# Costimulation loss enhances IL-2-driven Treg generation by PI3K-STAT3 inhibition in CNS autoimmunity

**DOI:** 10.1038/s44321-026-00431-7

**Published:** 2026-05-05

**Authors:** Kyung-Ho Nam, Gil-Ran Kim, Yu-Rim Kim, Young Nam Kwon, Sung-Min Kim, Je-Min Choi

**Affiliations:** 1 Department of Life Science, College of Natural Sciences, Hanyang University, Seoul, Republic of Korea; 2 Research Institute for Natural Sciences, Hanyang University, Seoul, Republic of Korea; 3 Department of Neurology, Kangbuk Samsung Hospital, Seoul, Republic of Korea; 4 Biomedical Research Institute, Department of Neurology, Seoul National University Hospital, Seoul, Republic of Korea; 5 Department of Neurology, Seoul National University, College of Medicine, Seoul, Republic of Korea; 6 Hanyang Institute of Bioscience and Biotechnology, Hanyang University, Seoul, Republic of Korea; 7 Research Institute for Convergence of Basic Sciences, Hanyang University, Seoul, Republic of Korea

**Keywords:** Immunology, Neuroscience

## Abstract

Costimulation blockade with CTLA-4 Ig (Abatacept) is a widely used strategy to suppress autoreactive T cells; however, its efficacy is often self-limiting due to concurrent depletion of regulatory T cells (Tregs), which depend on CD28 signaling for homeostasis. Here, we demonstrate that costimulation blockade paradoxically potentiates IL-2-driven Treg generation by selectively reprogramming intracellular cytokine signaling. We identified that IL-2 activates STAT3 only in the presence of TCR stimulation, as this pathway requires CD28-mediated PI3K–AKT signaling, which is abrogated by costimulation blockade. Consequently, CTLA-4 Ig uncouples STAT3 activation from IL-2 signaling while sparing STAT5, thereby enhancing TGF-β/Smad2/3 signaling to induce Foxp3 expression. This dual action—sustained STAT5 activation and increased Smad2/3 signaling—promoted robust Treg generation that ameliorated experimental autoimmune encephalomyelitis (EAE). Furthermore, we confirmed that this synergistic effect is conserved in human T cells from patients with multiple sclerosis (MS) upon CTLA-4 Ig and IL-2 cotreatment. Our findings suggest a strategy to expand the utility of CTLA-4 Ig therapy, providing a mechanistic rationale for combining costimulation blockade with IL-2 to restore immune tolerance in CNS autoimmunity.

## Synopsis

Costimulation blockade rewires IL-2 signaling by selectively inhibiting STAT3 while preserving STAT5, thereby enhancing Smad2/3-driven Foxp3 induction. This mechanism promotes Treg differentiation and improves immune regulation in CNS autoimmunity.

- STAT3 phosphorylation induced by IL-2 under TCR stimulation was selectively inhibited by CTLA-4 Ig, while Smad2/3 signaling was enhanced, promoting Treg differentiation.
- Clinical severity in the EAE model was synergistically reduced by combined CTLA-4 Ig and IL-2 treatment compared to monotherapy.
- Combination with IL-2 is proposed to extend the therapeutic applicability of CTLA-4 Ig to CNS autoimmune diseases by restoring Treg-mediated immune regulation.

## Abstract (web summary)

Costimulation blockade rewires IL-2 signaling by selectively inhibiting STAT3 while preserving STAT5, thereby enhancing Smad2/3-driven Foxp3 induction. This mechanism promotes Treg differentiation and improves immune regulation in CNS autoimmunity.

## Graphical abstract

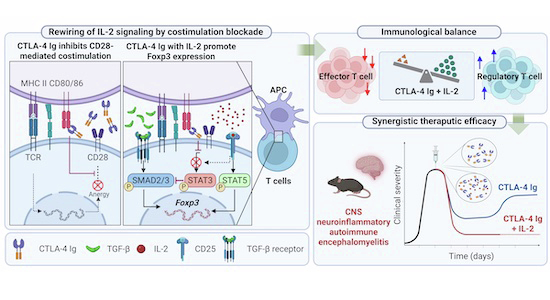

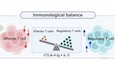

The paper explained **Problem**

Costimulation blockade with CTLA-4 Ig (e.g., Abatacept) suppresses autoreactive T cells but is limited by concurrent loss of regulatory T cells (Tregs), which depend on CD28 signaling for their maintenance. This paradox restricts its broader application beyond diseases such as rheumatoid arthritis and psoriatic arthritis.

**Results**

We show that costimulation blockade enhances IL-2–driven Treg differentiation by selectively rewiring intracellular signaling. CTLA-4 Ig disrupts CD28-dependent PI3K–AKT signaling, uncoupling STAT3 from IL-2 signaling while preserving STAT5. This shift favors TGF-β/Smad2/3–dependent Foxp3 induction, promoting Treg differentiation and amelioration of experimental autoimmune encephalomyelitis.

**Impact**

Our study shows that the Treg-limiting effects of CTLA-4 Ig (e.g., Abatacept) can be overcome by IL-2 co-treatment, reinforcing Treg induction while preserving CTLA-4 Ig-mediated inhibition of effector T cell activation. This provides a mechanistic rationale for combining costimulation blockade with IL-2 to enhance immune tolerance and improve therapeutic outcomes in CNS autoimmune diseases.

## Introduction

Cytolytic T-lymphocyte-associated antigen-4 (CTLA-4) is a key immune checkpoint molecule, which competitively binds to CD80 and CD86 with greater affinity than CD28 (Azuma et al, 1993; Krummel and Allison, 1995; Linsley et al, 1991). CTLA-4 is expressed on activated T cells and negatively regulates their activation and proliferation by inhibiting costimulatory signaling, thereby promoting T cell anergy (Jago et al, 2004). CTLA-4 is constitutively expressed in Treg cells, where it suppresses the costimulatory signals required for the activation of neighboring effector T cells (Read et al, 2000; Takahashi et al, 2000). CTLA-4 also limits antigen-presenting cell (APC) function by physically removing CD80/CD86 through trans-endocytosis, thereby restricting T cell responses (Kim et al, 2026; Qureshi et al, 2011). In addition, CTLA-4 signaling is critical for maintaining Treg stability and lineage integrity (Zappasodi et al, 2021). The evolutionarily conserved cytoplasmic domain of CTLA-4 contains an immunoreceptor tyrosine-based inhibitory motif (ITIM)-like sequence, which transmits negative signals to T cells by recruiting SH2 domain-containing protein tyrosine phosphatase 1 and 2 (SHP1/2) or PP2A (Baroja et al, 2002; Schneider and Rudd, 2000). Several cytoplasmic binding partners have been identified in relation to endocytosis, recycling, and negative signaling (Kim and Choi, 2022), including SHP1/2 (Lorenz, 2009), PP2A (Teft et al, 2009), PKC-η (Kim et al, 2021a; Kong et al, 2014), AP-1/2 (Bradshaw et al, 1997; Schneider et al, 1999), and LRBA (Lo et al, 2015). Notably, the cytoplasmic domain of CTLA-4 can regulate T cell activation and inflammatory responses independently of ligand engagement (Chikuma et al, 2005; Choi et al, 2006; Vijayakrishnan et al, 2004). We previously demonstrated that a cell-penetrating peptide-conjugated CTLA-4 cytoplasmic domain protein or peptide exhibited therapeutic effects in allergic asthma (Lim et al, 2017), arthritis (Choi et al, 2008), experimental autoimmune encephalomyelitis (EAE) (Kim et al, 2021a; Kim et al, 2021b; Lim et al, 2015), and psoriasis (Lee et al, 2023). The peptide also enhanced Treg generation both in vitro and in vivo.

CTLA-4-Ig (abatacept), a fusion protein comprising the extracellular domain of CTLA-4 conjugated to the Fc region of human immunoglobulin G, inhibits T cell activation by blocking CD80/CD86 and thereby disrupting CD28-mediated costimulatory signaling (Silva et al, 2015). CTLA-4 Ig was first approved by the FDA in 2005 for rheumatoid arthritis (RA) and later in 2017 for active psoriatic arthritis (PsA). Abatacept has also been approved for the prevention of acute graft–versus–host disease (GvHD) when combined with calcineurin inhibitors and methotrexate (Genovese et al, 2005; Mease et al, 2017; Vincenti, 2008; Watkins et al, 2021). However, its clinical efficacy has been limited in several other autoimmune diseases including multiple sclerosis (MS), in part due to its unintended reduction and functional impairment of Treg cells (Khoury et al, 2017; Parulekar et al, 2013; Sandborn et al, 2012). CD28 signaling is essential for maintaining Foxp3, the master transcription factor of Tregs (Salomon et al, 2000; Vogel et al, 2015). Consequently, the inability to sustain long-term immune tolerance due to inadequate Treg preservation remains a major limitation of CTLA-4 Ig therapy (Glatigny et al, 2019; Orban et al, 2014). Therefore, there is a critical need to develop therapeutic strategies that preserve the Treg compartment while maintaining the inhibitory function of CTLA-4 Ig on effector T cells.

As Treg cells constitutively express high levels of the IL-2 receptor α chain (CD25) (Sakaguchi et al, 1995), low-dose interleukin-2 (IL-2) has been explored as a strategy to selectively expand Treg cells in autoimmune diseases (Raeber et al, 2023; Webster et al, 2009). While IL-2 alone expands Tregs and CTLA-4 Ig suppresses effector T cells, whether their combination exerts synergistic effects in CNS autoimmunity and its mechanism remains poorly understood.

Therefore, we examined whether the combination of CTLA-4 Ig with IL-2 restores Treg cells while maintaining suppression of effector T cell activation in EAE and MS. Unexpectedly, we found that IL-2 signaling under TCR stimulation induces not only canonical STAT5 activation but also STAT3 phosphorylation, which is associated with Foxp3 destabilization. Costimulation blockade by CTLA-4 Ig prevented this non-canonical STAT3 activation while preserving STAT5 signaling, thereby reshaping IL-2-mediated responses toward enhanced Foxp3 induction. This signaling rewiring contributed to the therapeutic efficacy of CTLA-4 Ig plus IL-2 in EAE. Importantly, a similar synergistic effect on Treg generation and effector suppression was observed in T cells from patients with MS. Together, these findings provide a mechanistic rationale for combining costimulation blockade with IL-2 as a therapeutic strategy in CNS autoimmune disease.

## Results

### CTLA-4 Ig reduces Treg cells in autoimmune disease

**Figure 1:**
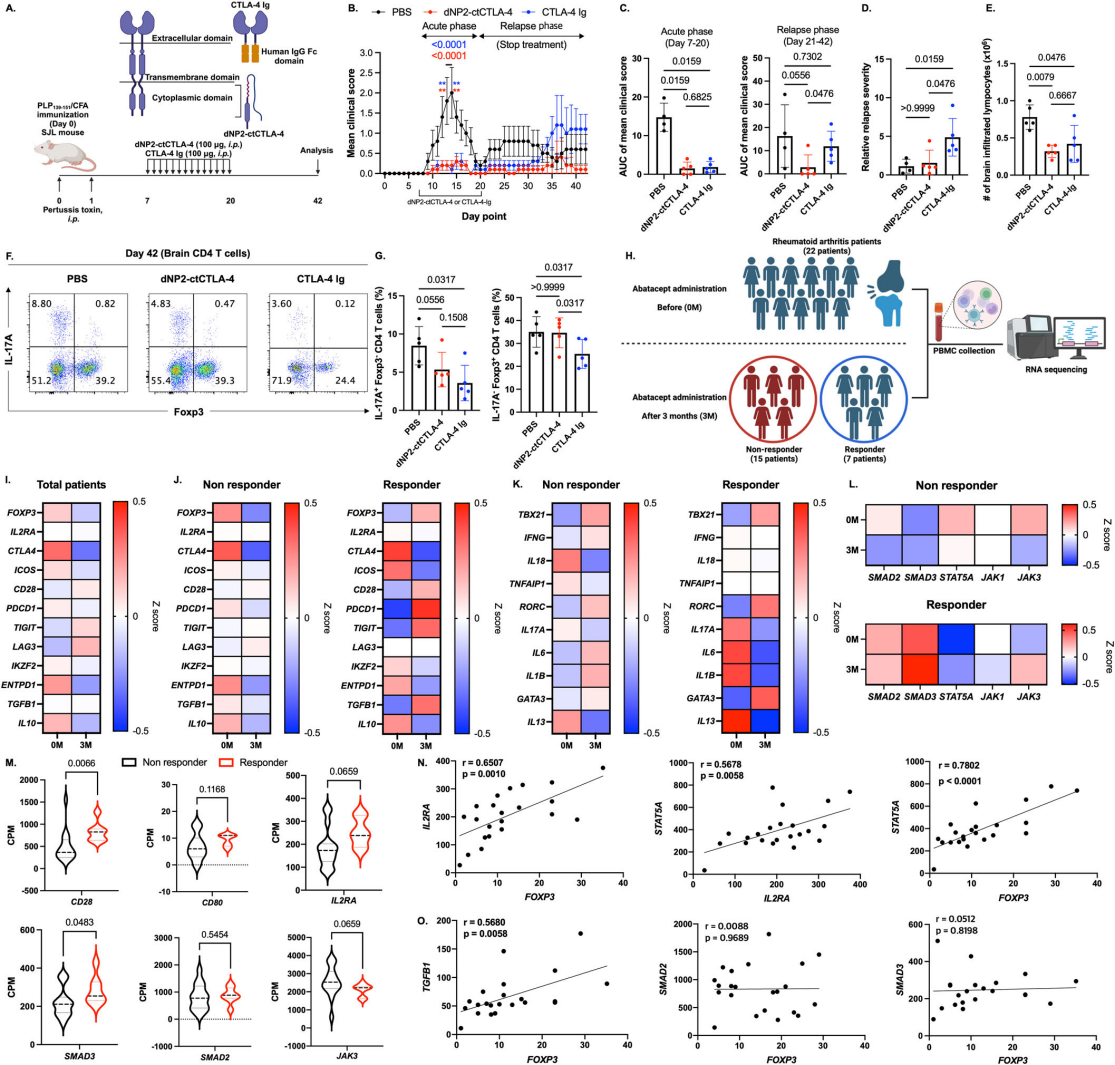
CTLA-4 Ig reduces Treg cells in autoimmune disease. (**A**–**G**) Relapsing-remitting experimental autoimmune encephalomyelitis (EAE) was induced by PLP_139-151_ in CFA emulsion with PTX. SJL mice were treated with CTLA-4 Ig (100 μg) and dNP2-ctCTLA-4 (100 μg) from day 7 to 20 (*n* = 5). (**A**) Experimental scheme used to induce EAE. (**B**) Clinical score of EAE. (**C**) Comparison of disease score AUC between peak stage and relapse stage. (**D**) Relative relapse severity. (**E**) The number of brain-infiltrated lymphocytes. (**F**, **G**) Proportion of brain-infiltrated IL-17A^+^ Foxp3^−^ and IL-17A^−^ Foxp3^+^ CD4 T cells. Representative FACS dot plot (**F**) and bar graph (**G**). (**H**–**O**) Gene expression comparison in PBMCs between before and after Abatacept treatment for 3 months in RA patients. (**H**) Schematic figure for comparing gene expression between patients. (**I**) Treg-related gene expression comparison between before and after Abatacept treatment for 3 months in total RA patients using heatmap. (**J**) Treg-related gene expression comparison between non-responder and responder patients using heatmap. (**K**) Inflammatory cytokine gene expression comparison utilizing heatmap between non-responders and responders. (**L**) Comparison of *SMAD2*, *SMAD3, STAT5A, JAK1* and *JAK3* gene expression between non-responders and responders before and after Abatacept treatment. (**M**) Comparison of *IL2RA, CD80, CD28*, *SMAD3*, *SMAD2* and *JAK3* expression between non-responders and responders before Abatacept treatment. (**N**) Correlation of *FOXP3* with *IL2RA*, *STAT5A* with *IL2RA* and *STAT5A* with *FOXP3* in total RA patients. (**O**) Correlation of *TGFB1* with *FOXP3*, *SMAD2* with *FOXP3* and *SMAD3* with *FOXP3*. Data are presented as mean ± SEM in (**B**) and as mean ± SD in the remaining panels. Statistical significance was determined by two-way ANOVA in (**B**) and nonparametric Mann–Whitney test in remaining panels. ns = nonsignificant, *P < 0.05, **P < 0.01, ***P < 0.001. Source data are available online for this figure.

To confirm the long-term consequences of CTLA-4 Ig treatment in CNS autoimmune disease, we utilized a relapsing-remitting EAE mouse model induced by proteolipid protein (PLP_139-151_) (Fig. 1A). We compared the effects by CTLA-4 Ig with dNP2-ctCTLA-4, a synthetic peptide composed of cytoplasmic domain of CTLA-4 conjugated to the cell-penetrating peptide dNP2 (Kim et al, 2021a). Although both molecules suppressed disease severity during the acute phase of treatment, only dNP2-ctCTLA-4 sustained its therapeutic effect and effectively prevented disease relapse after treatment cessation, whereas CTLA-4 Ig failed to maintain disease control (Fig. 1B,C). To quantify relapse, we defined a relative relapse severity score as the ratio of the post-relapse area under the curve (AUC) to the initial peak AUC. This parameter was selectively increased in the CTLA-4 Ig group, indicating aggravated disease re-exacerbation following withdrawal (Fig. 1D). The number of brain-infiltrated lymphocytes was reduced by both dNP2-ctCTLA-4 and CTLA-4 Ig (Fig. 1E), and the IL-17A expression in brain-infiltrated CD4 T cells was similarly decreased in both groups (Fig. 1F,G). However, a marked difference was observed in the Treg compartment. While dNP2-ctCTLA-4 maintained brain-infiltrated Foxp3^+^ CD4 Treg population for up to 22 days after treatment cessation, CTLA-4 Ig significantly reduced the proportion of these cells (Fig. 1F,G). These findings indicate that preservation of the Treg population may be associated with sustained long-term disease control.

**Figure EV1:**
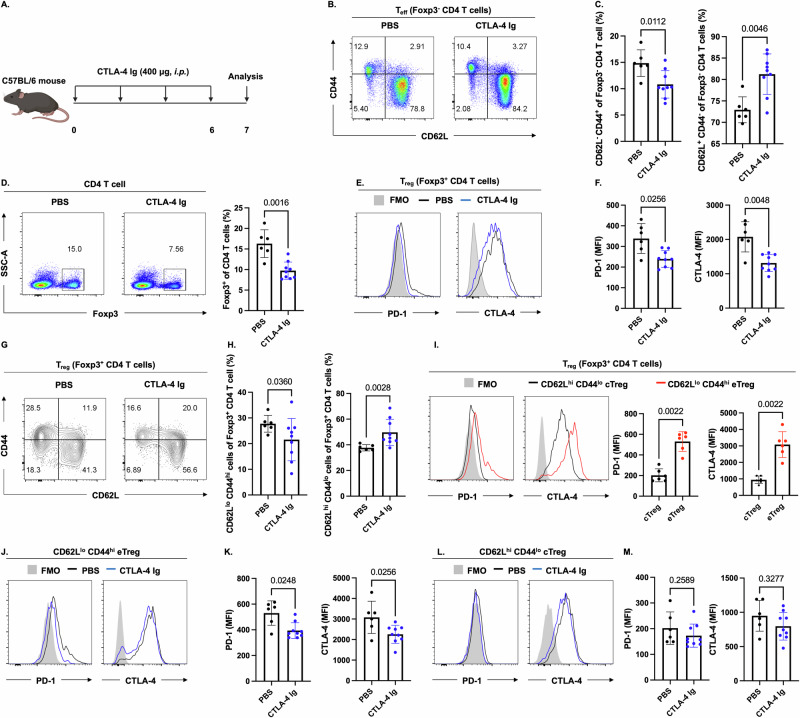
Costimulation blockade by CTLA-4 Ig reduces proportion of effector/memory CD4 T cells and eTreg cells in steady-state mice. (**A**–**M**) Intact C57BL/6 mice were intraperitoneally injected with 400 μg of CTLA-4 Ig every other day from day 0 to 6 and analyzed at day 7 (*n* = 6 (PBS), *n* = 9 (CTLA-4 Ig)). (**B**, **C**) Naive and effector/memory phenotypic change in CD4 T cells of spleen by CTLA-4 Ig analyzed in flow cytometry. (**D**) Expression of Foxp3 in CD4 T cells was analyzed by flow cytometry. (**E**, **F**) PD-1 and CTLA-4 expression in Foxp3^+^ CD4 T cells. (**E**) Representative histogram of CTLA-4 and PD-1. (**F**) Bar graph of MFI of PD-1 and CTLA-4. (**G**, **H**) CD62L^lo^ CD44^hi^ and CD62L^hi^ CD44^lo^ subpopulation of Foxp3^+^ CD4 T cells. (**I**) Comparison of Immuno-suppressive molecules, including PD-1 and CTLA-4 and expression in CD62L^lo^ CD44^hi^ Foxp3^+^ eTreg cells and CD62L^hi^ CD44^lo^ Foxp3^+^ cTreg cells were analyzed by flow cytometry. (**J**–**M**) Expression changes of PD-1 and CTLA-4 and expression in CD62L^lo^ CD44^hi^ Foxp3^+^ eTreg cells (**J**, **K**) and CD62L^hi^ CD44^lo^ Foxp3^+^ cTreg cells by CTLA-4 Ig (**L**, **M**). Data are collated from three independent experiments. Data are presented as mean ± SD. Statistical significance was determined by nonparametric Mann–Whitney test. ns = nonsignificant, *P < 0.05, **P < 0.01.

To determine whether this phenomenon reflects an impact of costimulatory blockade on Treg homeostasis, we examined steady-state mice treated with CTLA-4 Ig. CTLA-4 Ig treatment administered three times over 7 days led to a rapid decline in Foxp3^+^ CD4 T cells, accompanied by reduction in CD62L^lo^ CD44^hi^ effector CD4 T cells (Fig. EV1A–D). Expression of suppressive markers, including PD-1 and CTLA-4 (Fig. EV1E,F), was also diminished in Foxp3⁺ CD4 T cells, most prominently within the CD62L^lo^ CD44^hi^ effector Treg (eTreg) subset, which has more potent suppressive functions (Appendix Fig. S1A–D), whereas CD62L^hi^ CD44^lo^ central Treg (cTreg) cells were comparatively spared (Fig. EV1G–M). In thymic CD4 T cells, CTLA-4 Ig treatment reduced the frequency of thymic Foxp3^+^ Treg cells, without substantially altering Helios, PD-1, or CTLA-4 expression (Appendix Fig. S2A–C). Collectively, these findings suggest that costimulatory blockade constrains both peripheral maintenance and thymic generation of Treg cells.

Given that Abatacept is an FDA-approved therapy for rheumatoid arthritis (RA), we explored the translational relevance of these observations by reanalyzing an RNA-seq dataset of PBMCs from 22 RA patients treated with Abatacept for 3 months, available in the Dataset EV1 (10.1186/s13075-023-03236-y, Fig. 1H) (Iwasaki et al, 2024). Z-scores were calculated to visualize relative gene expression changes in heatmaps. Across the cohort, expression of Treg-related genes, including *FOXP3, PDCD1, CTLA4, ENTPD1* and *IL10*, was generally reduced following therapy, with a more pronounced decrease observed in non-responder patients (Fig. 1I,J). In contrast, responder patients exhibited greater reductions in inflammatory cytokine transcripts (*IL17A, IL6, IL1B, IL13*) (Fig. 1K). Notably, genes linked to upstream Foxp3 signaling, including *SMAD2, SMAD3* and *JAK3*, were expressed at lower levels in non-responder patients, indicating that intact TGF-β and IL-2 signaling is associated with therapeutic responsiveness (Fig. 1L,M). *CD28* expression was significantly lower in non-responder patients, suggesting that adequate *CD28* expression may further contribute to effective response (Fig. 1M). Regardless of Abatacept treatment status, *FOXP3, IL-2RA* and *STAT5A* expression showed strong positive correlations with each other (Fig. 1N). Moreover, *FOXP3* showed a positive correlation with *TGFB1* but not with *SMAD2* or *SMAD3* (Fig. 1O). As our bulk PBMC transcriptomic analysis lacks cell-type resolution and was not validated at the protein level in this cohort, these findings should be interpreted with caution. Consistent with our observations, prior studies have reported reduced expression of Foxp3 and suppressive molecules, including Helios, CD39, and CTLA-4, in PBMCs from Abatacept-treated RA patients (Aldridge et al, 2022; Pieper et al, 2013). These reports support the notion that costimulation blockade by CTLA-4 Ig is associated with alterations in the Treg compartment.

Collectively, these results suggest that although CTLA-4 Ig therapy effectively attenuates disease progression in both mice and humans, it concurrently imposes a constraint on the Treg compartment. These findings confirm a previously documented fundamental limitation of CTLA-4 Ig in achieving long-term immune regulation and underscore the need for complementary strategies that preserve IL-2 and TGF-β signaling to restore diminished Treg cell populations.

### CTLA-4 Ig and IL-2 synergistically increase Treg cell differentiation while suppressing effector T cell activation

**Figure 2:**
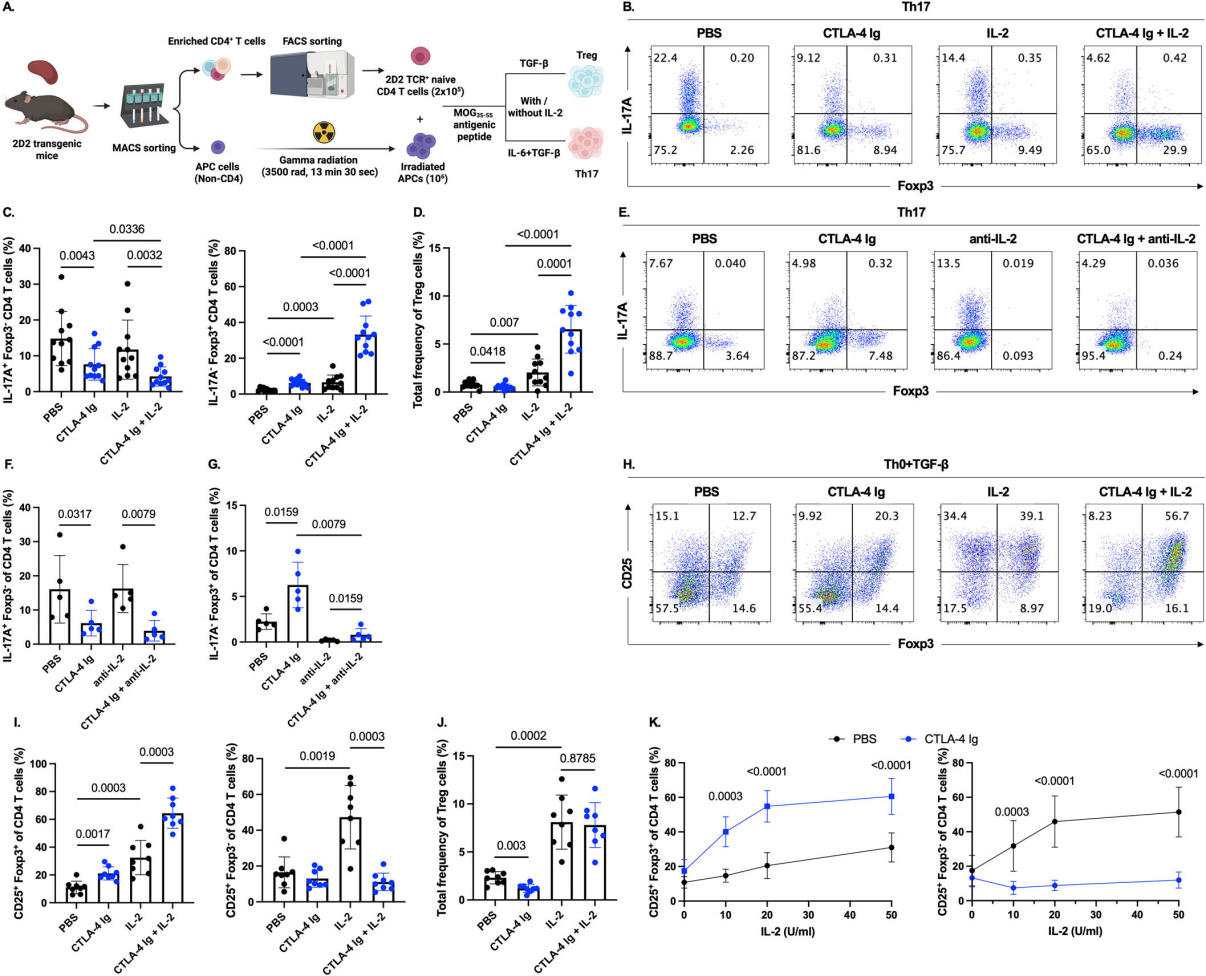
CTLA-4 Ig and IL-2 synergistically increase Treg cell differentiation while suppressing effector T cell activation. (**A**–**J**) Naive CD4 T cells sorted from 2D2 transgenic mice were cultured with irradiated APCs and stimulated by MOG_35-55_ antigenic peptide in presence (**B**–**G**) IL-6 + TGF-β (Th17) or (**H**–**J**) TGF-β (Th0+TGF-β), with or without 50 U/ml of IL-2, treatment of CTLA-4 Ig (0.5 µM) for 3 days. (**A**) Experimental flow of co-culture using FACS sorted naive 2D2 TCR^+^ CD4 T cells with APCs. (**B**) Relative FACS dot plot showing IL-17A and Foxp3 expression in CD4 T cells cultured with IL-2, with or without CTLA-4 Ig in Th17 polarized condition (*n* = 11). (**C**) Bar graph of IL-17A^+^ Foxp3^−^ effector CD4 T cells and IL-17A^−^ Foxp3^+^ iTreg cells. (**D**) Total frequency of IL-17A^−^ Foxp3^+^ iTreg cells based on flow cytometry. (**E**–**G**) Expression of IL-17A and Foxp3 in presence of CTLA-4 Ig with 5 μg/ml of anti-IL-2 neutralization antibody in Th17 polarized condition (*n* = 5). (**E**) Representative dot plot of IL-17A and Foxp3 expression in CD4 T cells. (**F**) Bar graph of IL-17A^+^ Foxp3^−^ CD4 T cells. (**G**) Bar graph of IL-17A^-^ Foxp3^+^ CD4 T cells. (**H**) Representative FACS dot plot showing CD25^+^ Foxp3^+^ proportion in CD4 T cells cultured with IL-2, with or without CTLA-4 Ig in Th0+TGF-β condition (*n* = 8). (**I**) Bar graph indicating CD25^+^ Foxp3^+^ Treg population and CD25^+^ Foxp3^-^ effector T cell population. (**J**) Total frequency of CD25^+^ Foxp3^+^ Treg cells based on flow cytometry. (**K**) IL-2 dose dependency in presence of TGF-β with CTLA-4 Ig (*n* = 5 per IL-2 concentration). Data are presented as the mean ± SD. Statistical significance was determined by nonparametric Mann–Whitney test. ns = nonsignificant, *P < 0.05, **P < 0.01. ***P < 0.001, ****P < 0.0001. Source data are available online for this figure.

To overcome the limitation of CTLA-4 Ig-induced Treg reduction, we next investigated whether IL-2 could compensate for this deficit. Given previous reports that reduced costimulation favors Treg differentiation with increased stability of Foxp3 expression (Mikami et al, 2020), we sought to determine whether costimulation blockade by CTLA-4 Ig could cooperate with IL-2 to promote Treg differentiation, while maintaining suppression of effector T cell activation. To validate this hypothesis, we utilized a co-culture system consisting of antigen-presenting cells (APCs) and naive 2D2 CD4 T cells (2D2/APC co-culture system), which express a T cell receptor specific for the myelin oligodendrocyte glycoprotein (MOG_35-55_) antigenic peptide (Fig. 2A). Under Th17-polarizing conditions (TGF-β + IL-6), CTLA-4 Ig treatment alone significantly inhibited IL-17A production while slightly increasing Foxp3 expression (Fig. 2B,C). IL-2 treatment alone did not affect IL-17A production but modestly increased Foxp3 expression (Fig. 2B,C). Interestingly, the combination of CTLA-4 Ig and IL-2 synergistically suppressed IL-17A production and markedly enhanced Foxp3 expression (Fig. 2B,C). However, in terms of the total frequency of Foxp3⁺ CD4 T cells, CTLA-4 Ig treatment alone reduced the Treg frequency (Fig. 2D). In contrast, co-administration of IL-2 significantly increased Treg frequency compared with either CTLA-4 Ig or IL-2 alone, accompanied by restoration of overall CD4 T cell proportions and viability (Appendix Fig. S3A–E). Moreover, in the presence of an anti-IL-2 neutralizing antibody, Foxp3 expression was no longer increased by CTLA-4 Ig treatment, while the inhibitory effect of CTLA-4 Ig on IL-17A production was preserved (Fig. 2E–G). These results indicate that IL-2 is required for Foxp3 induction in the context of CTLA-4 Ig treatment, whereas the suppression of Th17 effector function by CTLA-4 Ig occurs independently of IL-2. In addition, concomitant treatment with CTLA-4 Ig and IL-2 robustly induced CD25^+^ Foxp3^+^ CD4 T cells compared to treatment with IL-2 alone or CTLA-4 Ig alone, while the CD25^+^ Foxp3^-^ effector CD4 T cell population was significantly reduced under Treg-polarizing condition (TGF-β) (Fig. 2H,I). When analyzed as total frequency based on the entire gating strategy (Appendix Fig. S3F–J), CTLA-4 Ig reduced total frequency of Treg cells and the combination with IL-2 resulted in restoration of total Treg frequency comparable to IL-2 alone (Fig. 2J). The increase in Treg population was observed in an IL-2 dose-dependent manner, while CD25^+^ Foxp3^-^ effector CD4 T cells remained at low levels (Fig. 2K). However, no dose-dependent effect was observed with TGF-β (Appendix Fig. S4A–C). This pattern is the opposite of that observed with dNP2-ctCTLA-4, which exhibits a TGF-β dependent, rather than IL-2-dependent, effect on Treg induction (Appendix Fig. S5A–D). In addition to the co-culture system, we confirmed that, in the absence of CD28 stimulation, CD25⁺ Foxp3⁺ Treg cells were more efficiently induced in naive CD4 T cell–only cultures (Appendix Fig. S6A), with increased Foxp3 intensity and comparable CTLA-4 expression and proliferation (Appendix Fig. S6B). Collectively, these findings indicate that CTLA-4 Ig and IL-2 synergistically enhance Treg differentiation while maintaining suppression of effector T-cell activation, thereby counteracting CTLA-4 Ig-induced Treg reduction.

### Concomitant CTLA-4 Ig and IL-2 treatment synergistically ameliorates EAE disease

**Figure 3:**
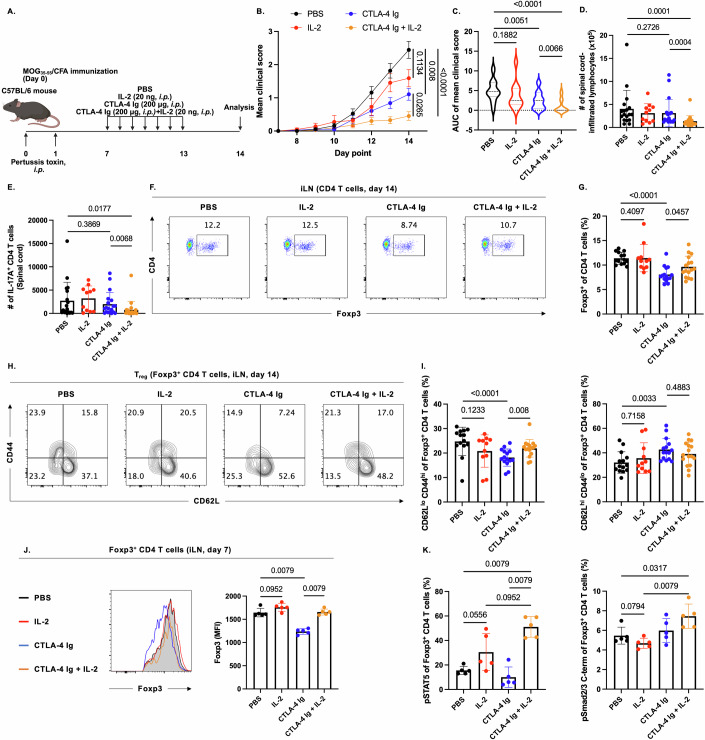
Concomitant CTLA-4 Ig and IL-2 treatment synergistically ameliorates EAE disease. (**A**–**I**) Ten-week-old C57BL/6 female mice were subcutaneously immunized with MOG_35-55_ peptide (100 µg /flank) emulsified in CFA together with PTX. From day 7 after disease induction, mice received CTLA-4 Ig (200 µg), IL-2 (20 ng) or a combination of CTLA-4 Ig and IL-2 until day 13. (Representative data from 4 independent experiments, *n* = 19 (PBS), *n* = 11 (IL-2), *n* = 19 (CTLA-4 Ig), *n* = 20 (CTLA-4 Ig + IL-2)). (**A**) Experimental scheme for inducing EAE. (**B**) Clinical disease score after EAE induction. (**C**) AUCs of total disease score after induction of EAE. (**D**) Cell number of spinal cord-infiltrated lymphocyte analyzed by flow cytometry. (**E**) The number of IL-17A producing CD4 T cells in spinal cord. (**F**, **G**) Proportion of Foxp3^+^ CD4 T cells in inguinal lymph node (iLN) analyzed by flow cytometry. Representative FACS dot plot (**F**) and bar graph (**G**). (**H**) Representative dot plot of naive (CD62L^hi^ CD44^lo^) and effector/memory (CD62L^lo^ CD44^hi^) T cells in Foxp3^+^ CD4 Treg cells of iLN analyzed by flow cytometry. (**I**) Bar graph indicating proportion of eTreg cells and cTreg cells. (**J**, **K**) Ten-week-old C57BL/6 female mice were subcutaneously immunized with MOG_35-55_ peptide (100 µg/ flank) emulsified in CFA with PTX. From day 0 after disease induction, mice received CTLA-4 Ig (200 µg), IL-2 (20 ng) or a combination of CTLA-4 Ig and IL-2 until day 7 (*n* = 5 per group). (**J**) Representative histogram and bar graph of Foxp3 in Foxp3^+^ CD4 T cells. (**K**) Bar graph indicating expression of pSTAT5 and pSmad2/3 C-term in Foxp3^+^ CD4 T cells. Data are presented as mean ± SEM in (**B**) and mean ± SD in remaining panels. Statistical significance was determined by two-way ANOVA in (**B**) and nonparametric Mann–Whitney test in remaining panels. ns = nonsignificant, *P < 0.05, **P < 0.01, ***P < 0.001, ****P < 0.0001. Source data are available online for this figure.

Given the cooperative effects of CTLA-4 Ig and IL-2 on Treg induction in vitro, we next examined whether this combination could improve functional disease control in vivo. To validate this hypothesis, we employed MOG_35-55_-induced EAE disease model. 200 μg of CTLA-4 Ig and 20 ng of IL-2 were intraperitoneally administered every day from day-7 to day-13 (Fig. 3A). Combination treatment resulted in a significantly lower clinical scores compared with all other groups, including CTLA-4 Ig monotherapy, as reflected by both mean clinical scores and the AUC analysis (Fig. 3B,C). The number of lymphocytes infiltrating the spinal cord was significantly reduced by the combined CTLA-4 Ig and IL-2 treatment compared to the control group (Fig. 3D). Consistently, the number of IL-17A-producing CD4 T cells was most markedly reduced in the combination treatment group (Fig. 3E). At a dose of 20 ng, IL-2 alone did not increase the frequency of Foxp3⁺ CD4 T cells, indicating that this dose was insufficient to promote Treg expansion (Fig. 3F,G). Notably, the proportion of Foxp3^+^ CD4 Treg cells, which were diminished by CTLA-4 Ig treatment, were restored when IL-2 was co-administered with CTLA-4 Ig in draining lymph nodes (Fig. 3F,G). In addition, the proportion of CD62L^lo^ CD44^hi^ eTreg cells, which were attenuated by CTLA-4 Ig, was significantly recovered with the addition of IL-2 (Fig. 3H,I). In contrast, no significant effect was observed on CD62L^hi^ CD44^lo^ cTreg cells by IL-2 supplementation to CTLA-4 Ig treatment (Fig. 3H,I). Mechanistically, Foxp3⁺ CD4 T cells in the draining lymph node from mice receiving combined CTLA-4 Ig and IL-2 treatment exhibited increased pSTAT5 and C-terminal Smad2/3 phosphorylation (pSmad2/3 C-term), accompanied by restoration of Foxp3 expression in the presence of CTLA-4 Ig (Fig. 3J,K; Appendix Fig. S7A,B), suggesting enhanced Treg differentiation-associated signaling events in EAE mice. Therefore, these results suggest that concomitant treatment with CTLA-4 Ig and IL-2 synergistically ameliorates EAE progression by promoting eTreg differentiation and enhancing IL-2 and TGF-β signaling, thereby restraining Th17 cell infiltration into the spinal cord.

### CTLA-4 Ig inhibits IL-2-driven PI3K/STAT3 axis, thereby enhancing Foxp3 expression

**Figure 4:**
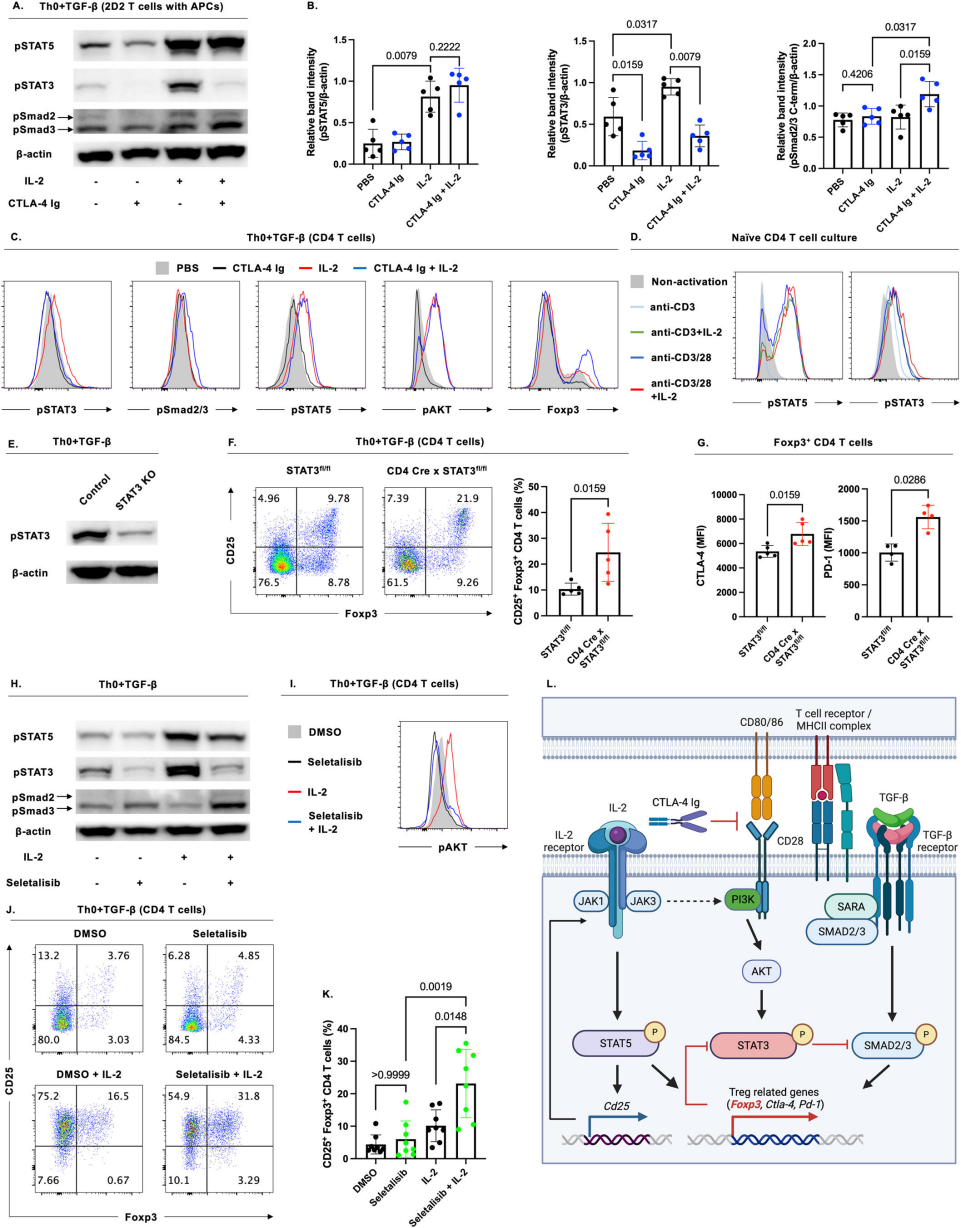
CTLA-4 Ig inhibits IL-2-driven PI3K/STAT3 axis thereby enhancing Foxp3 expression. (**A**–**C**) Naive CD4 T cells sorted from 2D2 transgenic mice were cultured with irradiated APCs and stimulated by 20 μg/ml of MOG_35-55_ antigenic peptide with 2 ng/ml of TGF-β (Th0+ TGF-β) or TGF-β with 50 U/ml of IL-2 in presence of CTLA-4 Ig (0.5 µM) for 3 days (*n* = 5). (**A**) Immunoblotting of phosphorylation of STAT5, STAT3, C-terminal (C-term) of Smad2 and Smad3. (**B**) Representative change in band intensity of pSTAT5, pSTAT3 and pSmad2/3 C-term. (**C**) Phosphorylation of STAT3, Smad2/3 C-term, STAT5 and AKT with expression of Foxp3 in CD4 T cells analyzed by flow cytometry. (**D**) FACS-sorted naive CD4 T cells were cultured with anti-CD3 (2 μg/ml), anti-CD3+anti-CD28 (5 μg/ml) with or without 50 U/ml of IL-2 for 3 days. The expression levels of pSTAT5 and pSTAT3. (**E**–**G**) Splenocyte from STAT3^fl/fl^ and CD4 Cre x STAT3^fl/fl^ mice were stimulated with a-CD3 (2 μg/ml) in presence of TGF-β (2 ng/ml) with or not IL-2 (50 U/ml) for 3 days (*n* = 5). (**E**) Immunoblotting of phosphorylation of STAT3 compared between STAT3 WT (STAT3^fl/fl^) and STAT3 KO (CD4 Cre x STAT3^fl/fl^). (**F**) Proportion of CD25^+^ Foxp3^+^ Treg cells in CD4 T cells in presence of TGF-β. (**G**) Expression of Foxp3 and PD-1 in Foxp3^+^ CD4 T cells in presence of TGF-β. (**H**–**K**) Splenocyte from C57BL/6 mice were stimulated by a-CD3 (2 μg/ml) with TGF-β (2 ng/ml) or TGF-β and IL-2 (50 U/ml) in presence of seletalisib (1 μM) for 3 days (*n* = 7). (**H**) Immunoblotting of phosphorylation of STAT5, STAT3 and Smad2/3 C-term. (**I**) Phosphorylation of AKT in CD4 T cells analyzed by flow cytometry. (**J**, **K**) Proportion of CD25^+^ Foxp3^+^ Treg cells in presence of seletalisib. Representative FACS dot pot (**J**) and bar graph (**K**) of proportion of CD25^+^ Foxp3^+^ Treg cells. (**L**) Schematic depiction of mechanism through which CTLA-4 Ig modulates IL-2-driven PI3K/STAT3 signaling. Data are presented as the mean ± SD. Statistical significance was determined by nonparametric Mann–Whitney test. ns = nonsignificant, *P < 0.05, **P < 0.01. ***P < 0.001. Source data are available online for this figure.

Next, to elucidate the molecular mechanisms underlying synergistic effects of the combined treatment of CTLA-4 Ig and IL-2 on Treg differentiation, we investigated intracellular signaling pathways related to IL-2 and TGF-β. In the 2D2/APC co-culture system stimulated with MOG_35-55_ antigenic peptide, IL-2 robustly induced STAT5 phosphorylation, which was unaffected by CTLA-4 Ig treatment (Fig. 4A,B). Unexpectedly, IL-2 also significantly increased pSTAT3 levels, which were dramatically inhibited by CTLA-4 Ig, with enhanced pSmad2/3 C-term (Fig. 4A,B). These findings were further confirmed by flow cytometry, which showed consistent results at the single-cell level, including a marked reduction of IL-2–induced pAKT upon CTLA-4 Ig treatment together with enhanced Foxp3 induction in the presence of IL-2 (Fig. 4C). Consistently, pSTAT3 levels were reduced by CTLA-4 Ig regardless of whether they were normalized to β-actin or to total STAT3 (Appendix Fig. S8A,B). To further define whether pSTAT3 is directly driven by IL-2 signaling and not by indirect signal from APCs, we utilized an APC-free system using FACS-sorted naive CD4 T cells stimulated with anti-CD3 or anti-CD3/CD28 antibodies in the presence or absence of IL-2 (Fig. 4D). IL-2 addition either to anti-CD3 or anti-CD3/28 antibody stimulation showed increased pSTAT3 expression compared to respective control group, with the highest pSTAT3 expression observed in the anti-CD3/CD28 + IL-2 condition (Fig. 4D). In contrast, pSTAT5 level were strongly induced by either IL-2 addition or anti-CD3/28 stimulation (Fig. 4D). In addition, IL-2 blockade by anti-IL-2 neutralizing antibody reduced expression of pSTAT3 as well as pSTAT5 (Appendix Fig. S9A). However, IL-2 alone was insufficient but required T cell receptor (TCR) stimulation to induce STAT3 phosphorylation (Appendix Fig. S9B).

**Figure EV2:**
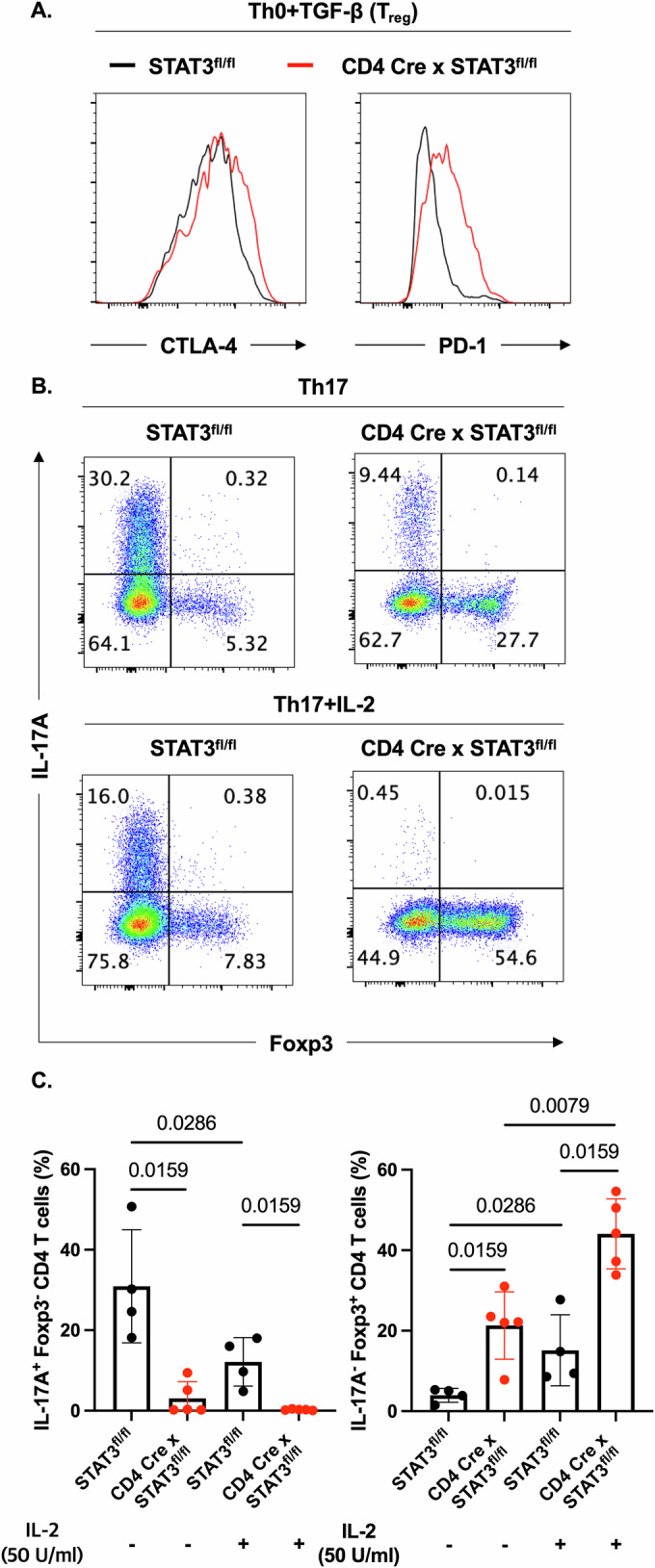
STAT3 deficiency enhances Foxp3 expression and suppresses Th17 differentiation in the presence of IL-2. (**A**) Representative histogram of CTLA-4 and PD-1 in Foxp3^+^ CD4 T cells in the presence of TGF-β condition. (**B**, **C**) Splenocyte from STAT3^fl/fl^ and CD4 Cre x STAT3^fl/fl^ mice were stimulated with a-CD3 (2 μg/ml) in presence of TGF-β (2 ng/ml) and IL-6 (30 ng/ml) with or not IL-2 (50 U/ml) for 3 days (*n* = 4 (STAT3^fl/fl^), *n* = 5 (CD4 Cre x STAT3^fl/fl^)). (**B**) Representative FACS dot plot and (**C**) bar graph of expression of IL-17A and Foxp3 in CD4 T cells. Data are presented as mean ± SD. Statistical significance was determined by nonparametric Mann–Whitney test. ns = nonsignificant, *P < 0.05, **P < 0.01.

Based on these observations, we hypothesized that pSTAT3 may negatively regulate C-term pSmad2/3, thereby limiting TGF-β-dependent Foxp3 expression. To test this, splenocytes from CD4 Cre x STAT3^flox/flox^ (CD4 Cre x STAT3^fl/fl^) mice were analyzed to determine whether Foxp3 expression is enhanced in the absence of STAT3. As shown in Fig. 4E, we confirmed that STAT3 phosphorylation was completely absent in STAT3-deficient cells. Importantly, there was a significant increase in CD25⁺ Foxp3⁺ cells in STAT3-deficient CD4 T cells, along with increased expression of CTLA-4 and PD-1 (Figs. 4F,G and EV2A). In addition, under Th17 polarizing condition, STAT3 deficient CD4 T cells showed markedly reduced IL-17A production and enhanced Foxp3 expression in presence of IL-2 (Fig. EV2B,C), mirroring the phenotype observed with CTLA-4 Ig (Fig. 2). These findings prompted us to examine whether upstream PI3K/AKT signaling contributes to IL-2–driven STAT3 activation under costimulatory conditions. Given prior evidence that IL-2 can activate the PI3K/AKT pathway (Johnston et al, 1995) and that PI3K/AKT signaling may facilitate STAT3 phosphorylation (Fung et al, 2003), we next assessed the effect of pharmacologic PI3K inhibition using PI3K inhibitor (Seletalisib). PI3K inhibition resulted in a significant reduction of pSTAT3 with marked increase of pSmad2/3 C-term (Fig. 4H), together with diminished pAKT induced by IL-2 (Fig. 4I). Moreover, treatment with Seletalisib and IL-2 further increases the proportion of CD25^+^ Foxp3^+^ Treg cells (Fig. 4J,K), consistent with the results observed with CTLA-4 Ig and IL-2.

Collectively, these results support a mechanistic model in which CTLA-4 Ig suppresses IL-2-driven PI3K/STAT3 signaling under TCR stimulation while enhancing TGF-β/Smad2/3 pathway, thereby synergistically promoting Foxp3 expression and Treg cell differentiation (Fig. 4L).

### Clinical impact of concomitant treatment of CTLA-4 Ig and IL-2 to modulate Treg subpopulations in multiple sclerosis

**Figure 5:**
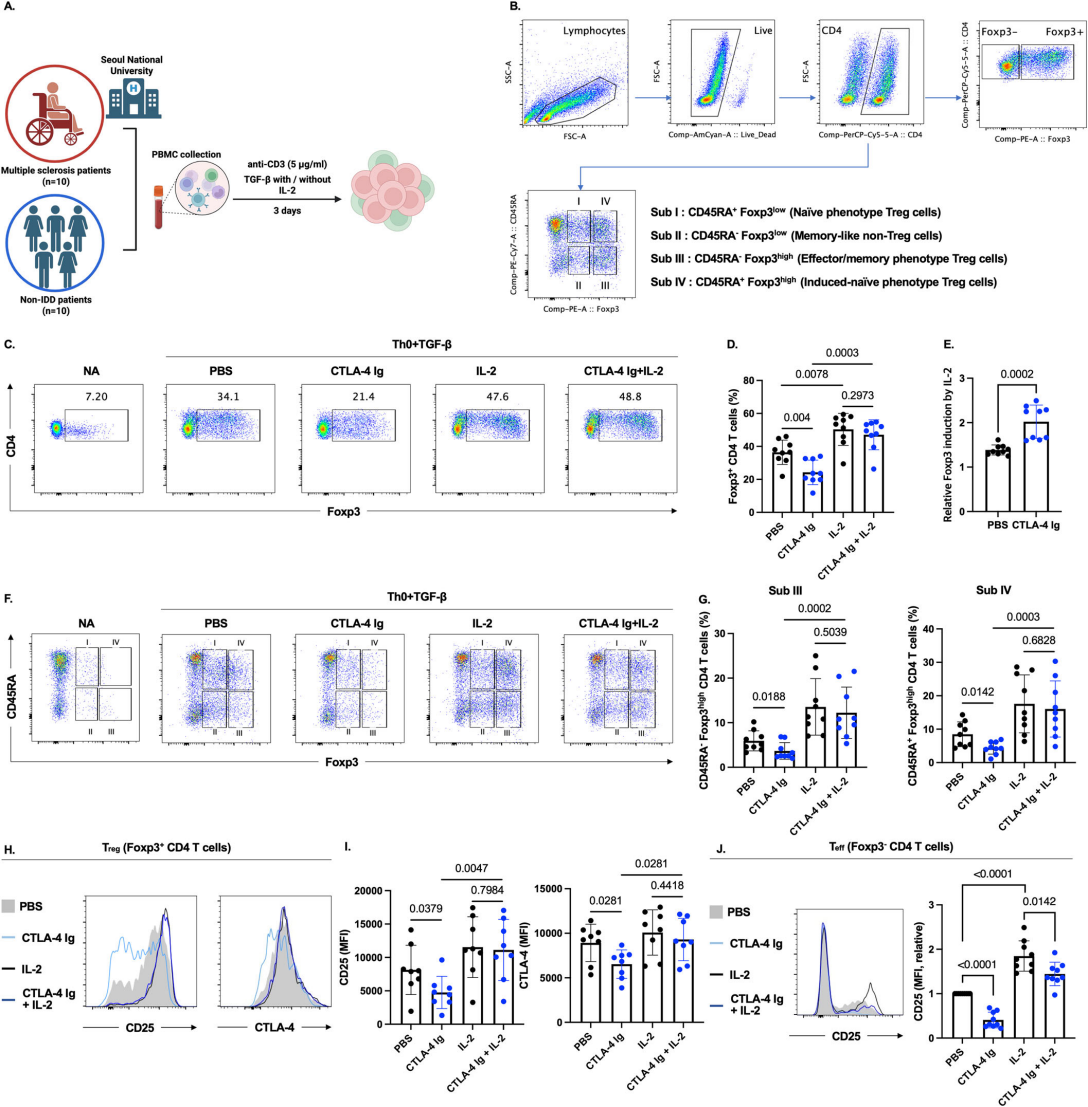
Clinical impacts of concomitant treatment of CTLA-4 Ig and IL-2 to modulate Treg subpopulation in multiple sclerosis. (**A**–**J**) PBMCs from MS patients were stimulated with human anti-CD3 monoclonal antibody under 2 ng/ml of TGF-β with or without 50 U/ml of IL-2 in presence of 1 µM of CTLA-4 Ig for 3 days (*n* = 8–9 independent human donors). (**A**) Schematic overview of the experimental design and (**B**) gating strategy for Treg subset analysis. Naive and effector/memory Treg cells were analyzed in 4 subpopulations; CD45RA^+^ Foxp3^low^ Treg cells: Sub I; CD45RA^-^ Foxp3^low^ Treg: Sub II; CD45RA^-^ Foxp3^high^ Treg cells: Sub III; CD45RA^+^ Foxp3^high^ Treg cells: Sub IV. (**C**, **D**) Representative FACS dot plot (**C**) and bar graph (**D**) exhibits Foxp3 induction. (**E**) IL-2 induced Foxp3 ratio with or without CTLA-4 Ig. (**F**, **G**) Representative FACS dot plot (**F**) and bar graph (**G**) of III (Effector/memory phenotype Treg cells) and IV (induced-naive phenotype Treg cells) sub-population of Treg cells. (**H**) Representative histogram (**I**) and bar graph of CD25 and CTLA-4 expression in Foxp3^+^ CD4 T cells. (**J**) Representative histogram and bar graph of CD25 expression in Foxp3^−^ effector CD4 T cells. Data are presented as the mean ± SD. Statistical significance was determined by nonparametric Mann–Whitney test. ns = nonsignificant, *P < 0.05, **P < 0.01, ***P < 0.001, ****P < 0.0001. Source data are available online for this figure.

**Figure EV3:**
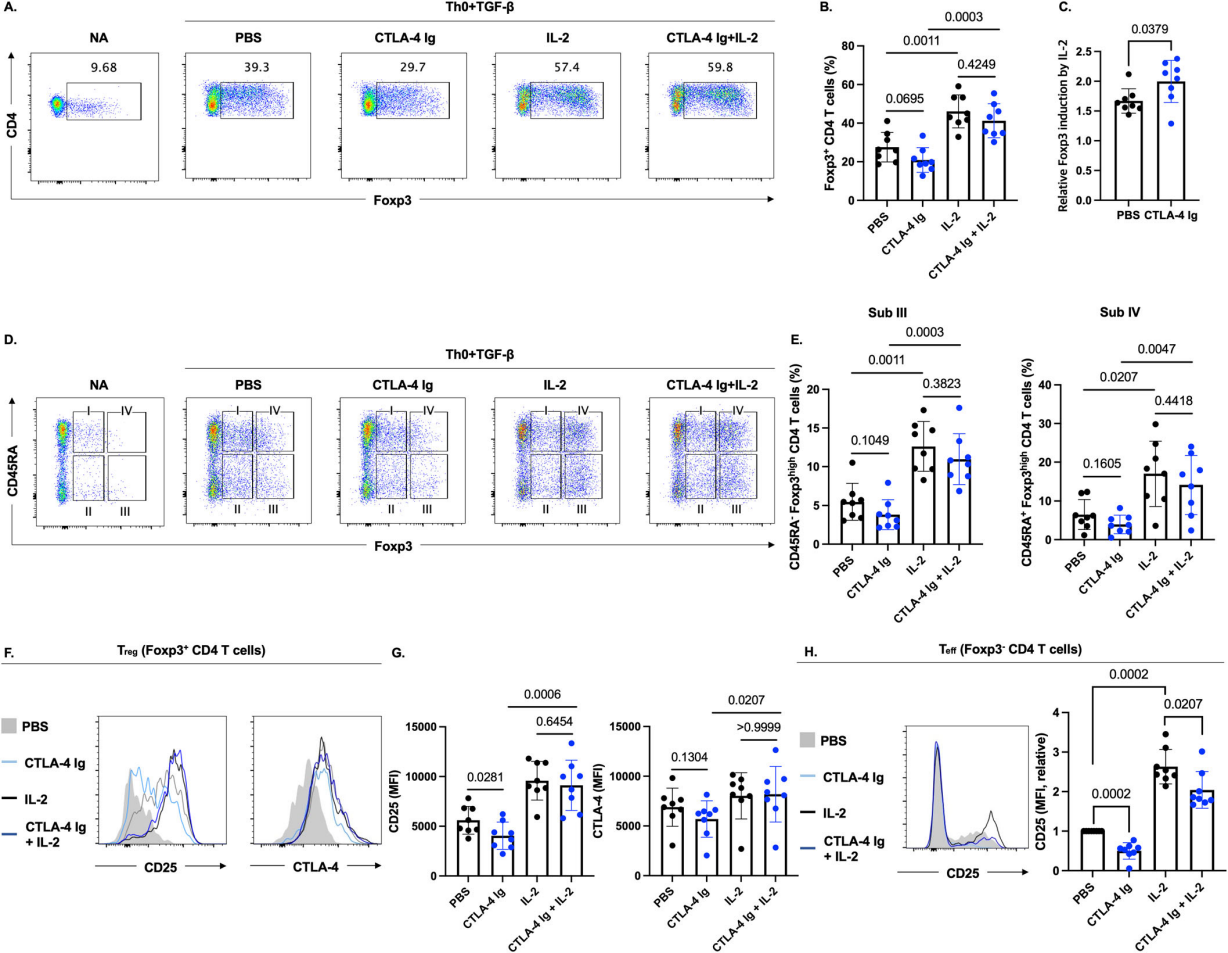
CTLA-4 Ig and IL-2 co-treatment increases Foxp3^hi^ Treg subpopulations and enhances suppressive molecule expression in human PBMCs from non-IDD patients. (**A**–**H**) PBMCs from non-IDD patients were stimulated with human anti-CD3 monoclonal antibody under 2 ng/ml of TGF-β with or without 50 U/ml of IL-2 in presence of 1 µM of CTLA-4 Ig for 3 days (*n* = 8 from independent donors). (**A**, **B**) Representative FACS dot plot (**A**) and bar graph (**B**) exhibits Foxp3 induction. (**C**) IL-2 induced Foxp3 ratio with or without CTLA-4 Ig. (**D**, **E**) Representative FACS dot plot (**D**) and bar graph (**E**) of III (Effector/memory phenotype Treg cells) and IV (induced-naive phenotype Treg cells) sub-population of Treg cells. (**F**) Representative histogram (**G**) and bar graph of CD25 and CTLA-4 expression in Foxp3^+^ CD4 T cells. (**H**) Representative histogram and bar graph of CD25 expression in Foxp3^−^ effector CD4 T cells. Data are presented as the mean ±  SD. Statistical significance was determined by nonparametric Mann–Whitney test. ns = nonsignificant, *P < 0.05, **P < 0.01, ***P < 0.001.

Lastly, to assess the translational relevance of our findings, we evaluated the effects of CTLA-4 Ig in combination with IL-2 on human PBMCs from relapsing-remitting multiple sclerosis (RRMS) patients and non-inflammatory demyelinating disease (non-IDD) patients (Fig. 5A). We first analyzed changes in Foxp3 expression in the presence of CTLA-4 Ig or IL-2, and then examined the combined effects of CTLA-4 Ig and IL-2 in Treg subpopulation defined by CD45RA and Foxp3 by flow cytometry (Fig. 5B). PBMCs were stimulated with a soluble anti-CD3 antibody in the presence of TGF-β for 3 days, resulting in a significant induction of Foxp3 in CD4 T cells (Fig. 5C). Consistent with our previous results, CTLA-4 Ig treatment led to a reduction in the total Foxp3 proportion within CD4^+^ T cells; however, this effect was completely reversed and even further enhanced-upon co-administration of IL-2 (Fig. 5C,D). Interestingly, Foxp3 induction by IL-2 was more pronounced in the presence of CTLA-4 Ig, suggesting that IL-2-mediated Foxp3 induction is enhanced under conditions of reduced costimulatory signaling (Fig. 5E). To further dissect the human Treg subpopulations, we classified them based on Foxp3 and CD45RA expression within CD4^+^ T cells (Fig. 5F; Appendix Fig. S10A) following a previously established classification system (Miyara et al, 2009). In our dataset, we identified an additional distinct subpopulation (Sub IV), indicative of an induced-naive phenotype Treg (inTreg) population driven by Treg-inducing cytokine stimulation. Thus, our analysis revealed three functional Treg subpopulations (Sub I, III, IV). Among the Foxp3^high^ populations (Sub III and Sub IV), the CD45RA^-^ Foxp3^high^ effector Treg subset exhibited higher expression of Treg-related markers compared to other subpopulations, including CD45RA^+^ Foxp3^high^ inTreg cells (Appendix Fig. S10B). In RRMS patients’ PBMCs, IL-2 strongly induced Foxp3 expression at high intensity regardless of CTLA-4 Ig treatment, suggesting that IL-2 enhances both naive and effector Treg expansion (Fig. 5F,G), while having minimal effects on the Foxp3^low^ population (Sub I and Sub II; Appendix Fig. S10C). Moreover, the diminished expression of suppressive molecules, including CD25 and CTLA-4, caused by CTLA-4 Ig, was significantly restored by IL-2 (Fig. 5H,I). In Foxp3^−^ effector CD4^+^ T cells, CTLA-4 Ig reduced CD25 expression even in presence of IL-2 (Fig. 5J). These patterns are also confirmed in PBMCs from patients with non-inflammatory demyelinating disease (non-IDD) (Fig. EV3A–H).

Collectively, our murine findings were recapitulated in human PBMCs, indicating that reduced costimulatory signaling in the presence of IL-2 preferentially supports Treg differentiation while preserving suppression of effector T-cell activation. This combined treatment promoted a tolerogenic immune phenotype characterized by enrichment of Foxp3^high^ functional Treg subpopulations and increased expression of key suppressive markers, including Foxp3, CD25, and CTLA-4, highlighting the potential translational relevance of concomitant CTLA-4 Ig and IL-2 combination therapy in multiple sclerosis.

## Discussion

In this study, we demonstrate that attenuation of costimulatory signaling by CTLA-4 Ig constrains the Treg compartment, whereas IL-2 supplementation restores this deficit by increased Treg differentiation, and improves immune regulation in both murine EAE model and human MS patients. Unexpectedly, we found that IL-2 signaling activates STAT3 via the PI3K/AKT pathway in the presence of TCR stimulation, a pathway capable of limiting TGF-β-dependent Foxp3 induction. Importantly, CTLA-4 Ig selectively inhibited IL-2-driven STAT3 phosphorylation while preserving STAT5 activation and enhancing TGF-β/Smad2/3 signaling, thereby shifting intracellular signaling toward a Treg differentiation-favoring state. Therefore, we propose IL-2 combination therapy with CTLA-4 Ig to compensate costimulation loss-induced Treg diminution.

MS is a neuroinflammatory autoimmune disease triggered and progressed by T cell- and B cell-mediated immune response against self-antigens, particularly proteins derived from the central nervous system (Cavallo, 2020). Several drugs targeting T cells and B cells, including fingolimod, natalizumab, and ocrelizumab, have been approved and are currently used for the treatment of MS (Jeffery et al, 2016; McCormack, 2013; Mulero et al, 2018). However, neither natalizumab nor fingolimod provides a permanent cure, as disease relapse occurs once treatment is discontinued (Fuentes-Rumí et al, 2020; Malpas et al, 2022), because as antigen-specific T cells cannot be removed completely. Importantly, in MS patients, both the number and function of Treg cells are compromised, while Th1 and Th17 cells are elevated, disrupting the balance between pathogenic effector T cells and Treg cells, and this imbalance is closely linked to disease severity, progression, and relapse (Jamshidian et al, 2013; Peelen et al, 2011; Zhang et al, 2021). The most prominent strategy to modulate MS has been thought to inhibiting T cell activation or Th1/Th17 cell functions. IL-17A blockade (e.g., ustekinumab, secukinumab, and RORγt inhibitors) (Confavreux and Compston, 2009), Th1-modulating therapies such as interferon-β (Kougkas et al, 2022), and agents that inhibit T-cell activation, including glatiramer acetate (Tselis et al, 2007), have been explored in this context. Although Th17 cells and related molecules, including RORγt, IL-17, IL-23, and GM-CSF, have been extensively explored as therapeutic targets, none have yet been approved for the treatment of MS. Compared with CAR-Treg therapy, low-dose IL-2 represents a more accessible and economically feasible approach, and in contrast to short-chain fatty acids, it more directly targets the IL-2 signaling axis critical for Treg survival and expansion. However, despite these advantages, low-dose IL-2 lacks complete selectivity, as clinical studies in autoimmunity have reported concomitant increases in NK cells and effector T cells (He et al, 2016; Koreth et al, 2011). This limitation underscores the need for approaches that both enhancing Treg function and directly inhibition of pathogenic effector T cell activation.

CTLA-4 Ig (Abatacept) is a recombinant fusion protein composed of the extracellular domain of CTLA-4 linked to the Fc portion of human IgG. It suppresses T cell activation by attenuating CD28-mediated costimulatory signaling and was approved by the U.S. FDA for rheumatoid arthritis (RA) in 2005 and for psoriatic arthritis (PsA) in 2017. A related molecule, Belatacept, which contains two amino-acid substitutions that increase affinity for CD86, was approved in 2011 for kidney transplantation (Kim et al, 2024; Vincenti et al, 2016). Accordingly, CTLA-4 Ig–based therapies are currently used primarily in RA and organ transplantation. Costimulatory blockade by Abatacept and Belatacept represents a rational strategy to suppress antigen-specific T cell activation and promote anergy, thereby potentially reducing pathogenic autoreactive clones. However, despite its clinical potency in RA and transplantation, CTLA-4 Ig has shown limited efficacy in other autoimmune diseases and cannot be expanded to human disease treatment, including MS. A phase II study (NCT01116427) involving 65 RRMS patients tested CTLA-4 Ig but found that treatment led to a reduction in Treg cells within PBMCs, specifically affecting effector CD45RO^+^ Treg cells, while sparing CD45RA^+^ Treg cells (Glatigny et al, 2019). No significant differences were observed in MRI findings or clinical parameters of disease activity (Khoury et al, 2017). Therefore, optimal therapeutic strategy with CTLA-4 Ig for MS would require suppression of pathogenic effector T cell activation with maintaining Treg cell numbers and function.

In recent years, CTLA-4 Ig has been explored in combination with T-cell receptor–targeting immunosuppressants, such as calcineurin inhibitors or mTOR inhibitors (e.g., Rapamycin; NCT06055608), with the aim of achieving stronger suppression of T-cell activation. While these strategies focus primarily on amplifying immunosuppression, they do not directly address the restoration of immune regulation mediated by Treg cells. Our transcriptomic analysis of RA patients who received CTLA-4 Ig for 3 months revealed that responders exhibited higher *CD28* and maintained *FOXP3* gene expression while non-responders showed reduced *FOXP3* compared to before treatment showing correlative results with previous reports. Strong positive correlation between *FOXP3* expression and CD25/STAT5-associated genes were observed in RA patients with CTLA-4 Ig treated group. In parallel, PBMC-derived Treg cells from MS patients exhibit impaired IL-2/STAT5 signaling compared with those from healthy individuals (Cerosaletti et al, 2013). In line with these observations, it is well established that both costimulatory signaling and IL-2 are essential for the induction, maintenance, and functional stability of Treg cells (Salomon et al, 2000; Shouse et al, 2024). Costimulatory signaling, particularly through CD28, plays a critical role in sustaining Treg lineage stability by maintaining Foxp3 expression and supporting IL-2 receptor–mediated survival pathways. In the absence of CD28 signaling, impaired thymic Treg development and reduced peripheral Treg survival have been reported, accompanied by decreased IL-2 mRNA levels and diminished CD25 expression under steady-state conditions (Zhang et al, 2013). Notably, Treg-specific conditional deletion of CD28 results in a marked reduction of CD25⁺ Foxp3⁺ Treg populations in the thymus, lymph nodes, and spleen, and is associated with the spontaneous development of systemic autoimmunity and skin inflammation (Tang et al, 2003). Therefore, CD28-mediated costimulation is an essential factor for long-term Treg maintenance. Conversely, although CD28-mediated costimulation is essential for long-term Treg maintenance, its attenuation is advantageous during the Treg induction phase, where reduced costimulatory signaling favors Treg differentiation by permitting stable FOXP3 locus demethylation and epigenetic imprinting, as demonstrated in studies of Treg induction and chromatin remodeling in the presence of TGF-β and IL-2 (Mikami et al, 2020). Consistent with this concept, combination strategies employing costimulation blockade together with IL-2–based interventions have shown superior therapeutic efficacy in autoimmune models, including type 1 diabetes (Wang et al, 2022). Taken together, these observations provide a rationale for why IL-2–mediated restoration of Treg cell abundance and function is more effective than CTLA-4 Ig monotherapy.

A recent study similar demonstrated that costimulation blockade with an anti-CD80/86 antibody significantly reduced Foxp3⁺ CD4 T cells in BALB/c mice, while concomitant treatment with an IL-2:anti-IL-2 antibody complex (IL-2c), which has preferential affinity for the IL-2 receptor α chain and promotes selective Treg expansion, effectively restored the Treg population and ameliorated autoimmune type 1 diabetes (Wang et al, 2022). Similar to our findings, this study highlighted Treg contraction as a critical limitation of costimulation blockade therapy and attempted to compensate for this defect using IL-2–based intervention. However, their strategy primarily relied on Treg expansion and maintenance, achieved through IL-2c administration that selectively increased Treg numbers with minimal direct impact on effector T cells. In contrast, although our approach also utilized IL-2, the underlying mechanism differs conceptually from the previous study. Rather than focusing solely on numerical expansion, we demonstrate that IL-2, in the context of limited costimulatory signaling, promotes potent differentiation of naive CD4 T cells into the functional Treg cells during effector T cell lineage inhibition. Notably, we observed that CTLA-4 Ig-induced reduction of CD62L^lo^ CD44^hi^ effector Treg cells was selectively restored by IL-2 co-administration. These restored Tregs exhibited elevated expression of PD-1 and CTLA-4, consistent with a highly suppressive phenotype. As IL-2 induced expansion would not affect CD62L or CD44 expression, increased CD44^hi^ to CD44^low^ Treg ratio implies an increased proportion of induced Treg cells. Importantly, CD25⁺ Foxp3⁻ non-Treg cells were strongly reduced indicating that effector T cell activation suppressed under CTLA-4 Ig treatment despite in the presence of IL-2. In the Wang et al study, the IL-2c-only group displayed the highest Treg proportion in an antigen-specific type 1 diabetes model, whereas combined costimulation blockade with IL-2c produced comparable or slightly lower Treg frequencies but yielded superior disease control. In contrast, our study employed a suboptimal dose of uncomplexed IL-2 did not expand Treg cells (20 ng, Fig. 3), under which IL-2 monotherapy exerted minimal effects on EAE progression and draining lymph node Treg proportions. Only the combination of CTLA-4 Ig with IL-2 resulted in both robust disease suppression and a significant increase in Treg numbers. Together, these comparisons suggest that while IL-2–based expansion strategies can restore Treg abundance, the combination of costimulation blockade with appropriately titrated IL-2 may additionally enhance qualitative Treg differentiation and functional potency, thereby achieving a more balanced immune regulation that simultaneously limits effector T cell activation and reinforces regulatory control. Importantly, Treg differentiation in vivo appears to be antigen-licensed (Josefowicz et al, 2012). Accordingly, in the EAE context, the presence of antigenic stimulation in draining lymph nodes likely enables selective enhancement of antigen-experienced Treg differentiation under CTLA-4 Ig and IL-2 co-treatment. Such antigen-contextualized differentiation may contribute to enhanced suppressive potency, and this framework suggests that this combination therapy biases differentiation toward antigen-relevant, functionally superior Treg subsets.

Mechanistically, we unexpectedly found IL-2 induced STAT3 activation as well as STAT5 activation during T cell activation or differentiation. Although STAT3 is typically activated by cytokines such as IL-6, IL-10, IL-21, and IL-23 through JAK-mediated signaling, earlier studies reported that IL-2 can also induce STAT3 phosphorylation in human T lymphocytes (Johnston et al, 1995), and that this effect is mediated through PI3K/AKT pathway (Fung et al, 2003; Karnitz et al, 1995). However, in our system, IL-2 alone was insufficient to induce detectable STAT3 phosphorylation, despite robust activation of STAT5. Instead, STAT3 phosphorylation occurred only when IL-2 stimulation was combined with TCR engagement specifically CD28-mediated costimulation. This indicates that IL-2 does not act as an autonomous STAT3 activator but amplifies STAT3 signaling in an antigen-driven activation context. Consistent with this model, CTLA-4 Ig selectively attenuated IL-2-driven PI3K/AKT and STAT3 activation while preserving STAT5 phosphorylation, thereby shifting intracellular signaling toward enhanced TGF-β/Smad2/3 activity and Foxp3 induction. This reciprocal regulation is consistent with prior reports describing antagonistic crosstalk between STAT3 and TGF-β/Smad2/3 signaling. For example, Wang et al showed that activated STAT3 can directly interact with Smad3 and attenuate TGF-β–induced responses by impairing Smad3-dependent transcriptional activity (Wang et al, 2016). In addition to this direct interaction, STAT3 has also been reported to upregulate the inhibitory Smad, Smad7, which in turn interferes with TGF-β receptor–mediated phosphorylation of Smad2 and Smad3, thereby dampening canonical TGF-β/Smad signaling (Kawabata et al, 1997; Luwor et al, 2013). In line with this model, our data suggest that CTLA-4 Ig-mediated costimulation blockade suppresses the PI3K/AKT–STAT3 axis, thereby relieving STAT3-dependent antagonism of TGF-β/Smad2/3 signaling and promoting Foxp3 induction and Treg differentiation. Pharmacologic PI3Kδ inhibition recapitulated key aspects of this signaling shift, including reduced pAKT and pSTAT3 levels with concomitant enhancement of Smad2/3 phosphorylation and Foxp3 induction. However, global PI3K inhibition lacks cellular selectivity and carries a higher risk of systemic immune perturbation (Tarantelli et al, 2021). In contrast, CTLA-4 Ig provides a T-cell–selective means of attenuating PI3K-dependent STAT3 signaling while preserving IL-2/STAT5 activity, thereby offering a mechanistically similar but clinically safer partner for IL-2 co-therapy. This distinction underscores the translational advantage of biologic costimulation blockade over broad kinase inhibition.

Meanwhile, translating these findings into clinical application requires careful consideration as human Treg biology is more complex than that of mice. Unlike murine systems, human Treg cells are composed of heterogeneous subpopulations that can be classified based on CD45RA and Foxp3 expression (Miyara et al, 2009). In this framework, Sub I (CD45RA^+^ Foxp3^low^) cells and Sub III (CD45RA^-^ Foxp3^high^) cells correspond to resting Treg (rTreg) and activated Treg (aTreg) cells, respectively, both of which exhibit suppressive function and serve as an important reference for understanding human Treg heterogeneity. In our in vitro differentiated human CD4 T cells demonstrated not only Sub I and Sub III populations but also a distinct Sub IV (CD45RA^+^ Foxp3^high^) observed by IL-2 treatment. This subset appeared to be preferentially induced by IL-2 and may represent IL-2/STAT5 axis-based increase of Foxp3 expression (Dikiy et al, 2021). CTLA-4 Ig treatment alone markedly reduced T-cell activation and also Foxp3 expression in consistent with mouse results. Co-administration of IL-2 restored Treg induction in comparable level with IL-2 alone group, however, CD25 expression in Foxp3^-^ non-Treg cells were significantly reduced in the presence of CTLA-4 Ig, indicating that effector T cell activation was still effectively restrained showing synergistic effects. Therefore, this combinatorial strategy may possess meaningful translational and clinical potential to treat MS.

Taken together, our finding shows that combining CTLA-4 Ig with a suboptimal dose of IL-2 robustly generates functional Treg populations while preserving effective inhibition of effector T cell activation. This combinatorial approach offers a more balanced immunomodulatory strategy and holds promise for broader therapeutic application in multiple sclerosis and other autoimmune diseases.

## Methods

**Table:** Reagents and tools table

Reagent/resource	Reference or source	Identifier or catalog number
CD4 + T Cell Isolation Kit	Miltenyi Biotec	Cat# 130-104-453
Recombinant human TGF-β	R&D Systems	Cat# 240-b
Recombinant murine IL-2	Peprotech	Cat# 212-12
Recombinant murine IL-6	R&D Systems	Cat# 406-ML
Recombinant human IL-2	Peprotech	Cat# 200-02
MOG_35-55_/CFA Emulsion PTX	HookeLab	Cat# EK-2110
[Ser^140^]-PLP_139-151_/CFA Emulsion PTX	HookeLab	Cat# EK2120
**Experimental models**		
SJL mice	Charles River	Yokohama, Japan
C57BL/6 mice	Daehan Biolink	Korea
2D2 TCR transgenic mice	Jackson Laboratory	Bar Harbor, ME, USA
CD4 Cre x STAT3fl/fl mice	Provided by Yeonseok Chung	Seoul National University, Korea
**Antibodies**		
Anti-mouse CD4 (RM4-5)	BioLegend	Cat# 100531
Anti-mouse CD25 (PC61)	BD Biosciences	Cat# 561112
Anti-mouse PD-1 (J43)	Invitrogen	Cat# 11-7311-81
Anti-mouse CD62L (MEL-14)	Invitrogen	Cat# 11-0621-81
Anti-mouse CD44 (IM7)	BioLegend	Cat# 103028
Anti-mouse IL-17A (eBio17B7)	Invitrogen	Cat# 11-7177-81
Anti-human CD4 (RPA-T4)	BioLegend	Cat# 300528
Anti-human CD45RA (HI100)	Invitrogen	Cat# 25-0458-42
Anti-human CD25 (BC96)	BioLegend	Cat# 302614
Anti-mouse Foxp3 (FJK-16s)	Invitrogen	Cat#17-5773-82
Anti-mouse CTLA-4 (UC10-4F10-11)	BD Biosciences	Cat# 561718
Anti-mouse pSTAT5 (47)	BD Biosciences	Cat# 560117
Anti-mouse pSmad2/3 (O72-670)	BD Biosciences	Cat# 562586
Anti-mouse pSTAT3 (pY705)	BD Biosciences	Cat# 560312
Anti-mouse pAKT (pS473)	BD Biosciences	Cat# 560343
Anti-human Foxp3 (259D)	BioLegend	Cat# 320208
Anti-human CTLA-4 (L3D10)	BioLegend	Cat# 349908
Phospho-SMAD2 (Ser465/467)/SMAD3 (Ser423/425) (D27F4) Rabbit Monoclonal Antibody	Cell Signaling Technology	Cat# 8828S
Phospho-Stat5 (Tyr694) Antibody	Cell Signaling Technology	Cat# 9351S
Phospho-Stat3 (Tyr705) Antibody	Cell Signaling Technology	Cat# 9131S
Phospho-Akt (Ser473) Antibody	Cell Signaling Technology	Cat# 9271S
IL-2 Monoclonal Antibody (JES6-1A12)	Invitrogen	Cat# 16-7022-81
Anti-Mo IFN gamma	Invitrogen	Cat# 16-7311-85
Anti-Mo IL-4	Invitrogen	Cat# 16-7041-85
**Chemicals, enzymes and other reagents**		
BD Phosflow Lyse/Fix Buffer	BD Biosciences	Cat# 558049
BD Phosflow Perm Buffer III	BD Biosciences	Cat# 558050
DNase I	Sigma-Aldrich	Cat# 10104159001
Collagenase D	Sigma-Aldrich	Cat# 11088866001
Percoll	GE Healthcare	Cat# 17544501
Foxp3/Transcription Factor Staining Buffer Set	eBioscience	Cat# 00-5523-00
Cell Stimulation Cocktail	eBioscience	Cat# 00-4975-03
Zombie Aqua Fixable Viability Kit	BioLegend	Cat# 423101
dNP2-ctCTLA-4	Anygen	
CTLA-4 Ig (Orencia)	Bristol Myers Squibb	Cat# NDC 0003-2187-10
MOG_35-55_ peptide	Anygen	
**Software**		
Flowjo software	BD Biosciences	
GraphPad Prism version 9.0	GraphPad Software	
**Other**		
FACS Canto II	BD Biosciences	
FACS Symphony A3	BD Biosciences	

### Mice

Eight-week-old female SJL mice were purchased from Charles River (Yokohama, Japan), 9 weeks old female C57BL/6 were purchased from Daehan Biolink (DBL, Korea) and 2D2 TCR transgenic mice were purchased from the Jackson Laboratory (Bar Harbor, ME, USA). 8 weeks old CD4 Cre x STAT3^fl/fl^ mice were provided by Yeonseok Chung (Seoul National University). Mice were housed and bred in a specific pathogen free animal facility at Hanyang University under controlled conditions with constant temperature (21 ± 1 ^o^C), humidity (50 ± 5%) and a 12 h light/dark cycle with regular chow and autoclaved water. All mouse experimental procedures used in this study carried out in accordance with the guidelines of the Institutional Animal Care and Use Committees of Hanyang University and all mice studies were randomized in a blinded manner.

### Antibodies

The following monoclonal antibodies were used for cell surface staining: anti-mouse CD4 (1:500; RM4-5, Biolegend), anti-mouse CD25 (1:500; PC61, BD Biosciences), anti-mouse PD-1 (1:500; J43, Invitrogen), anti-mouse CD62L (1:500; MEL-14, Invitrogen), anti-mouse CD44 (1:500; IM7, Biolegend), anti-IL-17A (1:200; eBio17B7, Invitrogen), anti-human CD4 (3 μl/sample; RPA-T4, Biolegend), anti-human CD45RA (3 μl/sample; HI100, Invitrogen), anti-human CD25 (3 μl/sample; BC96, Biolegend). The following antibodies were used for intracellular staining: anti-mouse Foxp3 (1:300; FJK-16s, Invitrogen), anti-mouse CTLA-4 (1:200; UC10-4F10-11, BD Biosciences), anti-mouse pSTAT5 (3 μl/sample; 47, BD Biosciences), anti-mouse pSmad2/3 (3 μl/sample; O72-670, BD Biosciences), anti-human Foxp3 (3 μl/sample; 259D, Biolegend), anti-human CTLA-4 (3 μl/sample; L3D10, Biolegend).

### In vitro T cell differentiation

Naive 2D2 CD4 T cells (CD4^+^ 2D2 Vβ11^+^ CD25^−^ CD62L^high^ CD44^low^) were isolated from splenocytes of 8–10 weeks old 2D2 mice by FACS sorting after CD4 T cell enrichment using CD4^+^ T Cell Isolation Kit (Miltenyi Biotec, Auburn, CA) according to manufacturer’s protocols. Antigen presenting cells were flushed out the remaining cells in the column after MACS, irradiated with 3500 rad gamma ray, and seeded with naive CD4 T cells at a 5:1 ratio. T cells were activated with 20 μg/ml (Th0, Th0+TGF-β, Th0+TGF-β + IL-2) or 40 μg/ml (Th17, Th17+IL-2, Th17+anti-IL-2) of MOG_35-55_ (Anygen) and differentiated with the following cytokine cocktails in a 96-well flat bottom plate for 3 days: no cytokine stimulation for Th0; 2 ng/ml of recombinant human TGF-β (240-Β-002, R&D Systems) for Th0+TGF-β; 2 ng/ml of recombinant human TGF-β, 50 U/ml of recombinant murine IL-2 (212-12, Peprotech) for Th0+TGF-β + IL-2; 2 ng/ml of recombinant human TGF-β, 30 ng/ml of murine IL-6 (406-ML, R&D system) for Th17; 2 ng/ml of recombinant human TGF-β, 30 ng/ml of murine IL-6, 50 U/ml of recombinant murine IL-2 for Th17+IL-2; 2 ng/ml of recombinant human TGF-β, 30 ng/ml of murine IL-6, 5 μg/ml of anti-IL-2 (JES6-1A12, Invitrogen) for Th17+anti-IL-2. Cells were treated with 5 μM of dNP2-ctCTLA-4 (Anygen) or 0.5 μM of CTLA-4 Ig.

### Flowcytometry

Cells were analyzed by flow cytometry. Cells were restimulated with Cell Stimulation Cocktail (00-4975-03; eBioscience) to determine intracellular cytokine levels for 4 h at 37 °C. Cells were stained to exclude dead cells using Zombie Aqua Fixable Viability Kit (423101, BioLegend) at room temperature for 10 min. After washing with PBS, the surface proteins were stained with specific monoclonal antibodies for 30 min at 4 °C. After washing with PBS, cells were fixed and permeabilized using Foxp3/Transcription Factor Staining Buffer Set (00-5523-00, eBioscience) for 30 min at room temperature. At last, intracellular target proteins were stained with monoclonal antibodies for 30 min at room temperature. FACS Canto II or FACS Symphony A3 was used to acquire data and FlowJo software version 10.8.0 was used to analyze the data.

### Analysis of published RNA-sequencing dataset

Publicly available RNA-sequencing data from rheumatoid arthritis patients (accession number: 8250013) were obtained from Zenodo (Iwasaki et al, 2024). The dataset was originally published in Iwasaki et al (2024), which has been cited accordingly. Processed expression data (CPM values) provided in the dataset were used for analysis. Z-scores for selected target genes were calculated based on CPM values and visualized using GraphPad Prism version 9.0.

### Intracellular signaling molecule staining

Splenocyte isolated from 10 weeks old female C57BL/6 mice were isolated activated with soluble anti-CD3 (2 μg/ml, 553057, BD Pharmingen) antibodies and differentiated with the following cytokine cocktails in a 96-well plate for 3 days: 2 ng/ml of TGF-β (240-Β-002, R&D System) for Th0+TGF-β; 2 ng/ml of TGF-β and 50 U/ml of IL-2 (212-12, Peprotech) for Th0+TGF-β + IL-2. Additionally, cells were treated with 0.5 μM of CTLA-4 Ig. After 3 days, cells were stained to exclude dead cells using Zombie Aqua Fixable Viability Kit (423101, BioLegend) at room temperature for 10 min. After washing with PBS, the surface proteins were stained with specific monoclonal antibodies for 30 min at 4 °C. After washing with PBS, cells were fixed with pre-warmed BD Phosflow Lyse/Fix Buffer (558049; BD Biosciences) at 37 °C for 10 min. After washed with HBSS, cells were permeabilized with pre-chilled BD Phosflow Perm Buffer III (558050, BD Biosciences) at 4 °C for 30 min. At last, intracellular signaling molecules and protein were stained with anti-mouse phosphor-STAT5 (612699, BD Biosciences), anti-mouse phosphor-Smad2/3 (562586, BD Biosciences) and anti-mouse Foxp3. Cells were analyzed with FACS Canto II or Symphony A3 and FlowJo software version 10.8.0.

### Immunoblot analysis

After T cell differentiation in each condition, cells were harvested and washed with PBS. Cells were lysed by RIPA (Cell Signaling, Beverly, MA) with 10^−3 ^M of PMSF and 10^−3 ^M of NaF for 30 min on ice. Immunoblotting was performed on PVDF membranes (Bio-Rad) using the following primary antibodies: phospho-Smad2/3 C-term (1:1000; Cell Signaling Technology, D27F4), phospho-STAT3 (Y705) (1:1000; Cell Signaling Technology), phospho-STAT5 (1:1000; Cell Signaling Technology), phospho-AKT (1:1000; Cell Signaling Technology) and β-actin mouse mAb (1:1000; Santa Cruz).

### Active EAE induction

Nine-week-old female C57BL/6 mice were purchased from Daehan Biolink (Eumseong, Korea). The protocol described here was approved by the Animal Experimentation Ethics Committee of Hanyang University. EAE was induced by subcutaneous immunization using the MOG_35-55_/CFA Emulsion PTX kit (Hooke Labs, Lawrence, MA) according to the manufacturer’s protocol. Mice were anesthetized with isoflurane and 100 μg of MOG_35-55_/CFA was subcutaneously injected bilaterally, followed by pertussis toxin (PTX) intraperitoneal injection 2 and 24 h later. Mice were randomized to different groups after MOG_35-55_/CFA immunization. EAE score and body weight were assessed daily using the following scoring system: 0, no obvious signs of disease; 0.5, partially limp tail; 1, completely limp tail; 1.5, limb tail and waddling gait; 2, paralysis of one hind limb; 2.5, paralysis of one hind limb and partial paralysis of the other hind limb; 3, paralysis of both hind limbs; 3.5, ascending paralysis; 4, paralysis of trunk; 4.5, moribund; 5, dead. Although treatment groups were randomized at the time of allocation, clinical assessment was not performed under blinded conditions. 20 ng or 50 ng of IL-2, 200 μg of CTLA-4 Ig (Orencia, BMS) was injected intraperitoneally daily from day 7. Mice were euthanized at the end of the experiments; isolated spinal cord was digested with 1 mg/ml of DNase 1 (10 104 159 001, Sigma-Aldrich) and 1 mg/ml of Collagenase D (11 088 866 001, Sigma-Aldrich) at 37 °C and incubated at 80 RPM on shaker for 40 min. After enzyme digestion, 0.5 mM ethylenediaminetetraacetic acid (EDTA) was added and lymphocytes were isolated by Percoll (GE Healthcare, Little Chalfont, UK) density-gradient centrifugation. Isolated spinal cord-infiltrated lymphocytes and lymphocytes from iLN were stained with anti-mouse CD4, anti-mouse CD25, anti-mouse CD62L, and anti-mouse CD44. Cells were then fixed, permeabilized using Foxp3/Transcription Factor Staining Buffer Set (00-5523-00, eBioscience), and stained with anti-mouse Foxp3 and anti-IL-17A. Cells were analyzed using FACS Canto and Symphony A3 and Flowjo software version 10.8.0.

### Relapsing-remitting EAE mouse model

Eight-week-old female SJL mice were purchased from Charles River. EAE was induced by subcutaneous immunization with an PLP_131-151_/CFA Emulsion PTX kit (Hooke Labs, Lawrence, MA) according to the manufacturer’s protocol. Mice were anesthetized with isoflurane and PLP_131-151_/CFA was subcutaneously injected at two sites on the upper back and lower back, followed by pertussis toxin (PTX) intraperitoneal injection immediately. Clinical scoring was performed as described above. 100 μg of dNP2-ctCTLA-4 (Anygen) and 100 μg of CTLA-4 Ig (Orencia, BMS) was injected intraperitoneally daily from day 7 to day 20 during acute phase. After treatment, clinical disease score chased to day 42.

### In vitro human Treg induction

PBMC from MS patients used in study were obtained from total 10 donors at Seoul National University College of Medicine (10 RRMS patient donors: 7 females and 3 males). Informed consent was obtained from all participants. The study was approved by the Institutional Review Board of Seoul National University Hospital (IRB number: H-1902-083-1010) and conducted in accordance with the principles of the Declaration of Helsinki and the Department of Health and Human Services Belmont Report. Demographic information of MS patients is described in Appendix Table S1. Human PBMCs were stimulated with 5 μg/ml of anti-CD3 (OKT3, 16-0037-85, ThermoFisher) in presence of 2 ng/ml of TGF-β (240-b, R&D Systems) and 50 U/ml of rhIL-2 (200-02, Peprotech) for 3 days. Cells were treated with 0.5 μM of CTLA-4 Ig. On day 3, cells were stained with Zombie Aqua Fixable Viability Kit (423101, BioLegend) for 10 min at room temperature, then stained with anti-human CD4, anti-human CD45RA, anti-human CD25 for 30 min at 4 °C. After fixation and permeabilization by Foxp3/Transcription Factor Staining Set (eBiosciences) for 30 min at room temperature, intracellular staining was performed using anti-human Foxp3 and anti-human CTLA-4. Stained cells were analyzed using a FACS Canto II.

### Intact mice CTLA-4 Ig injection

Ten-week-old female C57BL/6 mice were intraperitoneally injected 400 μg of CTLA-4 Ig once every two days from day 0 to day 6 and analyzed at day 7. On day 7, mice were sacrificed and the phenotypic analysis of lymphocyte from spleen using flow cytometry.

### Ethics statement

The animal study was approved by Institutional Animal Care and Use Committees of Hanyang University (2023-0308 A, 2024-0227 A). The study was conducted in accordance with the local legislation and institutional requirements. PBMCs of human RRMS patients were collected and provided by Seoul National University Hospital. This study was approved by the Institutional Review Board of Seoul National University Hospital (IRB number: H-1902-083-1010). The use of human samples from MS patients in this study was deemed exempt from IRB reviewed by the Hanyang University Institutional Review Board, as it falls under the category of research using de-identified biological specimens (HYUIRB-202502-006).

### Statistical analysis

All data were analyzed using Mann–Whitney test or two-way ANOVA in GraphPad Prism version 9.0 (Graphpad Software, San Diego, CA). No data or outliers were excluded. Data presented as mean ± SD or ± SEM in all cases, significance was defined as *P* ≤ 0.05. Sample size and statistical analysis information is provided in with figure legend. Animals were randomly assigned to experimental groups where applicable. Investigators were not blinded to group allocation during experiments and outcome assessment. All experiments were performed with at least three independent biological replicates unless otherwise stated.

### Graphics

Schematics and graphical illustrations were created with BioRender.com (Figs. 1A,H, 2A, 3A, 4L, 5A and  EV1A; Appendix Fig. S1A; Synopsis).

## Supplementary information

Appendix

Peer Review File

Dataset EV1

Source data Fig. 1

Source data Fig. 2

Source data Fig. 3

Source data Fig. 4

Source data Fig. 5

Expanded View Figures

## Data Availability

This study includes no data deposited in external repositories. The source data of this paper are collected in the following database record: biostudies:S-SCDT-10_1038-S44321-026-00431-7.

## References

[CR1] Aldridge J, Andersson K, Gjertsson I, Hultgård Ekwall A-K, Hallström M, van Vollenhoven R, Lundell A-C, Rudin A (2022) Blood PD-1+ TFh and CTLA-4+ CD4+ T cells predict remission after CTLA-4Ig treatment in early rheumatoid arthritis. Rheumatology 61:1233–1242 34009274 10.1093/rheumatology/keab454 PMC8889294

[CR2] Azuma M, Ito D, Yagita H, Okumura K, Phillips JH, Lanier LL, Somoza C (1993) B70 antigen is a second ligand for CTLA-4 and CD28. Nature 366:76–79 7694153 10.1038/366076a0

[CR3] Baroja ML, Vijayakrishnan L, Bettelli E, Darlington PJ, Chau TA, Ling V, Collins M, Carreno BM, Madrenas J, Kuchroo VK (2002) Inhibition of CTLA-4 function by the regulatory subunit of serine/threonine phosphatase 2A. J Immunol 168:5070–5078 11994459 10.4049/jimmunol.168.10.5070

[CR4] Bradshaw JD, Lu P, Leytze G, Rodgers J, Schieven GL, Bennett KL, Linsley PS, Kurtz SE (1997) Interaction of the cytoplasmic tail of CTLA-4 (CD152) with a clathrin-associated protein is negatively regulated by tyrosine phosphorylation. Biochemistry 36:15975–15982 9398332 10.1021/bi971762i

[CR5] Cavallo S (2020) Immune-mediated genesis of multiple sclerosis. J Transl Autoimmun 3:100039 32743522 10.1016/j.jtauto.2020.100039 PMC7388381

[CR6] Cerosaletti K, Schneider A, Schwedhelm K, Frank I, Tatum M, Wei S, Whalen E, Greenbaum C, Kita M, Buckner J (2013) Multiple autoimmune-associated variants confer decreased IL-2R signaling in CD4+ CD25hi T cells of type 1 diabetic and multiple sclerosis patients. PLoS ONE 8:e83811 24376757 10.1371/journal.pone.0083811 PMC3871703

[CR7] Chikuma S, Abbas AK, Bluestone JA (2005) B7-independent inhibition of T cells by CTLA-4. J Immunol 175:177–181 15972645 10.4049/jimmunol.175.1.177

[CR8] Choi J-M, Ahn M-H, Chae W-J, Jung Y-G, Park J-C, Song H-M, Kim Y-E, Shin J-A, Park C-S, Park J-W (2006) Intranasal delivery of the cytoplasmic domain of CTLA-4 using a novel protein transduction domain prevents allergic inflammation. Nat Med 12:574–579 16604087 10.1038/nm1385

[CR9] Choi J-M, Kim S-H, Shin J-H, Gibson T, Yoon B-S, Lee D-H, Lee S-K, Bothwell AL, Lim J-S, Lee S-K (2008) Transduction of the cytoplasmic domain of CTLA-4 inhibits TcR-specific activation signals and prevents collagen-induced arthritis. Proc Natl Acad Sci USA 105:19875–19880 19066215 10.1073/pnas.0805198105 PMC2604944

[CR10] Confavreux C, Compston A (2009) The natural history of multiple sclerosis. McAlpine’s Mult Scler 183–272. 10.1016/B978-0-443-07271-0.50006-9

[CR11] Dikiy S, Li J, Bai L, Jiang M, Janke L, Zong X, Hao X, Hoyos B, Wang Z-M, Xu B (2021) A distal Foxp3 enhancer enables interleukin-2 dependent thymic Treg cell lineage commitment for robust immune tolerance. Immunity 54:931–946 33838102 10.1016/j.immuni.2021.03.020 PMC8317508

[CR12] Fuentes-Rumí L, Hernández-Clares R, Carreón-Guarnizo E, Valero-López G, Iniesta-Martinez F, Cabrera-Maqueda JM, León-Hernández A, Zamarro-Parra J, Morales-Ortiz A, Meca-Lallana JE (2020) Prevention of rebound effect after natalizumab withdrawal in multiple sclerosis. study of two high-dose methylprednisolone schedules. Mult Scler Relat Disord 44:102311 32593958 10.1016/j.msard.2020.102311

[CR13] Fung MM, Rohwer F, McGuire KL (2003) IL-2 activation of a PI3K-dependent STAT3 serine phosphorylation pathway in primary human T cells. Cell Signal 15:625–636 12681450 10.1016/s0898-6568(03)00003-2

[CR14] Genovese MC, Becker J-C, Schiff M, Luggen M, Sherrer Y, Kremer J, Birbara C, Box J, Natarajan K, Nuamah I (2005) Abatacept for rheumatoid arthritis refractory to tumor necrosis factor α inhibition. N Engl J Med 353:1114–1123 16162882 10.1056/NEJMoa050524

[CR15] Glatigny S, Höllbacher B, Motley SJ, Tan C, Hundhausen C, Buckner JH, Smilek D, Khoury SJ, Ding L, Qin T (2019) Abatacept targets T follicular helper and regulatory T cells, disrupting molecular pathways that regulate their proliferation and maintenance. J Immunol 202:1373–1382 30683697 10.4049/jimmunol.1801425 PMC6481683

[CR16] He J, Zhang X, Wei Y, Sun X, Chen Y, Deng J, Jin Y, Gan Y, Hu X, Jia R (2016) Low-dose interleukin-2 treatment selectively modulates CD4+ T cell subsets in patients with systemic lupus erythematosus. Nat Med 22:991–993 27500725 10.1038/nm.4148

[CR17] Iwasaki T, Watanabe R, Ito H, Fujii T, Ohmura K, Yoshitomi H, Murata K, Murakami K, Onishi A, Tanaka M (2024) Monocyte-derived transcriptomes explain the ineffectiveness of abatacept in rheumatoid arthritis. Arthritis Res Ther 26:1 38167328 10.1186/s13075-023-03236-y PMC10759752

[CR18] Jago, Yates C, Olsen J, Saraiva, Câmara N, Lechler R, Lombardi G (2004) Differential expression of CTLA-4 among T cell subsets. Clin Exp Immunol 136:463–471 15147348 10.1111/j.1365-2249.2004.02478.x PMC1809051

[CR19] Jamshidian A, Shaygannejad V, Pourazar A, Zarkesh-Esfahani S-H, Gharagozloo M (2013) Biased Treg/Th17 balance away from regulatory toward inflammatory phenotype in relapsed multiple sclerosis and its correlation with severity of symptoms. J Neuroimmunol 262:106–112 23845464 10.1016/j.jneuroim.2013.06.007

[CR20] Jeffery DR, Rammohan KW, Hawker K, Fox E (2016) Fingolimod: a review of its mode of action in the context of its efficacy and safety profile in relapsing forms of multiple sclerosis. Expert Rev Neurotherapeutics 16:31–44 10.1586/14737175.2016.1123094 26587577

[CR21] Johnston JA, Bacon CM, Finbloom DS, Rees RC, Kaplan D, Shibuya K, Ortaldo JR, Gupta S, Chen YQ, Giri JD (1995) Tyrosine phosphorylation and activation of STAT5, STAT3, and Janus kinases by interleukins 2 and 15. Proc Natl Acad Sci 92:8705–8709 7568001 10.1073/pnas.92.19.8705 PMC41035

[CR22] Josefowicz SZ, Lu L-F, Rudensky AY (2012) Regulatory T cells: mechanisms of differentiation and function. Annu Rev Immunol 30:531–564 22224781 10.1146/annurev.immunol.25.022106.141623 PMC6066374

[CR23] Karnitz LM, Burns LA, Sutor SL, Blenis J, Abraham RT (1995) Interleukin-2 triggers a novel phosphatidylinositol 3-kinase-dependent MEK activation pathway. Mol Cell Biol 15:3049–3057 7760801 10.1128/mcb.15.6.3049 PMC230536

[CR24] Kawabata M, Heldin N, Heldin C, ten Dijke P (1997) Identification of Smad7, a TGFbeta-inducible antagonist of TGF-beta signalling. Nature 389:631–635 9335507 10.1038/39369

[CR25] Khoury SJ, Rochon J, Ding L, Byron M, Ryker K, Tosta P, Gao W, Freedman MS, Arnold DL, Sayre PH et al (2017) ACCLAIM: a randomized trial of abatacept (CTLA4-Ig) for relapsing-remitting multiple sclerosis. Mult Scler 23:686–695 27481207 10.1177/1352458516662727 PMC5288398

[CR26] Kim D, Lee N, Ha S-J (2026) Regulatory T cell heterogeneity in the steady state and tumor. Immune Netw 26:e10 41800022 10.4110/in.2026.26.e10 PMC12962836

[CR27] Kim G-R, Choi J-M (2022) Current understanding of cytotoxic T lymphocyte antigen-4 (CTLA-4) signaling in T-cell biology and disease therapy. Mol Cells 45:513–521 35950451 10.14348/molcells.2022.2056 PMC9385567

[CR28] Kim G-R, Kim W-J, Lim S, Lee H-G, Koo J-H, Nam K-H, Kim S-M, Park S-D, Choi J-M (2021a) In vivo induction of regulatory T cells via CTLA-4 signaling peptide to control autoimmune encephalomyelitis and prevent disease relapse. Adv Sci 8:2004973 10.1002/advs.202004973 PMC8292875 34306974

[CR29] Kim G-R, Nam K-H, Choi J-M (2024) Belatacept and regulatory T cells in transplantation: synergistic strategies for immune tolerance and graft survival. Clin Transplant Res 38:326–340 39690903 10.4285/ctr.24.0057 PMC11732762

[CR30] Kim W-J, Kim G-R, Cho H-J, Choi J-M (2021b) The cysteine-containing cell-penetrating peptide AP enables efficient macromolecule delivery to T cells and controls autoimmune encephalomyelitis. Pharmaceutics 13:1134 34452095 10.3390/pharmaceutics13081134 PMC8401785

[CR31] Kong K-F, Fu G, Zhang Y, Yokosuka T, Casas J, Canonigo-Balancio AJ, Becart S, Kim G, Yates IIIJR, Kronenberg M (2014) Protein kinase C-η controls CTLA-4-mediated regulatory T cell function. Nat Immunol 15:465–472 24705298 10.1038/ni.2866 PMC4040250

[CR32] Koreth J, Matsuoka K-I, Kim HT, McDonough SM, Bindra B, Alyea IIIEP, Armand P, Cutler C, Ho VT, Treister NS (2011) Interleukin-2 and regulatory T cells in graft-versus-host disease. N Engl J Med 365:2055–2066 22129252 10.1056/NEJMoa1108188 PMC3727432

[CR33] Kougkas N, Kruger-Krasagakis S, Papadaki E, Mastorodemos V (2022) Successful treatment of highly active multiple sclerosis and psoriasis exacerbation with natalizumab and secukinumab combination. a case report and literature review. Neuroimmunol Rep 2:100054

[CR34] Krummel MF, Allison JP (1995) CD28 and CTLA-4 have opposing effects on the response of T cells to stimulation. J Exp Med 182:459–465 7543139 10.1084/jem.182.2.459 PMC2192127

[CR35] Lee W-S, Nam K-H, Kim JH, Kim W-J, Kim JE, Shin E-C, Kim G-R, Choi J-M (2023) Alleviating psoriatic skin inflammation through augmentation of Treg cells via CTLA-4 signaling peptide. Front Immunol 14:1233514 37818377 10.3389/fimmu.2023.1233514 PMC10560854

[CR36] Lim S, Ho Sohn J, Koo J-H, Park J-W, Choi J-M (2017) dNP2-ctCTLA-4 inhibits German cockroach extract-induced allergic airway inflammation and hyper-responsiveness via inhibition of Th2 responses. Exp Mol Med 49:e362 28775364 10.1038/emm.2017.107 PMC5579505

[CR37] Lim S, Kim W-J, Kim Y-H, Lee S, Koo J-H, Lee J-A, Yoon H, Kim D-H, Park H-J, Kim H-M (2015) dNP2 is a blood–brain barrier-permeable peptide enabling ctCTLA-4 protein delivery to ameliorate experimental autoimmune encephalomyelitis. Nat Commun 6:8244 26372309 10.1038/ncomms9244 PMC4579786

[CR38] Linsley PS, Brady W, Urnes M, Grosmaire LS, Damle NK, Ledbetter JA (1991) CTLA-4 is a second receptor for the B cell activation antigen B7. J Exp Med 174:561–569 1714933 10.1084/jem.174.3.561 PMC2118936

[CR39] Lo B, Zhang K, Lu W, Zheng L, Zhang Q, Kanellopoulou C, Zhang Y, Liu Z, Fritz JM, Marsh R (2015) Patients with LRBA deficiency show CTLA4 loss and immune dysregulation responsive to abatacept therapy. Science 349:436–440 26206937 10.1126/science.aaa1663

[CR40] Lorenz U (2009) SHP-1 and SHP-2 in T cells: two phosphatases functioning at many levels. Immunol Rev 228:342–359 19290938 10.1111/j.1600-065X.2008.00760.x PMC2669678

[CR41] Luwor R, Baradaran B, Taylor L, Iaria J, Nheu T, Amiry N, Hovens C, Wang B, Kaye A, Zhu H (2013) Targeting Stat3 and Smad7 to restore TGF-β cytostatic regulation of tumor cells in vitro and in vivo. Oncogene 32:2433–2441 22751114 10.1038/onc.2012.260 PMC3655378

[CR42] Malpas CB, Roos I, Sharmin S, Buzzard K, Skibina O, Butzkueven H, Kappos L, Patti F, Alroughani R, Horakova D (2022) Multiple sclerosis relapses following cessation of fingolimod. Clin Drug Investig 42:355–364 35303292 10.1007/s40261-022-01129-7 PMC8989797

[CR43] McCormack PL (2013) Natalizumab: a review of its use in the management of relapsing-remitting multiple sclerosis. Drugs 73:1463–1481 23912625 10.1007/s40265-013-0102-7

[CR44] Mease PJ, Gottlieb AB, van der Heijde D, FitzGerald O, Johnsen A, Nys M, Banerjee S, Gladman DD (2017) Efficacy and safety of abatacept, a T-cell modulator, in a randomised, double-blind, placebo-controlled, phase III study in psoriatic arthritis. Ann Rheum Dis 76:1550–1558 28473423 10.1136/annrheumdis-2016-210724 PMC5561378

[CR45] Mikami N, Kawakami R, Chen KY, Sugimoto A, Ohkura N, Sakaguchi S (2020) Epigenetic conversion of conventional T cells into regulatory T cells by CD28 signal deprivation. Proc Natl Acad Sci USA 117:12258–12268 32414925 10.1073/pnas.1922600117 PMC7275710

[CR46] Miyara M, Yoshioka Y, Kitoh A, Shima T, Wing K, Niwa A, Parizot C, Taflin C, Heike T, Valeyre D (2009) Functional delineation and differentiation dynamics of human CD4+ T cells expressing the FoxP3 transcription factor. Immunity 30:899–911 19464196 10.1016/j.immuni.2009.03.019

[CR47] Mulero P, Midaglia L, Montalban X (2018) Ocrelizumab: a new milestone in multiple sclerosis therapy. Ther Adv Neurol Disord 11:1756286418773025 29774057 10.1177/1756286418773025 PMC5952271

[CR48] Orban T, Beam CA, Xu P, Moore K, Jiang Q, Deng J, Muller S, Gottlieb P, Spain L, Peakman M (2014) Reduction in CD4 central memory T-cell subset in costimulation modulator abatacept-treated patients with recent-onset type 1 diabetes is associated with slower C-peptide decline. Diabetes 63:3449–3457 24834977 10.2337/db14-0047 PMC4171657

[CR49] Parulekar AD, Boomer JS, Patterson BM, Yin-Declue H, Deppong CM, Wilson BS, Jarjour NN, Castro M, Green JM (2013) A randomized controlled trial to evaluate inhibition of T-cell costimulation in allergen-induced airway inflammation. Am J Respir Crit Care Med 187:494–501 23292882 10.1164/rccm.201207-1205OC PMC5448510

[CR50] Peelen E, Damoiseaux J, Smolders J, Knippenberg S, Menheere P, Tervaert JWC, Hupperts R, Thewissen M (2011) Th17 expansion in MS patients is counterbalanced by an expanded CD39+ regulatory T cell population during remission but not during relapse. J Neuroimmunol 240:97–103 22035960 10.1016/j.jneuroim.2011.09.013

[CR51] Pieper J, Herrath J, Raghavan S, Muhammad K, Vollenhoven RV, Malmström V (2013) CTLA4-Ig (abatacept) therapy modulates T cell effector functions in autoantibody-positive rheumatoid arthritis patients. BMC Immunol 14:34 23915385 10.1186/1471-2172-14-34 PMC3750242

[CR52] Qureshi OS, Zheng Y, Nakamura K, Attridge K, Manzotti C, Schmidt EM, Baker J, Jeffery LE, Kaur S, Briggs Z (2011) Trans-endocytosis of CD80 and CD86: a molecular basis for the cell-extrinsic function of CTLA-4. Science 332:600–603 21474713 10.1126/science.1202947 PMC3198051

[CR53] Raeber ME, Sahin D, Karakus U, Boyman O (2023) A systematic review of interleukin-2-based immunotherapies in clinical trials for cancer and autoimmune diseases. EBioMedicine 90:104539 10.1016/j.ebiom.2023.104539 PMC10111960 37004361

[CR54] Read S, Malmström V, Powrie F (2000) Cytotoxic T lymphocyte–associated antigen 4 plays an essential role in the function of CD25+ CD4+ regulatory cells that control intestinal inflammation. J Exp Med 192:295–302 10899916 10.1084/jem.192.2.295 PMC2193261

[CR55] Sakaguchi S, Sakaguchi N, Asano M, Itoh M, Toda M (1995) Immunologic self-tolerance maintained by activated T cells expressing IL-2 receptor alpha-chains (CD25). Breakdown of a single mechanism of self-tolerance causes various autoimmune diseases. J Immunol 155:1151–1164 7636184

[CR56] Salomon B, Lenschow DJ, Rhee L, Ashourian N, Singh B, Sharpe A, Bluestone JA (2000) B7/CD28 costimulation is essential for the homeostasis of the CD4+ CD25+ immunoregulatory T cells that control autoimmune diabetes. Immunity 12:431–440 10795741 10.1016/s1074-7613(00)80195-8

[CR57] Sandborn WJ, Colombel JF, Sands BE, Rutgeerts P, Targan SR, Panaccione R, Bressler B, Geboes K, Schreiber S, Aranda R (2012) Abatacept for Crohn’s disease and ulcerative colitis. Gastroenterology 143:62–69 22504093 10.1053/j.gastro.2012.04.010

[CR58] Schneider H, Martin M, Agarraberes FA, Yin L, Rapoport I, Kirchhausen T, Rudd CE (1999) Cytolytic T lymphocyte-associated antigen-4 and the TCRζ/CD3 complex, but not CD28, interact with clathrin adaptor complexes AP-1 and AP-2. J Immunol 163:1868–1879 10438921

[CR59] Schneider H, Rudd CE (2000) Tyrosine phosphatase SHP-2 binding to CTLA-4: absence of direct YVKM/YFIP motif recognition. Biochem Biophys Res Commun 269:279–283 10694513 10.1006/bbrc.2000.2234

[CR60] Shouse AN, LaPorte KM, Malek TR (2024) Interleukin-2 signaling in the regulation of T cell biology in autoimmunity and cancer. Immunity 57:414–428 38479359 10.1016/j.immuni.2024.02.001 PMC11126276

[CR61] Silva PDM, Bier J, Paiatto LN, Galdino Albuquerque C, Lopes Souza C, Fernandes LGR, Tamashiro WMDSC, Simioni PU (2015) Tolerogenic dendritic cells on transplantation: immunotherapy based on second signal blockage. J Immunol Res 2015:856707 26543876 10.1155/2015/856707 PMC4620289

[CR62] Takahashi T, Tagami T, Yamazaki S, Uede T, Shimizu J, Sakaguchi N, Mak TW, Sakaguchi S (2000) Immunologic self-tolerance maintained by CD25+ CD4+ regulatory T cells constitutively expressing cytotoxic T lymphocyte–associated antigen 4. J Exp Med 192:303–310 10899917 10.1084/jem.192.2.303 PMC2193248

[CR63] Tang Q, Henriksen KJ, Boden EK, Tooley AJ, Ye J, Subudhi SK, Zheng XX, Strom TB, Bluestone JA (2003) Cutting edge: CD28 controls peripheral homeostasis of CD4+ CD25+ regulatory T cells. J Immunol 171:3348–3352 14500627 10.4049/jimmunol.171.7.3348

[CR64] Tarantelli C, Argnani L, Zinzani PL, Bertoni F (2021) PI3Kδ inhibitors as immunomodulatory agents for the treatment of lymphoma patients. Cancers 13:5535 34771694 10.3390/cancers13215535 PMC8582887

[CR65] Teft WA, Chau TA, Madrenas J (2009) Structure-function analysis of the CTLA-4 interaction with PP2A. BMC Immunol 10:23 19405949 10.1186/1471-2172-10-23 PMC2683795

[CR66] Tselis A, Khan O, Lisak RP (2007) Glatiramer acetate in the treatment of multiple sclerosis. Neuropsychiatr Dis Treat 3:259–267 19300558 10.2147/nedt.2007.3.2.259 PMC2654627

[CR67] Vijayakrishnan L, Slavik JM, Illés Z, Greenwald RJ, Rainbow D, Greve B, Peterson LB, Hafler DA, Freeman GJ, Sharpe AH (2004) An autoimmune disease-associated CTLA-4 splice variant lacking the B7 binding domain signals negatively in T cells. Immunity 20:563–575 15142525 10.1016/s1074-7613(04)00110-4

[CR68] Vincenti F (2008) Costimulation blockade in autoimmunity and transplantation. J Allergy Clin Immunol 121:299–306 18269922 10.1016/j.jaci.2008.01.002

[CR69] Vincenti F, Rostaing L, Grinyo J, Rice K, Steinberg S, Gaite L, Moal M-C, Mondragon-Ramirez GA, Kothari J, Polinsky MS (2016) Belatacept and long-term outcomes in kidney transplantation. N Engl J Med 374:333–343 26816011 10.1056/NEJMoa1506027

[CR70] Vogel I, Kasran A, Cremer J, Kim YJ, Boon L, Van Gool SW, Ceuppens JL (2015) CD28/CTLA-4/B7 costimulatory pathway blockade affects regulatory T-cell function in autoimmunity. Eur J Immunol 45:1832–1841 25727069 10.1002/eji.201445190

[CR71] Wang CJ, Petersone L, Edner NM, Heuts F, Ovcinnikovs V, Ntavli E, Kogimtzis A, Fabri A, Elfaki Y, Houghton LP et al (2022) Costimulation blockade in combination with IL-2 permits regulatory T cell sparing immunomodulation that inhibits autoimmunity. Nat Commun 13:6757 36347877 10.1038/s41467-022-34477-1 PMC9643453

[CR72] Wang G, Yu Y, Sun C, Liu T, Liang T, Zhan L, Lin X, Feng X-H (2016) STAT3 selectively interacts with Smad3 to antagonize TGF-β. Oncogene 35:4388–4398 26616859 10.1038/onc.2015.446 PMC4885808

[CR73] Watkins B, Qayed M, McCracken C, Bratrude B, Betz K, Suessmuth Y, Yu A, Sinclair S, Furlan S, Bosinger S (2021) Phase II trial of costimulation blockade with abatacept for prevention of acute GVHD. J Clin Oncol 39:1865–1877 33449816 10.1200/JCO.20.01086 PMC8260909

[CR74] Webster KE, Walters S, Kohler RE, Mrkvan T, Boyman O, Surh CD, Grey ST, Sprent J (2009) In vivo expansion of T reg cells with IL-2–mAb complexes: induction of resistance to EAE and long-term acceptance of islet allografts without immunosuppression. J Exp Med 206:751–760 19332874 10.1084/jem.20082824 PMC2715127

[CR75] Zappasodi R, Serganova I, Cohen IJ, Maeda M, Shindo M, Senbabaoglu Y, Watson MJ, Leftin A, Maniyar R, Verma S (2021) CTLA-4 blockade drives loss of Treg stability in glycolysis-low tumours. Nature 591:652–658 33588426 10.1038/s41586-021-03326-4 PMC8057670

[CR76] Zhang R, Huynh A, Whitcher G, Chang J, Maltzman JS, Turka LA (2013) An obligate cell-intrinsic function for CD28 in Tregs. J Clin Investig 123:580–593 23281398 10.1172/JCI65013 PMC3561819

[CR77] Zhang W, Liu X, Zhu Y, Liu X, Gu Y, Dai X, Li B (2021) Transcriptional and posttranslational regulation of Th17/Treg balance in health and disease. Eur J Immunol 51:2137–2150 34322865 10.1002/eji.202048794

