# Supplementary material for: Costimulation loss enhances IL-2-driven Treg generation by PI3K-STAT3 inhibition in CNS autoimmunity
Source: EMBO Mol Med. 2026 May 5;18(6):2272–92. doi: 10.1038/s44321-026-00431-7 (PMC13269791; doi:10.1038/s44321-026-00431-7)
Supplement: Supplementary file 1 — Appendix [file 44321_2026_431_MOESM1_ESM.pdf]

Appendix

Costimulation loss enhances IL-2-driven Treg generation by PI3K-STAT3 inhibition in CNS autoimmunity

Kyung-Ho Nam[1†], Gil-Ran Kim[1,2†], Yu-Rim Kim[1], Young Nam Kwon[3,4], Sung-Min Kim[5], Je-Min Choi[1,2,6,7,*]

[1]Department of Life Science, College of Natural Sciences, Hanyang University, Seoul, Republic of Korea

[2]Research Institute for Natural Sciences, Hanyang University, Seoul, Republic of Korea

[3]Department of Neurology, Severance Hospital, Yonsei University College of Medicine, Seoul, Republic of Korea

[4]Biomedical Research Institute, Department of Neurology, Seoul National University Hospital, Seoul, Republic of Korea

[5]Department of Neurology, Seoul National University, College of Medicine, Seoul, Republic of Korea

[6]Hanyang Institute of Bioscience and Biotechnology, Hanyang University, Seoul, Republic of Korea

[7]Research Institute for Convergence of Basic Sciences, Hanyang University, Seoul, Republic of Korea

* Correspondence: Je-Min Choi
Email: jeminchoi@hanyang.ac.kr

Table of Contents

	Page
**Appendix Figure S1.** eTreg cells exhibit higher suppressive function compared to Treg cells in vitro.	3
**Appendix Figure S2.** Costimulation blockade by CTLA-4 Ig reduces thymic Treg cells in steady-state mice.	4
**Appendix Figure S3.** Gating strategies and effects of CTLA-4 Ig on $CD4^+$ T cells in in vitro co-culture system.	5
**Appendix Figure S4.** CTLA-4 Ig induces Treg differentiation under minimal TGF-β conditions without dose dependency.	6
**Appendix Figure S5.** dNP2-ctCTLA-4 enhances TGF-β-dependent, but not IL-2-driven, Foxp3 induction.	7
**Appendix Figure S6.** IL-2 promotes selective Treg induction under low-costimulation conditions.	8
**Appendix Figure S7.** FACS dot plots of signaling molecules in each group of EAE experiment.	9
**Appendix Figure S8.** CTLA-4 Ig consistently reduces pSTAT3 levels when normalized to either STAT3 or β-actin.	10
**Appendix Figure S9.** STAT3 phosphorylation by IL-2 requires T cell receptor stimulation.	11
**Appendix Figure S10.** Classification of human Treg subpopulation in unstimulated and Treg-induced condition.	12
**Appendix Table S1.** Demographic information of the MS and non-IDD patients	13

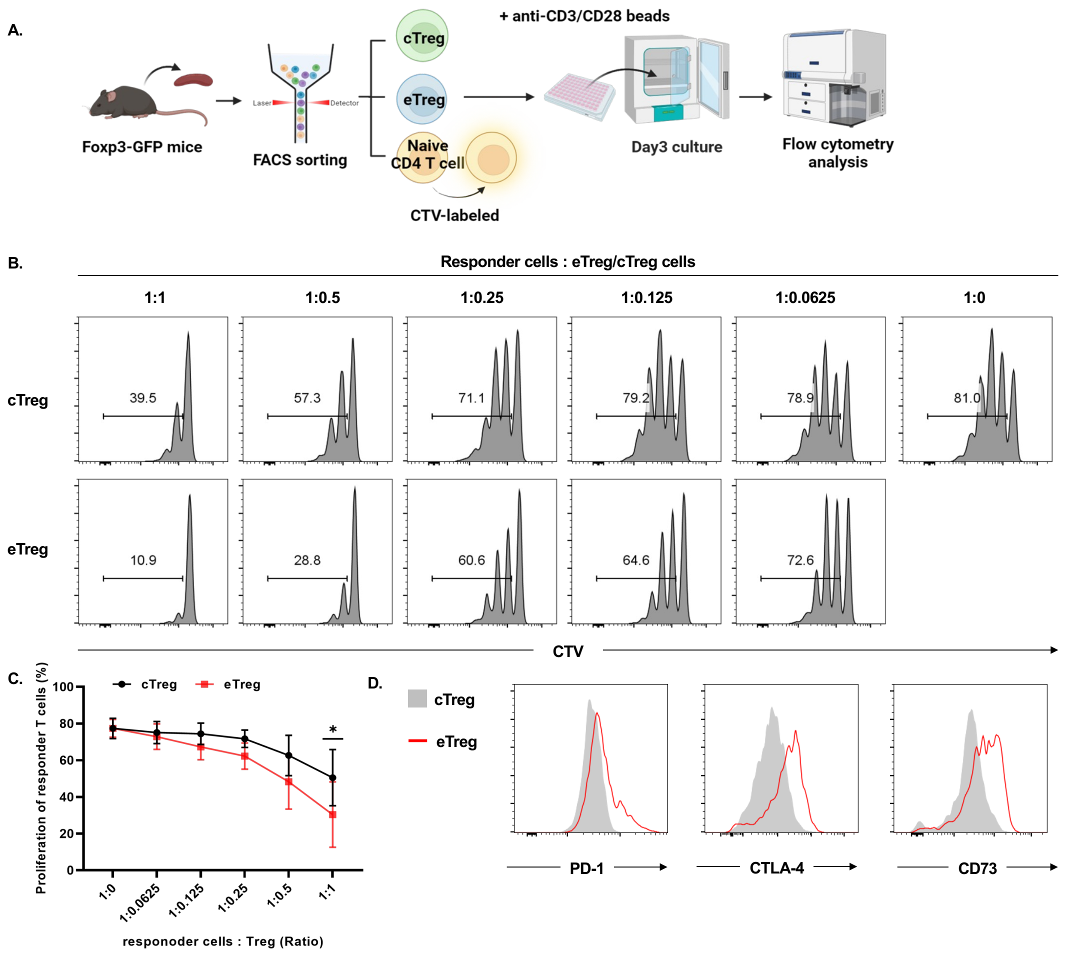

Appendix Figure S1. eTreg cells exhibit higher suppressive function compared to Treg cells in vitro.

(A-C) eTreg cells (TCRβ^{+} CD4^{+} Foxp3^{+} CD62L^{lo} CD44^{hi}) and cTreg cells (TCRβ^{+} CD4^{+} Foxp3^{+} CD62L^{hi} CD44^{lo}) were sorted from Foxp3-GFP mice using FACS. CTV (CellTrace Violet)-labeled FACS sorted naïve CD4 responder T cells (TCRβ^{+} CD4^{+} Foxp3^{-} CD62L^{hi} CD44^{lo}) were co-cultured with eTreg cells and cTreg cells, respectively, with indicated ratio in stimulation by CD3/28 dynabeads for 3 days. (A) Experimental scheme for Treg suppression assay. (B-C) Representative CTV histogram of responder T cells culture under each culture condition. (C) Suppression of proliferation (%) was analyzed by flow cytometry. (D) Expression of CD25, CTLA-4 and CD39 in cTreg cells and eTreg cells. Data are presented as the mean ± S.D. Statistical significance was determined by Two-Way ANOVA. ns = nonsignificant, *p<0.05.

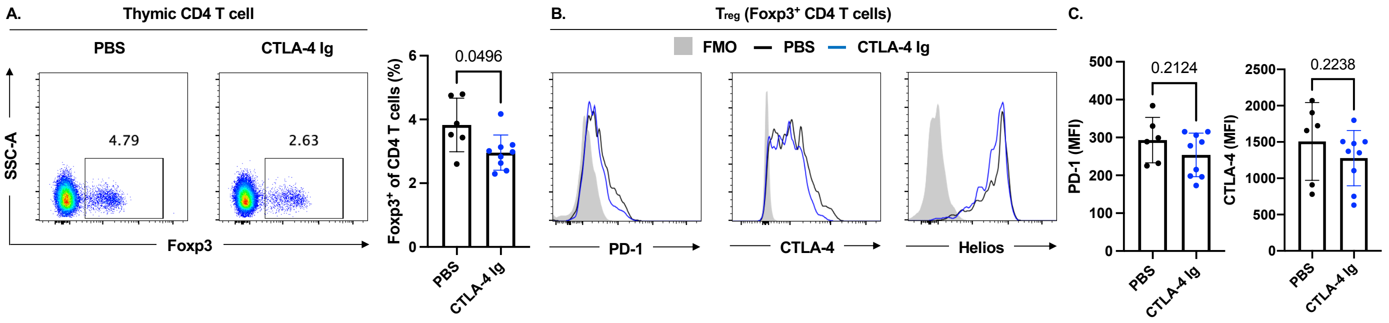

Appendix Figure S2. Costimulation blockade by CTLA-4 Ig reduces thymic Treg cells in steady-state mice.

(A-C) Intact C57BL/6 mice were intraperitoneally injected with 400 µg of CTLA-4 Ig every other day from day 0 to 6 and analyzed at day 7 ($n = 6$ (PBS), $n = 9$ (CTLA-4 Ig)). (A) Expression of Foxp3 in CD4 T cells from thymus was analysis by flow cytometry. (B) Representative histogram of PD-1, CTLA-4, and Helios in thymic Foxp3$^+$ CD4 T cells. (C) Bar graph of MFI of PD-1 and CTLA-4. Data are collated from 3 independent experiments. Data are presented as mean ± S.D. Statistical significance was determined by Mann-Whitney t-test. ns = nonsignificant, *p<0.05, **p<0.01. Data are collated from 3 independent experiments. Statistical significance was determined by nonparametric Mann-Whitney test. ns = nonsignificant, *p<0.05, **p<0.01.

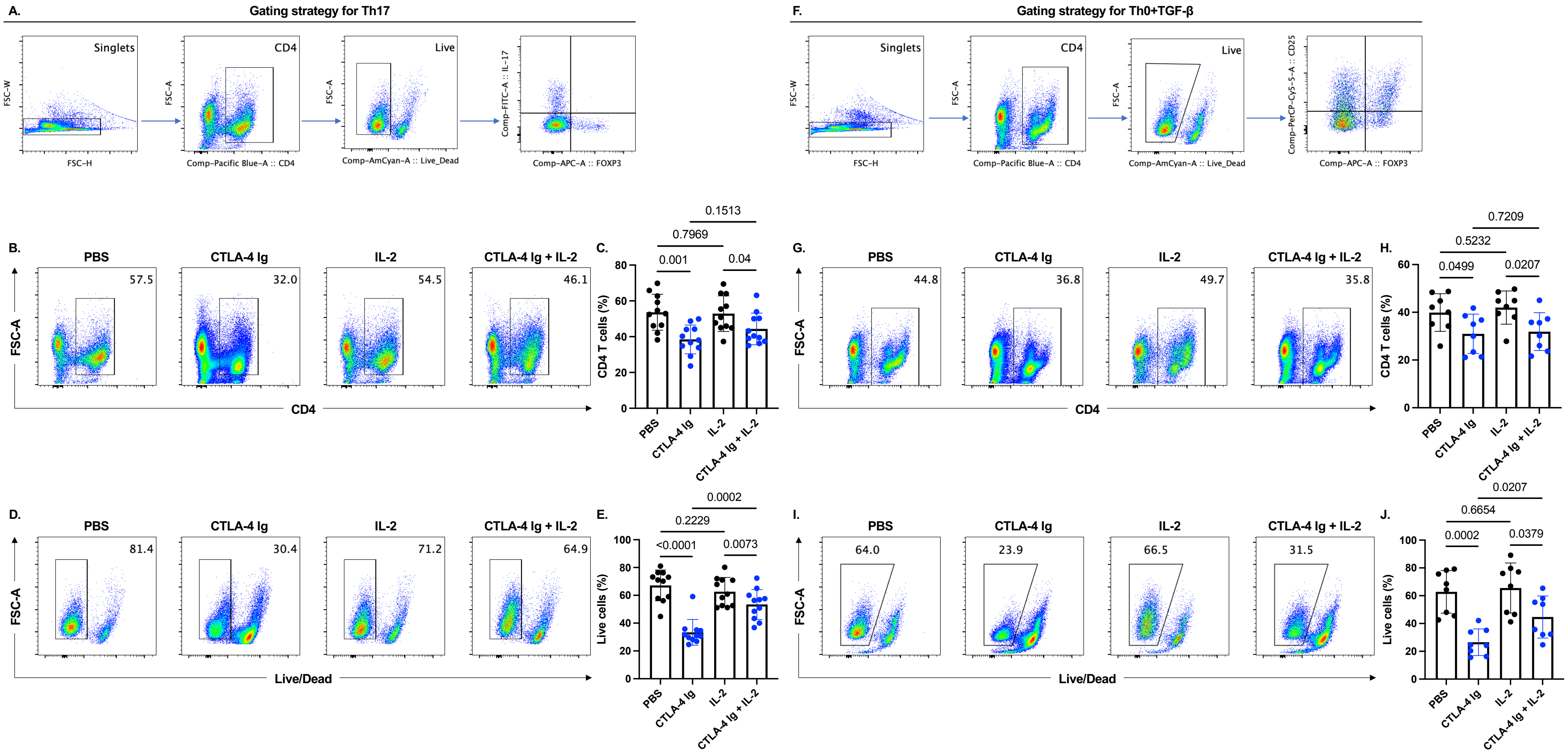

Appendix Figure S3. Gating strategies and effects of CTLA-4 Ig on CD4^{+} T cells in in vitro co-culture system.

Naïve CD4 T cells sorted from 2D2 transgenic mice were cultured with irradiated APCs and stimulated by MOG$_{35\text{-}55}$ antigenic peptide in presence of (A-E) IL-6+TGF-β (Th17, *n=11*) or (F-J) TGF-β (Th0+TGF-β, *n=8*), with or without 50 U/ml of IL-2, treatment of CTLA-4 Ig (0.5 μM) for 3 days. (A) Gating strategy for Th17-polarizing condition. (B) Representative FACS dot plot and (C) quantification of the proportion of CD4^{+} T cells. (D) Representative FACS dot plot and (E) quantification of live cells within the CD4^{+} T cell population. (F) Gating strategy for Th0+TGF-β condition. (G) Representative FACS dot plot and (H) quantification of the proportion of CD4^{+} T cells. (I) Representative FACS dot plot and (J) quantification of live cells within the CD4^{+} T cell population. Data are presented as the mean ± S.D. Statistical significance was determined by nonparametric Mann-Whitney test. ns = nonsignificant, *p < 0.05, **p < 0.01, ***p<0.001, ****p<0.0001.

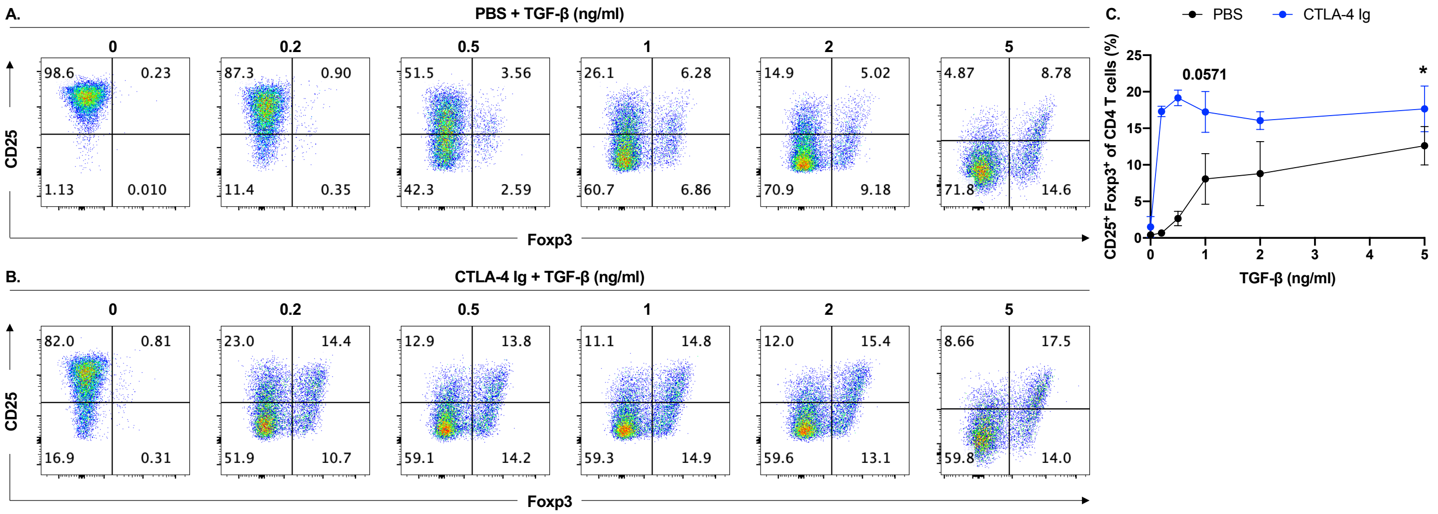

Appendix Figure S4. CTLA-4 Ig induces Treg differentiation under minimal TGF-β conditions without dose dependency.

(A, B) Naïve CD4 T cells sorted from 2D2 transgenic mice were cultured with irradiated APCs and stimulated by 20 μg/ml of MOG_{35-55} antigenic peptide with 0~5 ng/ml of TGF-β in presence of PBS (A) or CTLA-4 Ig (0.5 μM) (B) for 3 days (*n = 2-5*). (C) Proportion of $CD25^{+}$ $Foxp3^{+}$ iTreg cells. Data are presented as mean ± S.D. Statistical significance was determined by Two-Way ANOVA. ns = nonsignificant, *p<0.05, **p<0.01, ***p<0.001.

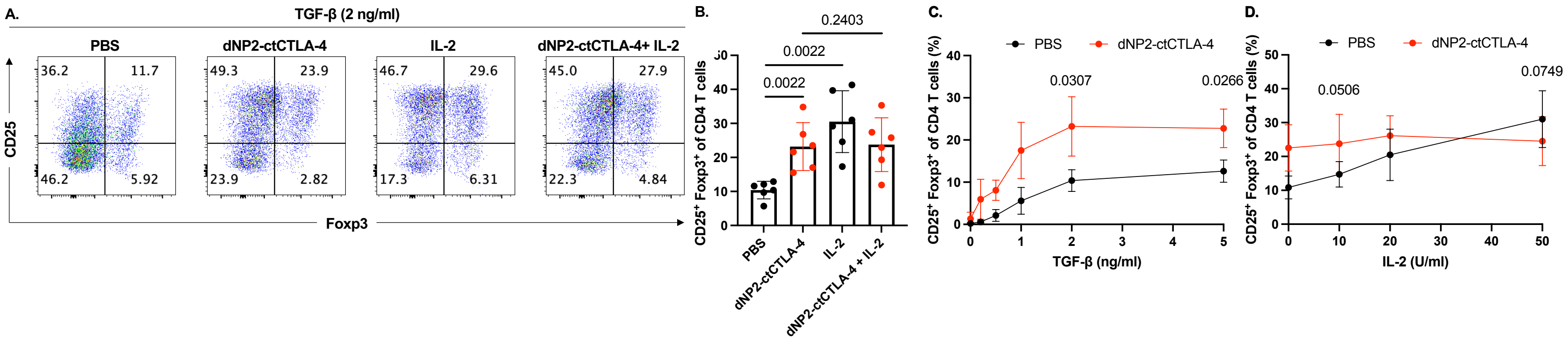

Appendix Figure S5. dNP2-ctCTLA-4 enhances TGF-β-dependent, but not IL-2-driven, Foxp3 induction.

(A- D) Naïve CD4 T cells sorted from 2D2 transgenic mice were cultured with irradiated APCs and stimulated by MOG_{35-55} antigenic peptide in presence of (A-D) TGF-β (Th0+TGF-β) for 3 days ($n = 6$). (A) Representative dot plot of $CD25^+$ $Foxp3^+$ CD4 T cells and (B) bar graph. (C) Dose dependency of TGF-β of $CD25^+$ $Foxp3^+$ Treg induction by dNP2-ctCTLA-4 in absence of IL-2. (D) Dose dependency of IL-2 of $CD25^+$ $Foxp3^+$ Treg induction by dNP2-ctCTLA-4 in fixed concentration of TGF-β. Data are presented as mean ± S.D. Statistical significance was determined by nonparametric Mann-Whitney test. ns = nonsignificant, *p < 0.05, **p < 0.01.

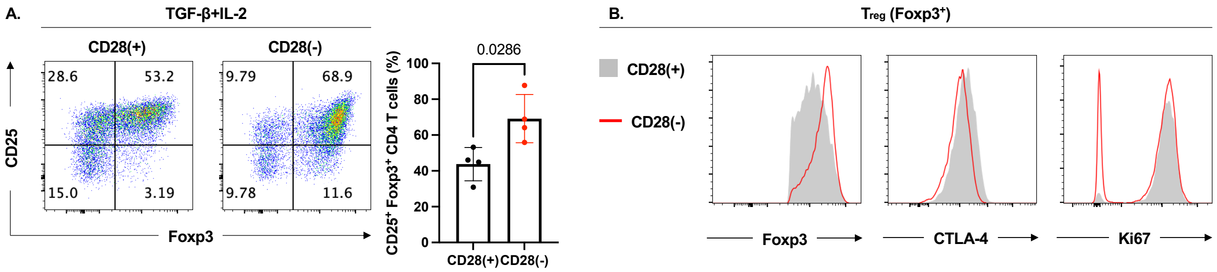

Appendix Figure S6. IL-2 promotes selective Treg induction under low-costimulation conditions.

(A, B) FACS sorted naïve CD4 T cells (CD4^{+} CD25^{-} CD62L^{+} CD44^{-}) were stimulated with anti-CD3 (5 ug/ml) with or not anti-CD28 (5 μg/ml) in presence of TGF-β (2 ng/ml) with or not IL-2 (50 U/ml) for 3 days (*n* = *4*). (A) Representative FACS dot plot of Foxp3^{+} CD25^{+} CD4 T cells and (B) bar graph. Data are presented as mean ± S.D. Statistical significance was determined by nonparametric Mann-Whitney test. ns = nonsignificant, *p<0.05.

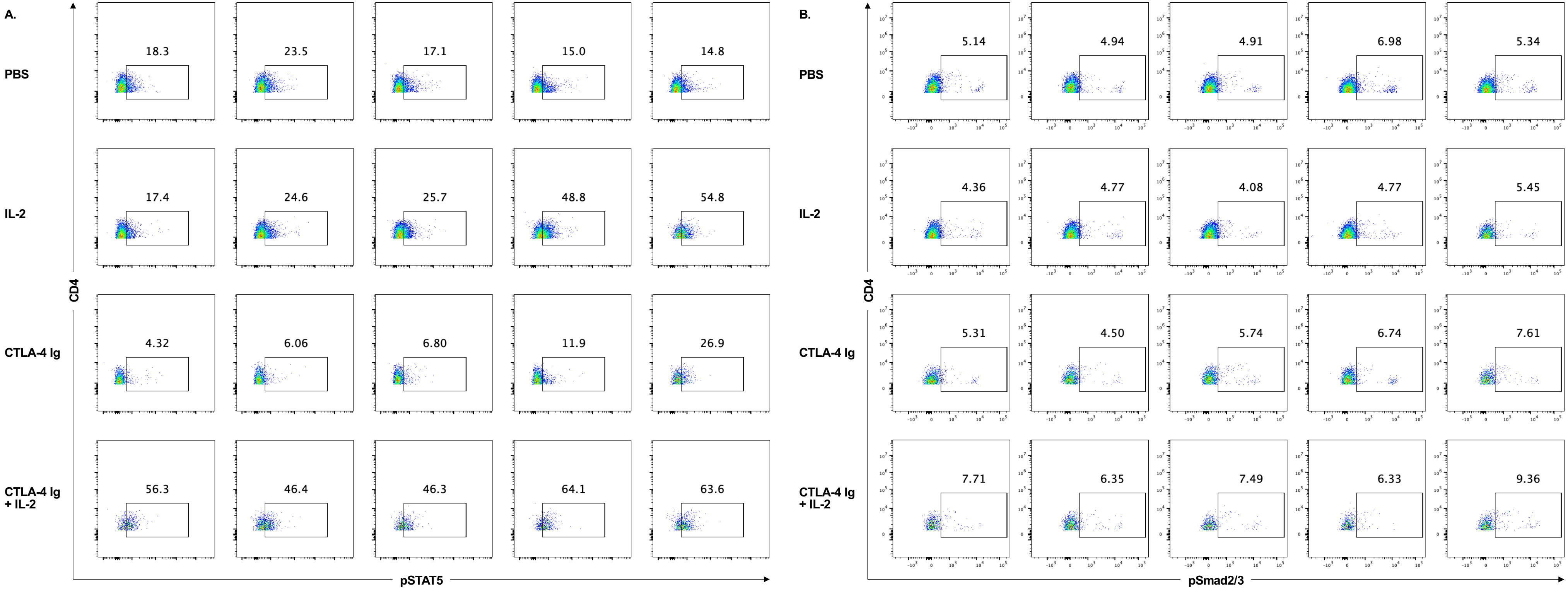

Appendix Figure S7. FACS dot plots of signaling molecules in each group of EAE experiment.

(A, B) 10-weeks-old C57BL/6 female mice were subcutaneously immunized with $MOG_{35\text{-}55}$ peptide (100 µg / flank) emulsified in CFA together with PTX. From day 0 after disease induction, mice received CTLA-4 Ig (200 µg), IL-2 (20 ng) or a combination of CTLA-4 Ig and IL-2 until day 7. FACS dot plots of pSTAT5 (A) and pSmad2/3 C-terminal (B) in $Foxp3^{+}$ CD4 T cells are shown.

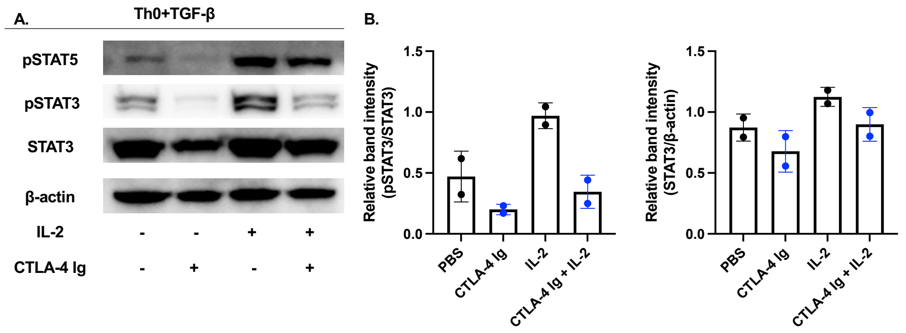

Appendix Figure S8. CTLA-4 Ig consistently reduces pSTAT3 levels when normalized to either STAT3 or β-actin.

(A, B) Naïve CD4 T cells sorted from 2D2 transgenic mice were cultured with irradiated APCs and stimulated by 20 µg/ml of $MOG_{35\text{-}55}$ antigenic peptide with 2 ng/ml of TGF-β (Th0+ TGF-β) or TGF-β with 50 U/ml of IL-2 in presence of CTLA-4 Ig (0.5 µM) for 3 days ($n = 2$). (A) Immunoblotting of pSTAT5, pSTAT3 and STAT3. (B) Representative change in band intensity of pSTAT3 normalized with STAT3 and STAT3 normalized with β-actin. Data are presented as the mean ± S.D.

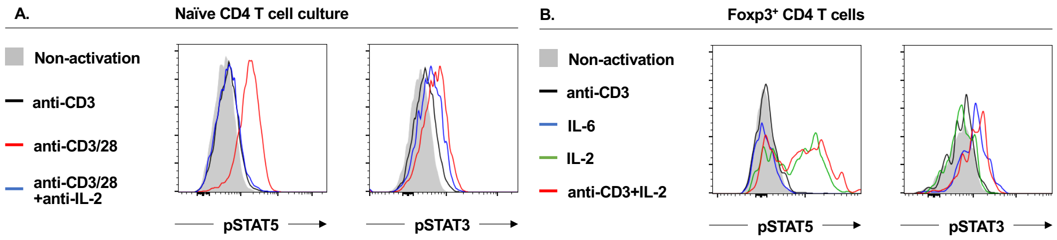

Appendix Figure S9. STAT3 phosphorylation by IL-2 requires T cell receptor stimulation.

(A) FACS-sorted naïve CD4 T cells were cultured with anti-CD3 (2 μg/ml) anti-CD3 + anti-CD28 (5 μg/ml) or anti-CD3 + anti-CD28 with anti-IL-2 neutralizing antibody (5 μg/ml) for 3 days. The expression levels of pSTAT5 and pSTAT3. (B) Isolated splenocyte stimulated by anti-CD3 (5 μg/ml), IL-2 (50 U/ml), IL-6 (30 ng/ml) or anti-CD3 + IL-2 for 4 hours. The expression levels of pSTAT5 and pSTAT3 in Foxp3^{+} CD4 T cells.

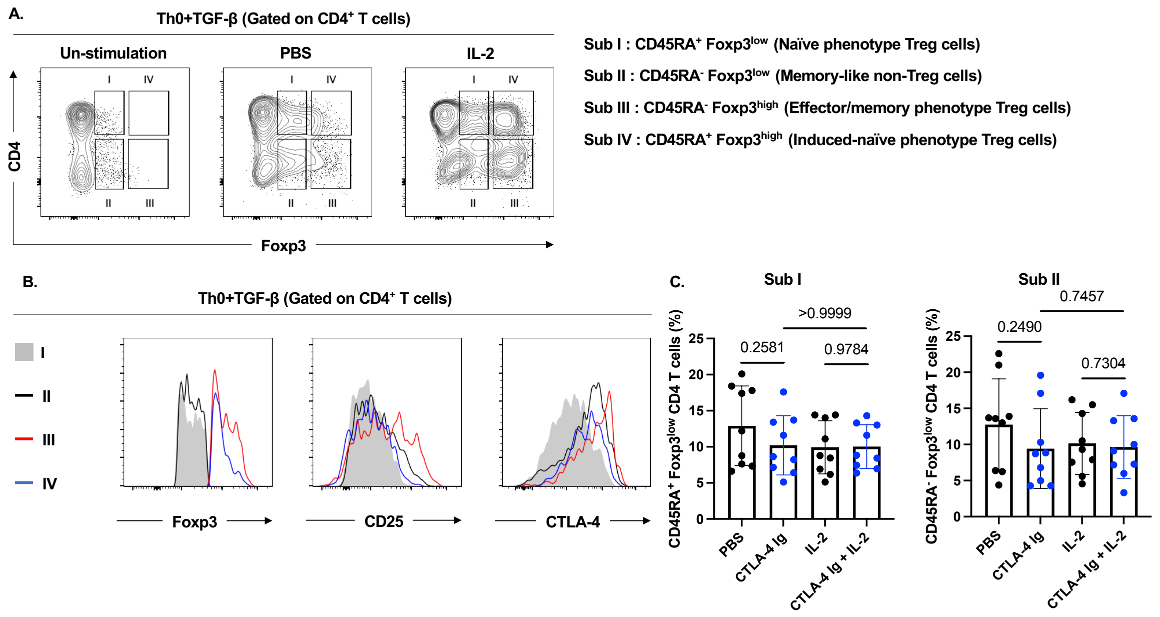

Appendix Figure S10. Classification of human Treg subpopulation in unstimulated and Treg-induced condition.

(A, B) PBMCs from MS patients were stimulated with human anti-CD3 monoclonal antibody under 2 ng/ml of TGF-β with or without 50 u/ml of IL-2. (A, B) 4 subsets CD4 Treg cells based on expression of CD45RA and Foxp3. (A) Representative FACS plot and (B) expression of Fopx3, CD25 and CTLA-4 in each subpopulation. (C) Representative bar graph of Sub I and Sub II population of Treg cells ($n = 9$). Data are presented as mean ± S.D. Statistical significance was determined by nonparametric Mann-Whitney test. ns = nonsignificant.

Age	Sex	Disease	Current treatment	Any other comorbidities
36	F	RRMS	Dimethyl fumarate	None
39	F	RRMS	Natalizumab	None
41	F	RRMS	Natalizumab	None
34	M	RRMS	Natalizumab	None
34	F	RRMS	Interferon-beta	None
35	F	RRMS	Natalizumab	None
52	F	RRMS	Dimethyl fumarate	None
50	M	RRMS	Dimethyl fumarate	None
36	F	RRMS	Fingolimod	None
24	M	RRMS	Natalizumab	None

Age	Sex	Disease	Current treatment	Any other comorbidities
34	M	polyneuropathy, r/o AIDP	Naïve	None
25	F	polyneuropathy, r/o AIDP	Prednisolone	None
22	F	ptosis	Naïve	None
50	F	MG	Naïve	None
43	F	polyneuropathy, CIDP	Monthly immunoglobulin	None
50	M	polyneuropathy, MMNCB	Monthly immunoglobulin	None
23	F	anaplastic astrocytoma	Naïve	None
36	M	MG, ocular	Naïve	None
38	F	blepharospasm	Naïve	None
54	M	MG	Tacrolimus, prednisolone	None

Appendix Table S1. Demographic information of the MS and non-IDD patients

Demographic information of the RRMS and non-IDD patients from Seoul National University Hospital. PBMCs from MS and non-IDD patients analyzed in this study were obtained from 10 donors, respectively.
