# Supplementary figures and images for: Costimulation loss enhances IL-2-driven Treg generation by PI3K-STAT3 inhibition in CNS autoimmunity
Source: EMBO Mol Med. 2026 May 5;18(6):2272–92. doi: 10.1038/s44321-026-00431-7 (PMC13269791; doi:10.1038/s44321-026-00431-7)

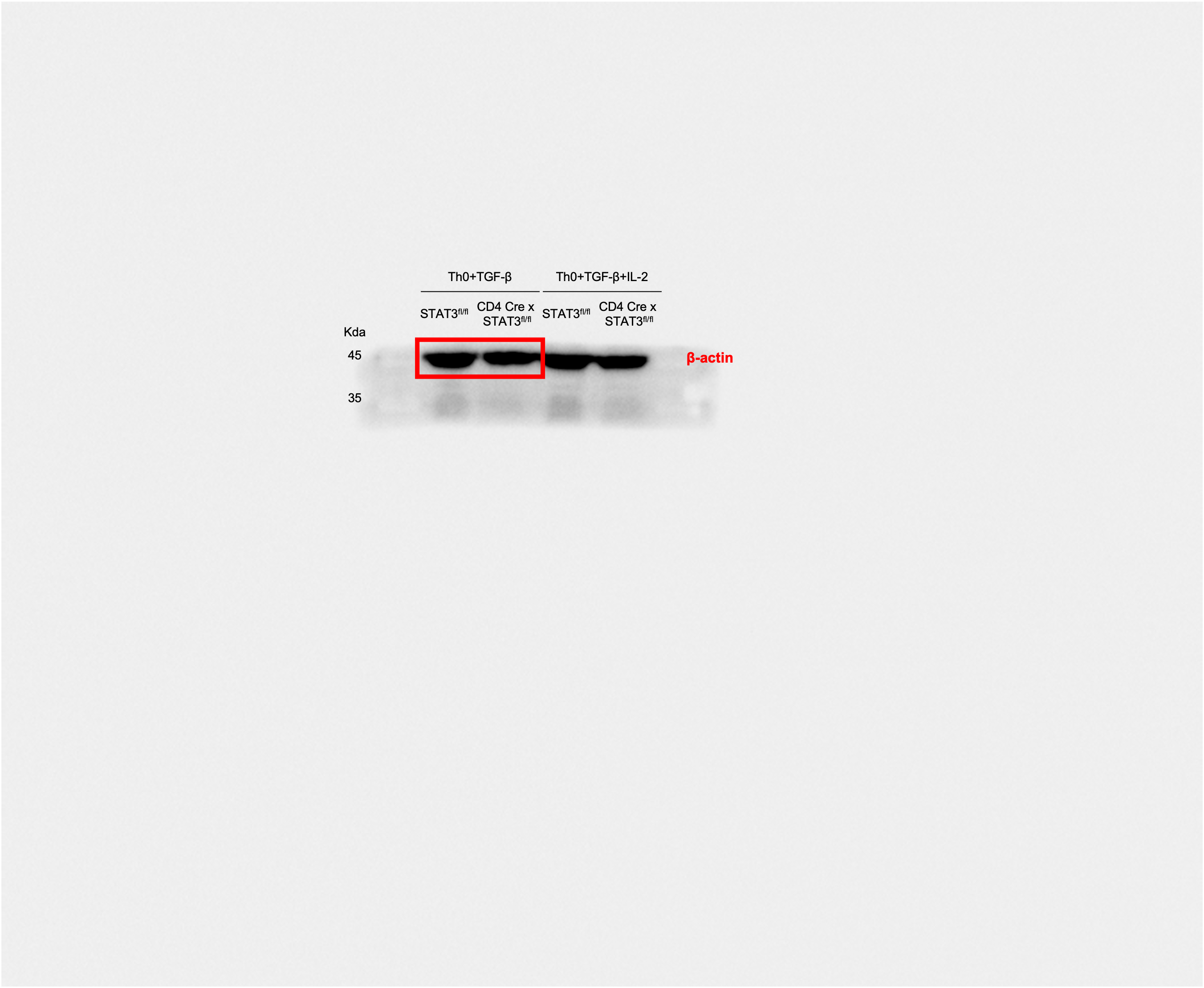

Supplement: Supplementary file 7 — Source data Fig. 4 [file 44321_2026_431_MOESM7_ESM.zip › Figure 4/4E/Western ╬▓-actin with crop box.png]

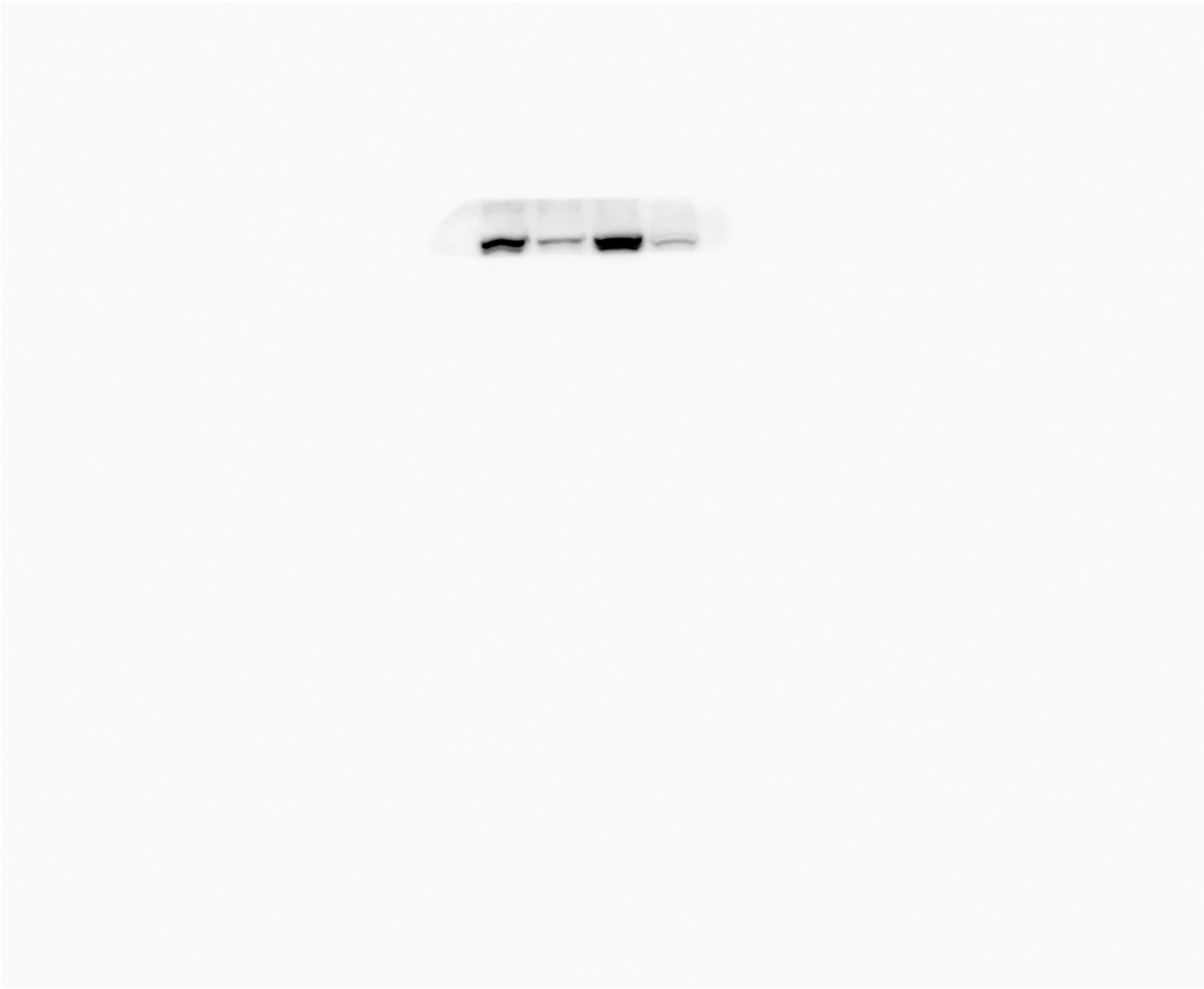

Supplement: Supplementary file 7 — Source data Fig. 4 [file 44321_2026_431_MOESM7_ESM.zip › Figure 4/4E/Western pSTAT3 with crop box.png]

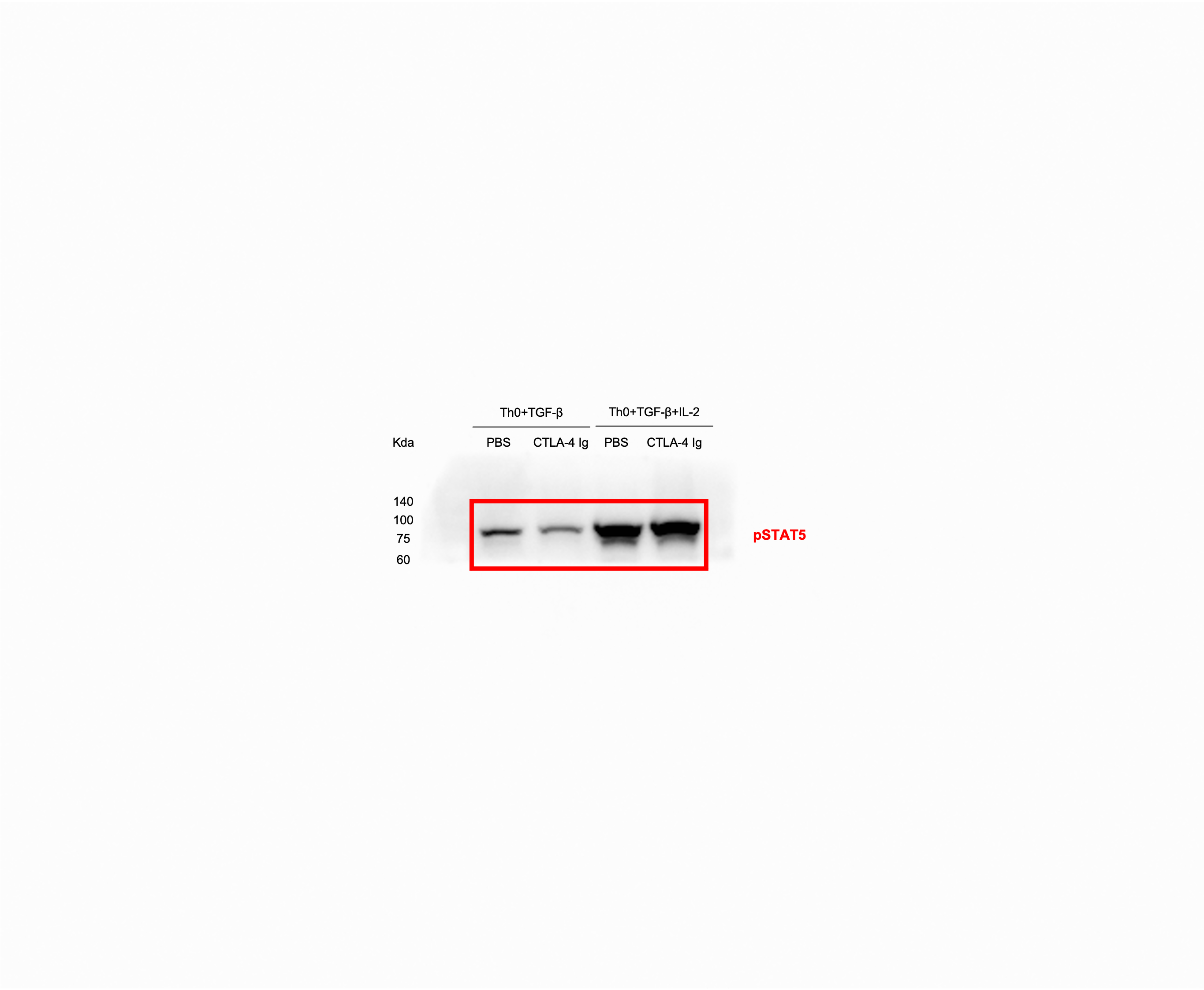

Supplement: Supplementary file 7 — Source data Fig. 4 [file 44321_2026_431_MOESM7_ESM.zip › Figure 4/4A/Western pSTAT5 with crop box.png]

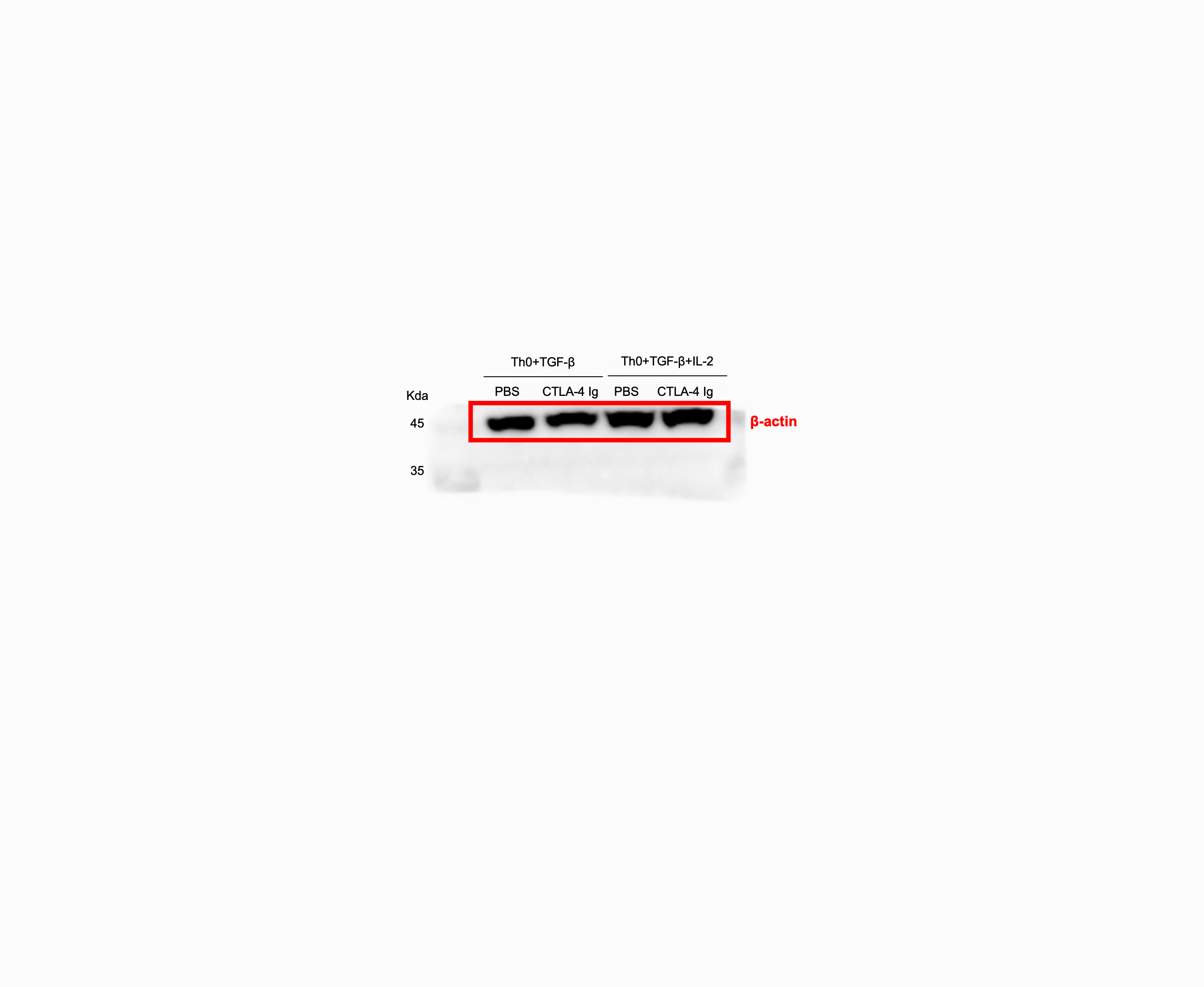

Supplement: Supplementary file 7 — Source data Fig. 4 [file 44321_2026_431_MOESM7_ESM.zip › Figure 4/4A/Western ╬▓-actin with crop box.png]

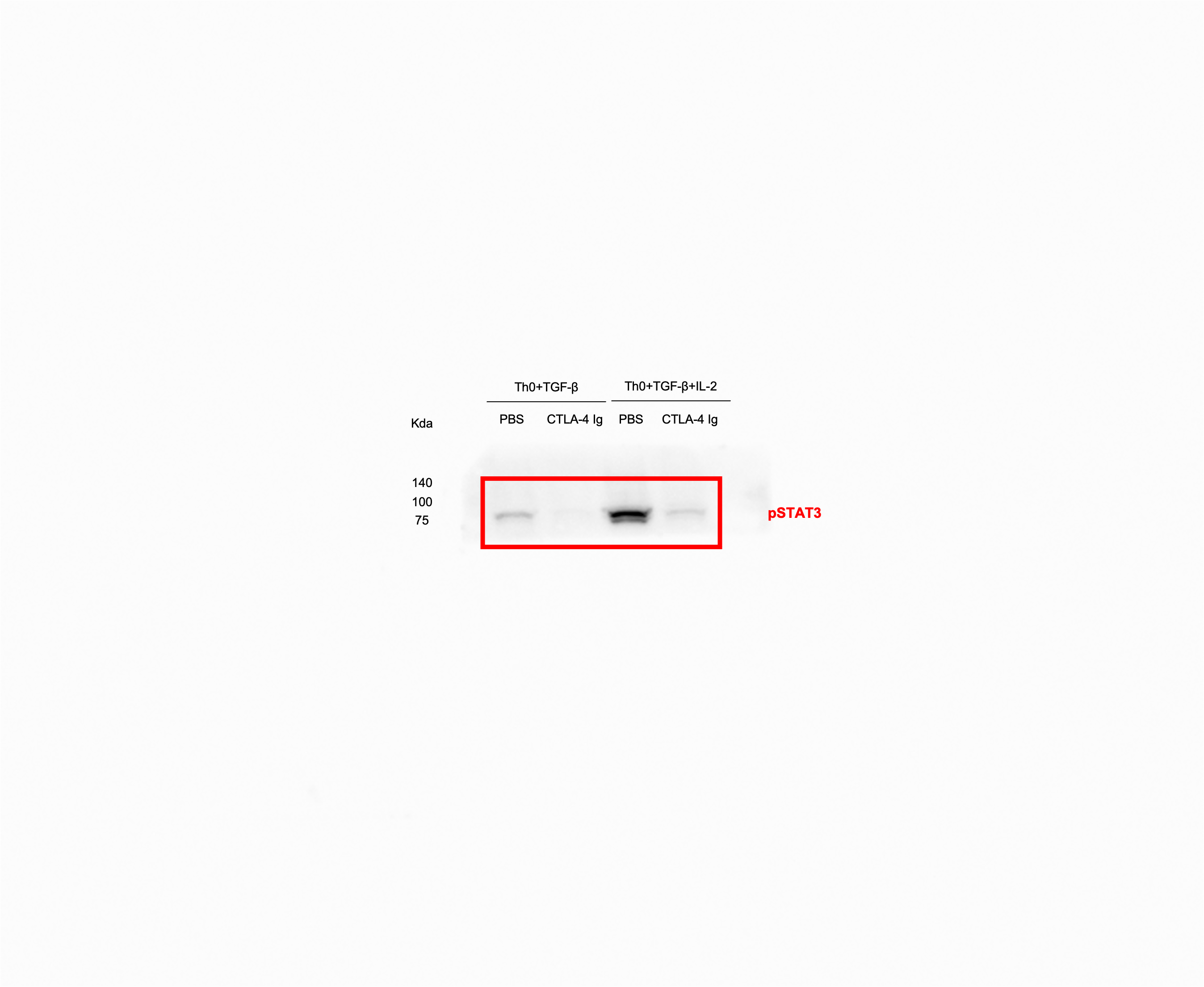

Supplement: Supplementary file 7 — Source data Fig. 4 [file 44321_2026_431_MOESM7_ESM.zip › Figure 4/4A/Western pSTAT3 with crop box.png]

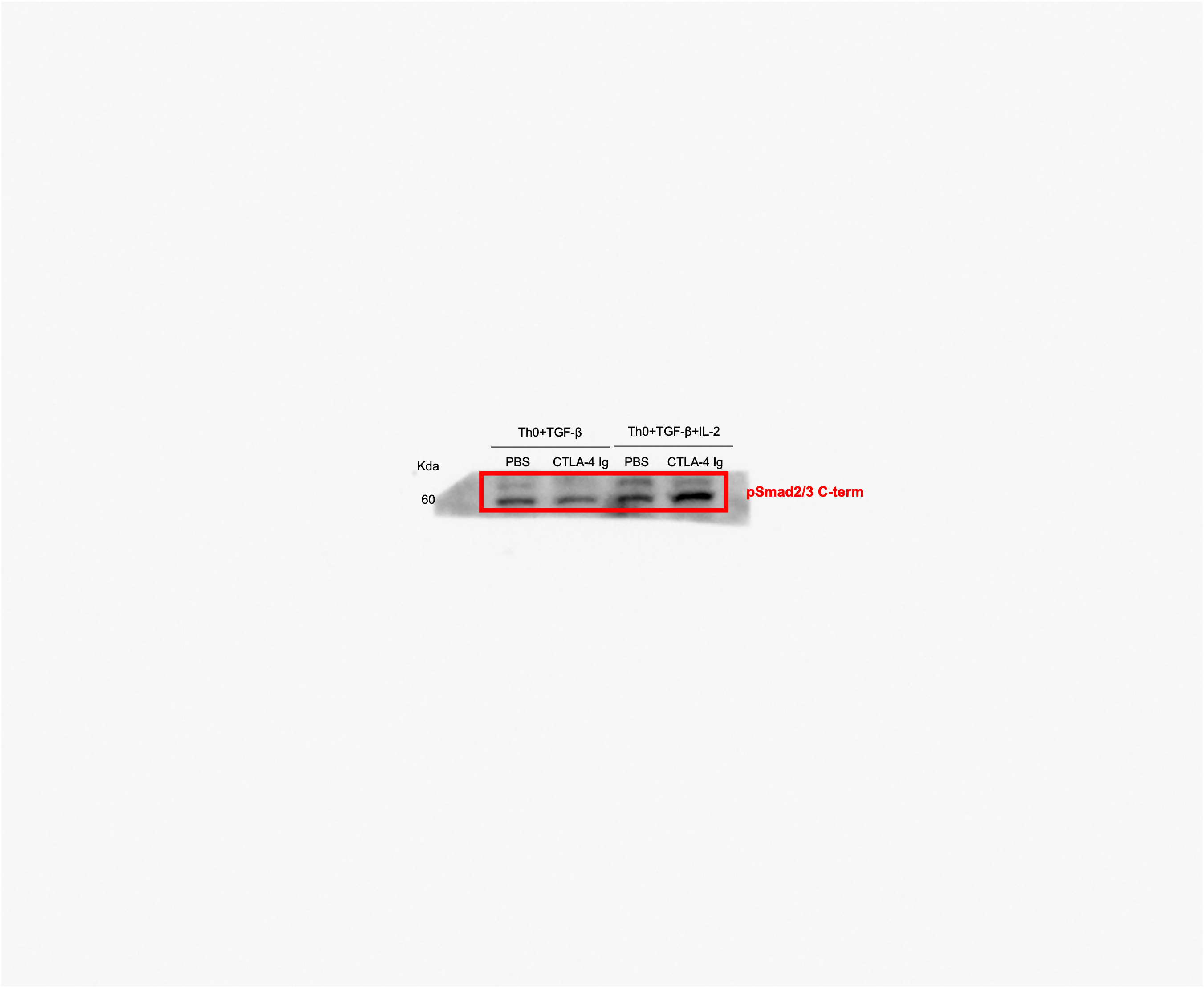

Supplement: Supplementary file 7 — Source data Fig. 4 [file 44321_2026_431_MOESM7_ESM.zip › Figure 4/4A/Western pSmad2_3 C-term with crop box.png]

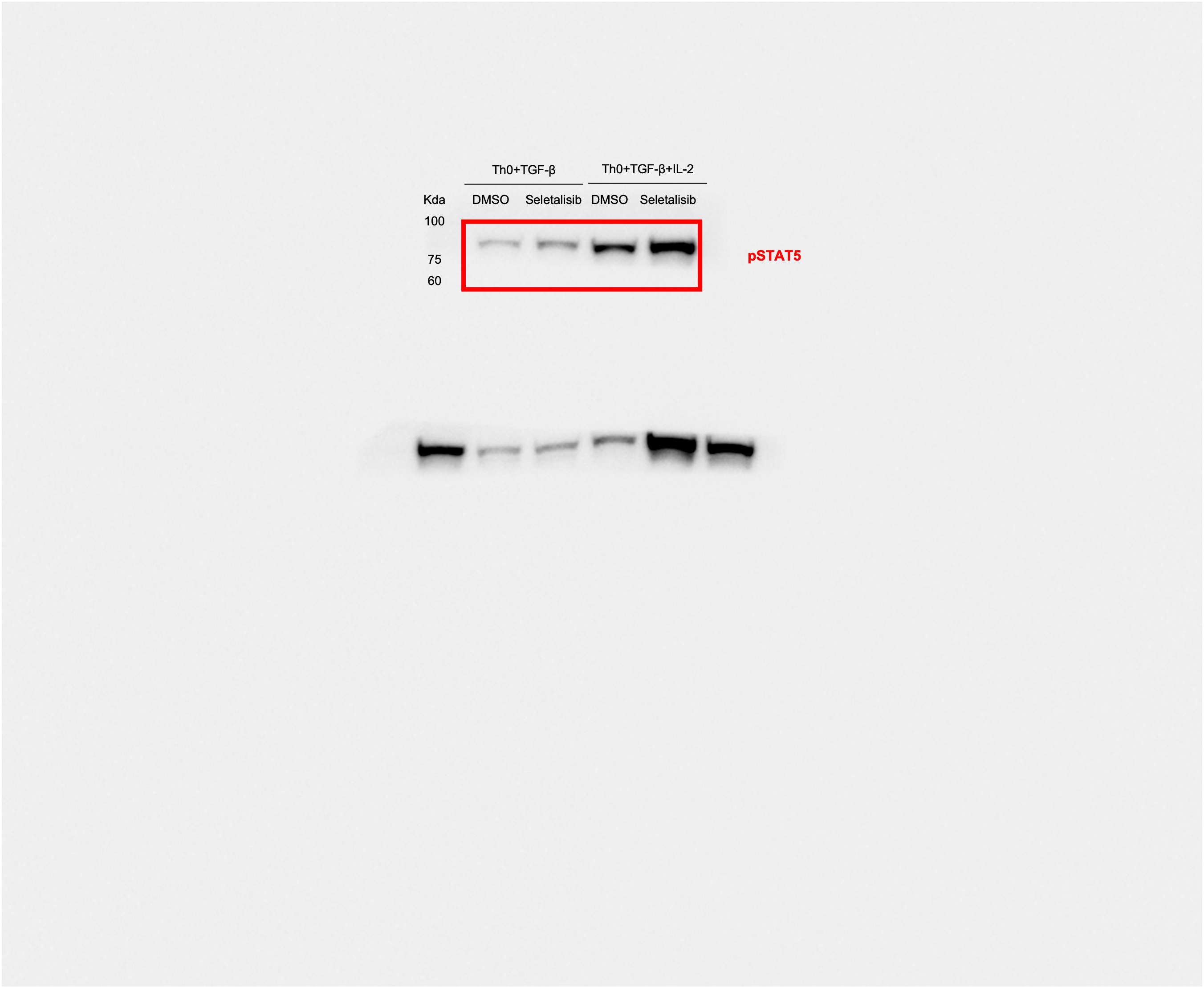

Supplement: Supplementary file 7 — Source data Fig. 4 [file 44321_2026_431_MOESM7_ESM.zip › Figure 4/4H/Western pSTAT5 with crop box.png]

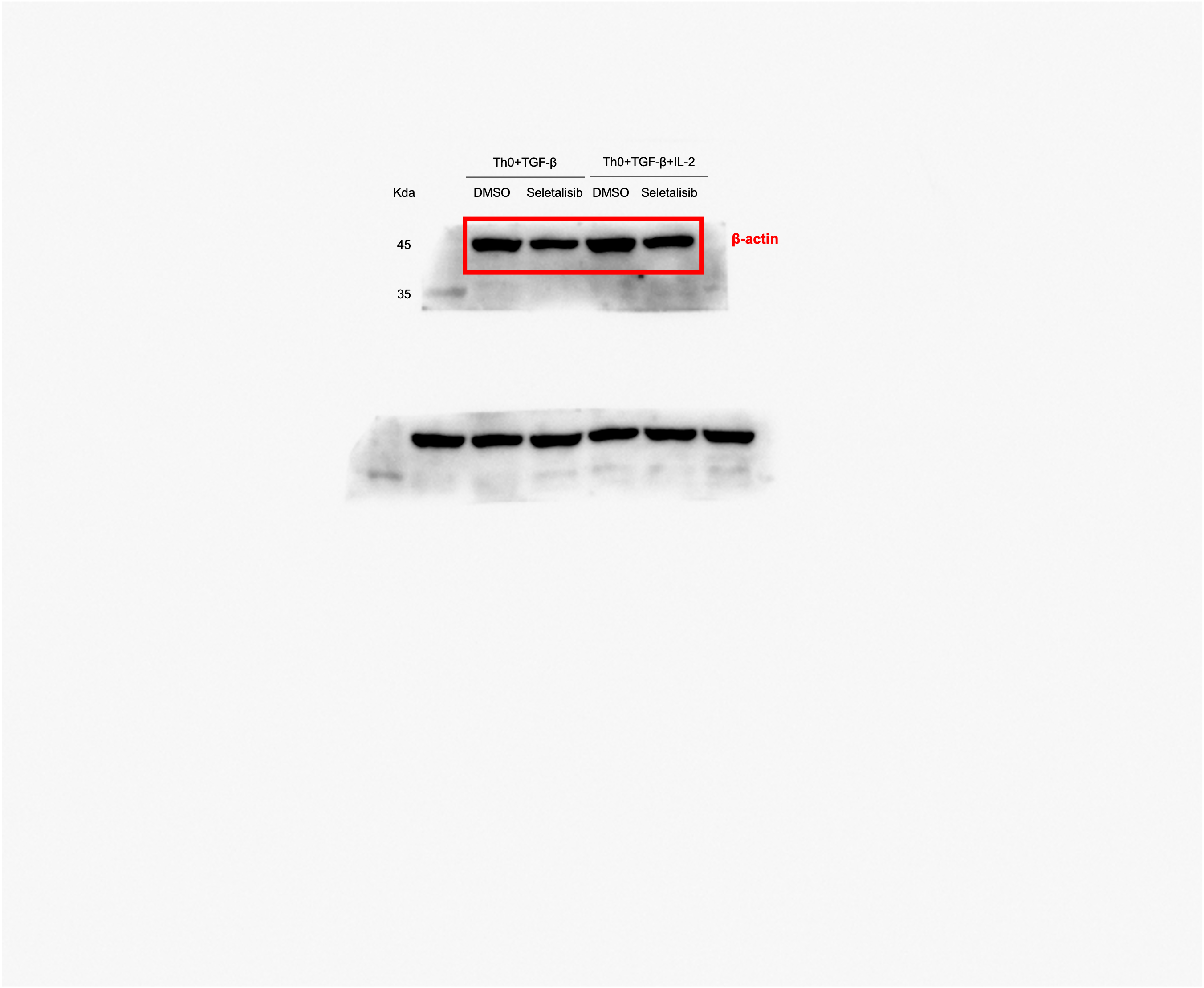

Supplement: Supplementary file 7 — Source data Fig. 4 [file 44321_2026_431_MOESM7_ESM.zip › Figure 4/4H/Western ╬▓-actin with crop box.png]

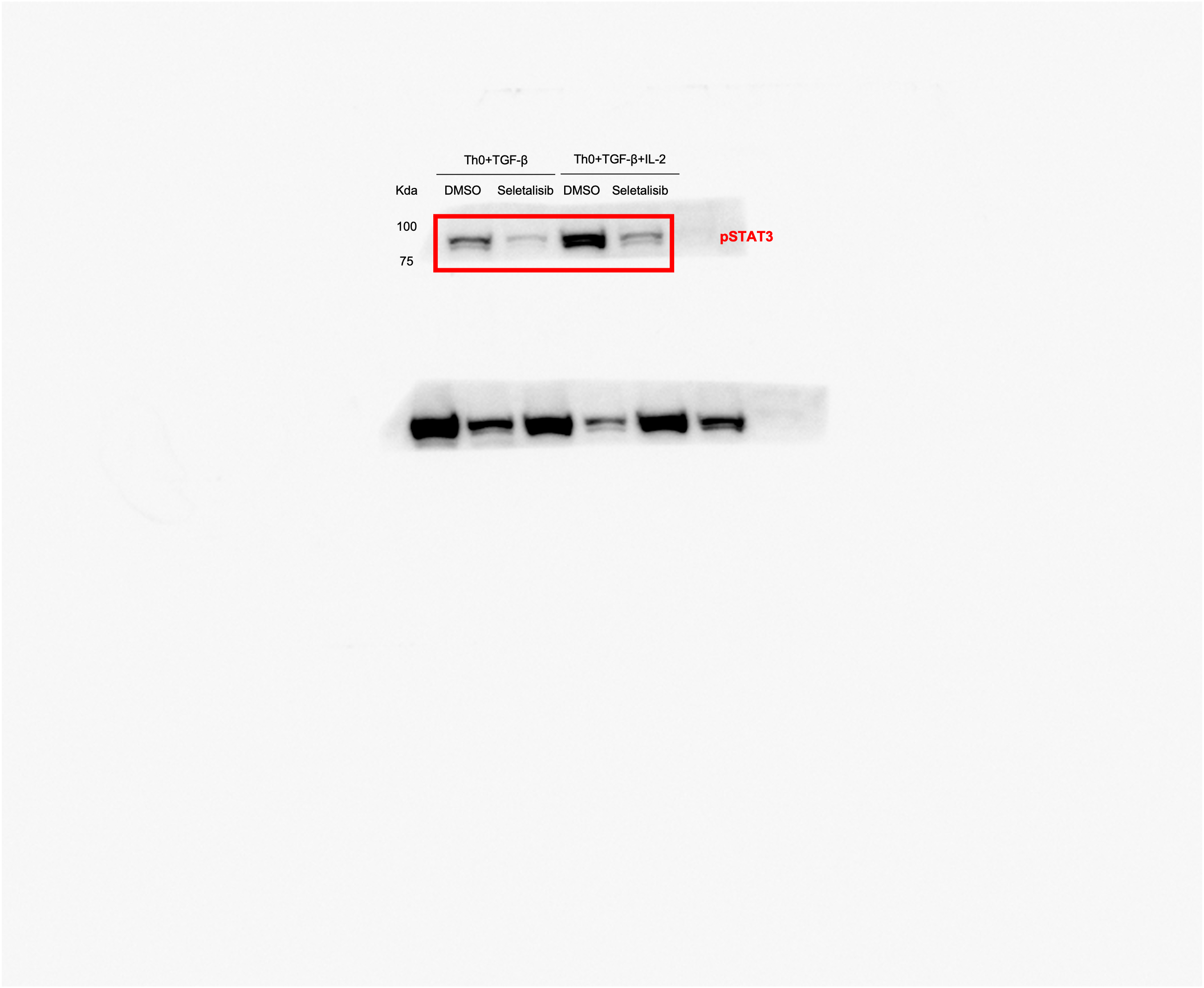

Supplement: Supplementary file 7 — Source data Fig. 4 [file 44321_2026_431_MOESM7_ESM.zip › Figure 4/4H/Western pSTAT3 with crop box.png]

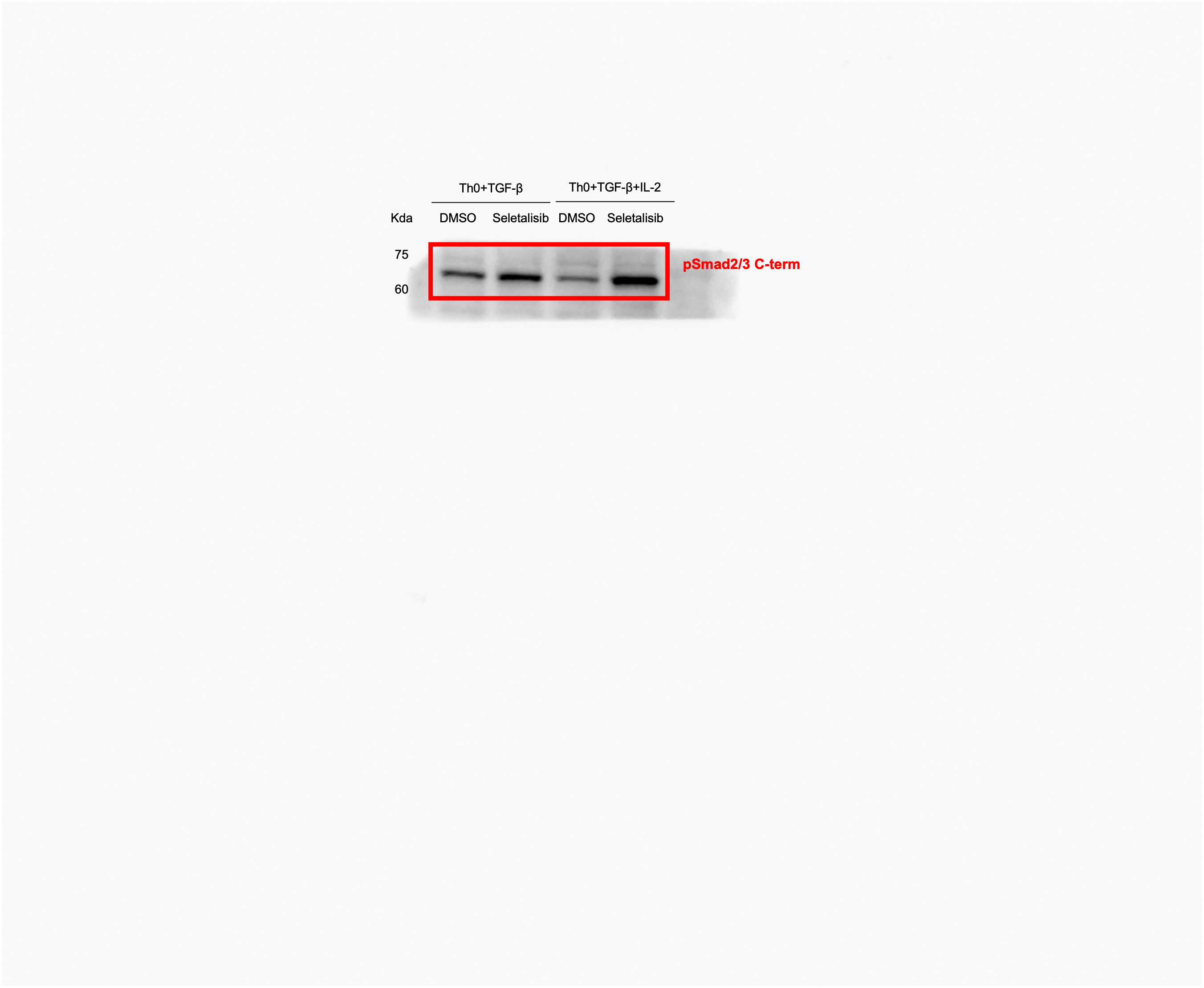

Supplement: Supplementary file 7 — Source data Fig. 4 [file 44321_2026_431_MOESM7_ESM.zip › Figure 4/4H/Western pSmad2_3 C-term with crop box.png]
