# Supplementary material for: Costimulation loss enhances IL-2-driven Treg generation by PI3K-STAT3 inhibition in CNS autoimmunity
Source: EMBO Mol Med. 2026 May 5;18(6):2272–92. doi: 10.1038/s44321-026-00431-7 (PMC13269791; doi:10.1038/s44321-026-00431-7)
Supplement: Supplementary file 9 — Expanded View Figures [file 44321_2026_431_MOESM9_ESM.pdf]

Expanded View Figures

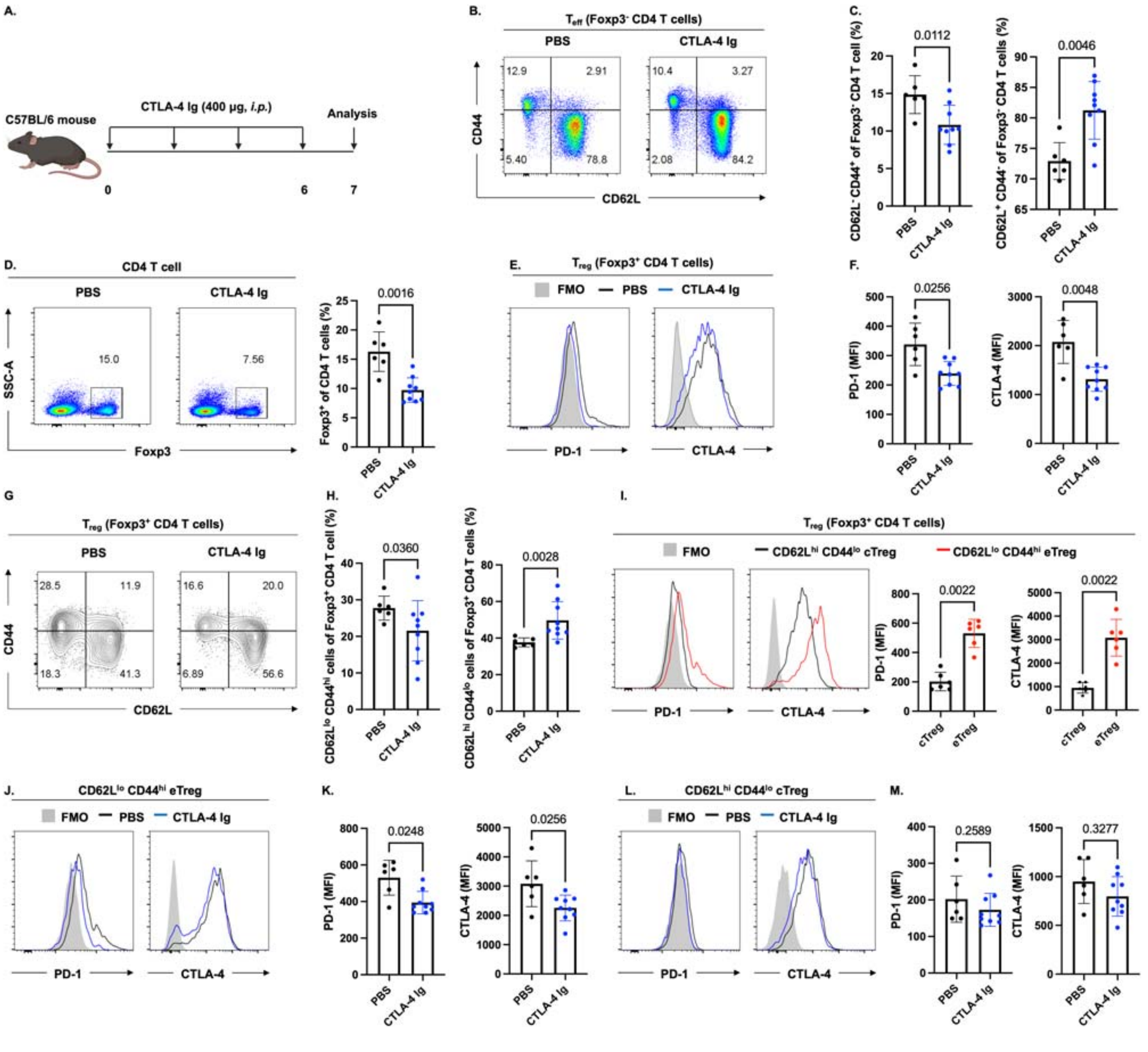

Figure EV1. Costimulation blockade by CTLA-4 Ig reduces proportion of effector/memory CD4 T cells and eTreg cells in steady-state mice.

(**A–M**) Intact C57BL/6 mice were intraperitoneally injected with 400 μg of CTLA-4 Ig every other day from day 0 to 6 and analyzed at day 7 ($n = 6$ (PBS), $n = 9$ (CTLA-4 Ig)). (**B**, **C**) Naive and effector/memory phenotypic change in CD4 T cells of spleen by CTLA-4 Ig analyzed in flow cytometry. (**D**) Expression of Foxp3 in CD4 T cells was analyzed by flow cytometry. (**E**, **F**) PD-1 and CTLA-4 expression in Foxp3^{+} CD4 T cells. (**E**) Representative histogram of CTLA-4 and PD-1. (**F**) Bar graph of MFI of PD-1 and CTLA-4. (**G**, **H**) CD62L^{lo} CD44^{hi} and CD62L^{hi} CD44^{lo} subpopulation of Foxp3^{+} CD4 T cells. (**I**) Comparison of Immuno-suppressive molecules, including PD-1 and CTLA-4 and expression in CD62L^{lo} CD44^{hi} Foxp3^{+} eTreg cells and CD62L^{hi} CD44^{lo} Foxp3^{+} cTreg cells were analyzed by flow cytometry. (**J–M**) Expression changes of PD-1 and CTLA-4 and expression in CD62L^{lo} CD44^{hi} Foxp3^{+} eTreg cells (**J**, **K**) and CD62L^{hi} CD44^{lo} Foxp3^{+} cTreg cells by CTLA-4 Ig (**L**, **M**). Data are collated from three independent experiments. Data are presented as mean ± SD. Statistical significance was determined by nonparametric Mann–Whitney test. ns = nonsignificant, *$P < 0.05$, **$P < 0.01$.

 © The Author(s)

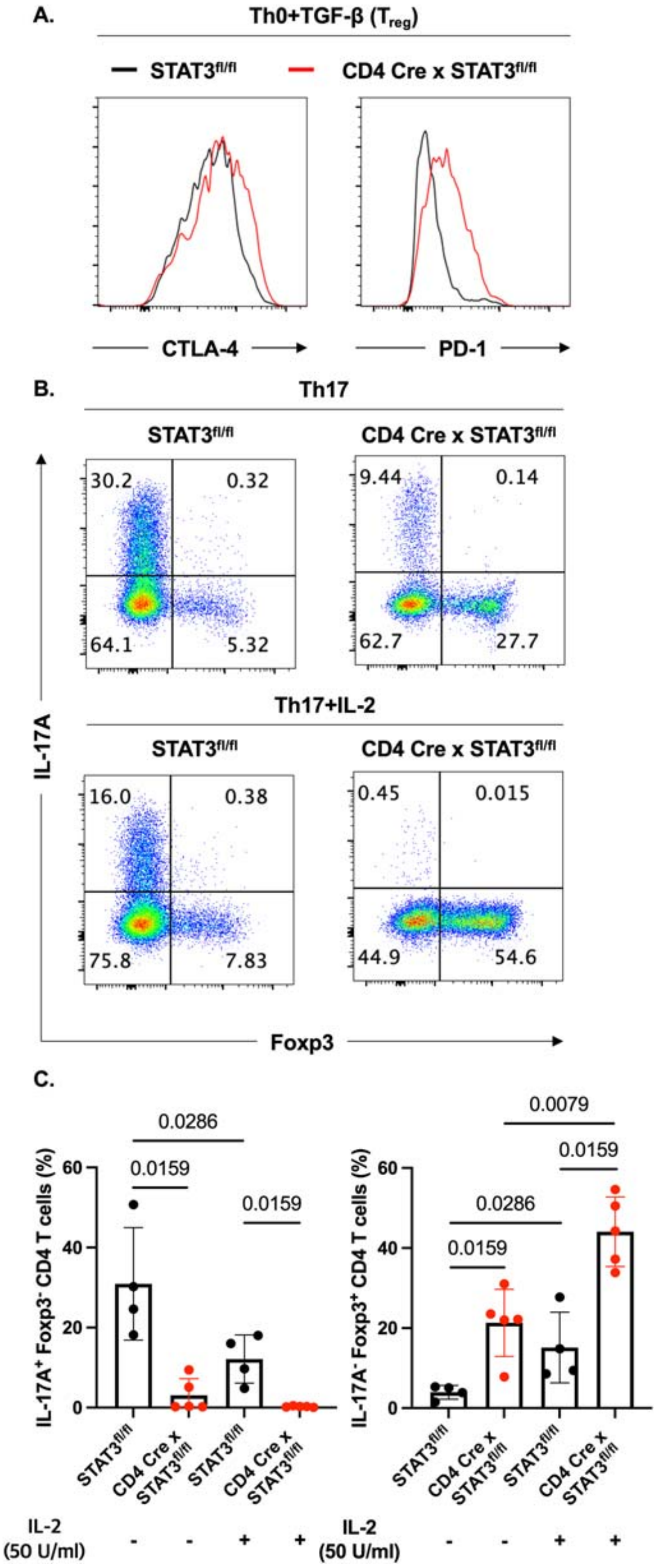
A.
Th0+TGF-β (Treg)
STAT3fl/fl
CD4 Cre x STAT3fl/fl
CTLA-4
PD-1
B.
Th17
STAT3fl/fl
CD4 Cre x STAT3fl/fl
30.2
0.32
64.1
5.32
9.44
0.14
62.7
27.7
Th17+IL-2
STAT3fl/fl
CD4 Cre x STAT3fl/fl
16.0
0.38
75.8
7.83
0.45
0.015
44.9
54.6
IL-17A
Foxp3
C.
IL-17A+ Foxp3- CD4 T cells (%)
0.0286
0.0159
0.0159
IL-17A- Foxp3+ CD4 T cells (%)
0.0079
0.0159
0.0286
0.0159
STAT3fl/fl
CD4 Cre x STAT3fl/fl
IL-2 (50 U/ml)
- - + +

Figure EV2. STAT3 deficiency enhances Foxp3 expression and suppresses Th17 differentiation in the presence of IL-2.

(**A**) Representative histogram of CTLA-4 and PD-1 in Foxp3$^+$ CD4 T cells in the presence of TGF-β condition. (**B**, **C**) Splenocyte from STAT3$^{fl/fl}$ and CD4 Cre x STAT3$^{fl/fl}$ mice were stimulated with a-CD3 (2 μg/ml) in presence of TGF-β (2 ng/ml) and IL-6 (30 ng/ml) with or not IL-2 (50 U/ml) for 3 days ($n = 4$ (STAT3$^{fl/fl}$), $n = 5$ (CD4 Cre x STAT3$^{fl/fl}$)). (**B**) Representative FACS dot plot and (**C**) bar graph of expression of IL-17A and Foxp3 in CD4 T cells. Data are presented as mean ± SD. Statistical significance was determined by nonparametric Mann–Whitney test. ns = nonsignificant, *$P < 0.05$, **$P < 0.01$.

 © The Author(s)

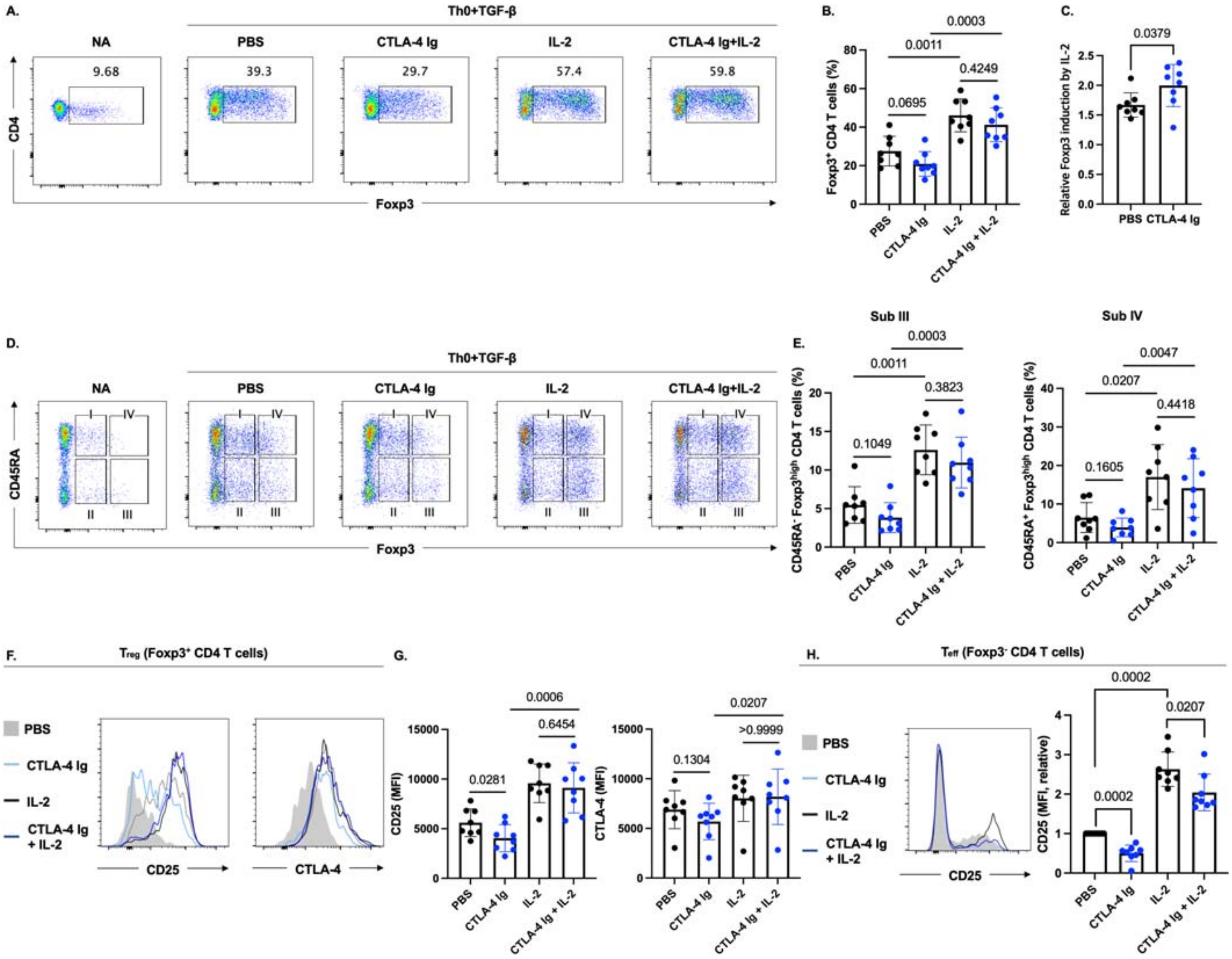

Figure EV3. CTLA-4 Ig and IL-2 co-treatment increases Foxp3hi Treg subpopulations and enhances suppressive molecule expression in human PBMCs from non-IDD patients.

(**A–H**) PBMCs from non-IDD patients were stimulated with human anti-CD3 monoclonal antibody under 2 ng/ml of TGF-β with or without 50 U/ml of IL-2 in presence of 1 μM of CTLA-4 Ig for 3 days (*n* = 8 from independent donors). (**A**, **B**) Representative FACS dot plot (**A**) and bar graph (**B**) exhibits Foxp3 induction. (**C**) IL-2 induced Foxp3 ratio with or without CTLA-4 Ig. (**D**, **E**) Representative FACS dot plot (**D**) and bar graph (**E**) of III (Effector/memory phenotype Treg cells) and IV (induced-naive phenotype Treg cells) sub-population of Treg cells. (**F**) Representative histogram (**G**) and bar graph of CD25 and CTLA-4 expression in $Foxp3^+$ CD4 T cells. (**H**) Representative histogram and bar graph of CD25 expression in $Foxp3^-$ effector CD4 T cells. Data are presented as the mean ± SD. Statistical significance was determined by nonparametric Mann–Whitney test. ns = nonsignificant, *$P < 0.05$, **$P < 0.01$, ***$P < 0.001$.
