# Supplementary material for: Costimulation loss enhances IL-2-driven Treg generation by PI3K-STAT3 inhibition in CNS autoimmunity
Source: EMBO Mol Med. 2026 May 5;18(6):2272–92. doi: 10.1038/s44321-026-00431-7 (PMC13269791; doi:10.1038/s44321-026-00431-7)
Supplement: Supplementary file 2 — Peer Review File [file 44321_2026_431_MOESM2_ESM.pdf]

EMBO Molecular Medicine

Costimulation loss enhances IL-2-driven Treg generation by PI3K-STAT3 inhibition in CNS autoimmunity

Je-Min Choi, Kyung-Ho Nam, Gil-Ran Kim, Yu-Rim Kim, Young Nam Kwon, and Sung-Min Kim

Corresponding author: Je-Min Choi (jeminchoi@hanyang.ac.kr)

Review Timeline:

Submission Date:	19th May 25
Editorial Decision:	7th Jul 25
Revision Received:	5th Mar 26
Editorial Decision:	27th Mar 26
Revision Received:	8th Apr 26
Accepted:	8th Apr 26

Editor: Zeljko Durdevic

Transaction Report:

(Note: With the exception of the correction of typographical or spelling errors that could be a source of ambiguity, letters and reports are not edited. Depending on transfer agreements, referee reports obtained elsewhere may or may not be included in this compilation. Referee reports are anonymous unless the Referee chooses to sign their reports.)

7th Jul 2025

Dear Prof. Choi,

*****

Thank you for the submission of your manuscript to EMBO Molecular Medicine, and please accept my apologies for the delay in getting back to you, which is due to the fact that one referee needed more time to complete his/her review. We have now received feedback from the three reviewers who agreed to evaluate your manuscript. As you will see from the reports below, all three referees acknowledge potential interest of the study but also raise serious and partially overlapping concerns, particularly regarding the limited evidence to support main conclusion of the study.

Overall, it is clear that publication of the paper cannot be considered at this stage. I also note that addressing the reviewers concerns in full will be necessary for further considering the manuscript in our journal and this appears to require a lot of additional work and experimentation. I am unsure whether you will be able or willing to address those and return a revised manuscript within the 6 months deadline. On the other hand, given the potential interest of the findings, I would be willing to consider a revised manuscript with the understanding that the referee concerns must be fully addressed.

Please note that further consideration of a revision that addresses reviewers' concerns in full will entail a second round of review. EMBO Molecular Medicine encourages a single round of revision only and therefore, acceptance or rejection of the manuscript will depend on the completeness of your responses included in the next, final version of the manuscript. For this reason, and to save you from any frustrations in the end, I would strongly advise against returning an incomplete revision and would also understand your decision if you chose to rather seek rapid publication elsewhere at this stage. If you would like to discuss further the points raised by the referees, I am available to do so via email or video. Let me know if you are interested in this option.

I look forward to receiving your revised manuscript. Should you find that the requested revisions are not feasible within the constraints outlined here and choose, therefore, to submit your paper elsewhere, we would welcome a message to this effect.

Yours sincerely,

Zeljko Durdevic

Zeljko Durdevic
Senior Editor
EMBO Molecular Medicine

*****

When submitting your revised manuscript, please carefully review the instructions that follow below. We perform an initial quality control of all revised manuscripts before re-review; failure to include requested items will delay the evaluation of your revision.

We require:

1) A .docx formatted version of the manuscript text (including legends for main figures, EV figures and tables). Please make sure that the changes are highlighted to be clearly visible.

2) Individual production quality figure files as .eps, .tif, .jpg (one file per figure). For guidance, download the 'Figure Guide PDF' (https://www.embopress.org/page/journal/17574684/authorguide#figureformat).

3) A .docx formatted letter INCLUDING the reviewers' reports and your detailed point-by-point responses to their comments. As part of the EMBO Press transparent editorial process, the point-by-point response is part of the Review Process File (RPF), which will be published alongside your paper.

4) A complete author checklist, which you can download from our author guidelines (https://www.embopress.org/page/journal/17574684/authorguide#submissionofrevisions). Please insert information in the checklist that is also reflected in the manuscript. The completed author checklist will also be part of the RPF.

5) Please note that all corresponding authors are required to supply an ORCID ID for their name upon submission of a revised manuscript.

6) It is mandatory to include a 'Data Availability' section after the Materials and Methods. Before submitting your revision, primary datasets produced in this study need to be deposited in an appropriate public database, and the accession numbers and database listed under 'Data Availability'. Please remember to provide a reviewer password if the datasets are not yet public (see https://www.embopress.org/page/journal/17574684/authorguide#dataavailability).

In case you have no data that requires deposition in a public database, please state so in this section. Note that the Data Availability Section is restricted to new primary data that are part of this study.

7) For data quantification: please specify the name of the statistical test used to generate error bars and P values, the number (n) of independent experiments (specify technical or biological replicates) underlying each data point and the test used to calculate p-values in each figure legend. The figure legends should contain a basic description of n, P and the test applied. Graphs must include a description of the bars and the error bars (s.d., s.e.m.).

8) We would also encourage you to include the source data for figure panels that show essential data. Numerical data should be provided as individual .xls or .csv files (including a tab describing the data). For blots or microscopy, uncropped images should be submitted (using a zip archive if multiple images need to be supplied for one panel). Additional information on source data and instruction on how to label the files are available at

.

9) Our journal encourages inclusion of *data citations in the reference list* to directly cite datasets that were re-used and obtained from public databases. Data citations in the article text are distinct from normal bibliographical citations and should directly link to the database records from which the data can be accessed. In the main text, data citations are formatted as follows: "Data ref: Smith et al, 2001" or "Data ref: NCBI Sequence Read Archive PRJNA342805, 2017". In the Reference list, data citations must be labeled with "[DATASET]". A data reference must provide the database name, accession number/identifiers and a resolvable link to the landing page from which the data can be accessed at the end of the reference. Further instructions are available at .

10) We replaced Supplementary Information with Expanded View (EV) Figures and Tables that are collapsible/expandable online. A maximum of 5 EV Figures can be typeset. EV Figures should be cited as 'Figure EV1, Figure EV2" etc... in the text and their respective legends should be included in the main text after the legends of regular figures.

- For the figures that you do NOT wish to display as Expanded View figures, they should be bundled together with their legends in a single PDF file called *Appendix*, which should start with a short Table of Content. Appendix figures should be referred to in the main text as: "Appendix Figure S1, Appendix Figure S2" etc.

- Additional Tables/Datasets should be labeled and referred to as Table EV1, Dataset EV1, etc. Legends have to be provided in a separate tab in case of .xls files. Alternatively, the legend can be supplied as a separate text file (README) and zipped together with the Table/Dataset file.

See detailed instructions here:

.

11) The paper explained: EMBO Molecular Medicine articles are accompanied by a summary of the articles to emphasize the major findings in the paper and their medical implications for the non-specialist reader. Please provide a draft summary of your article highlighting

- the medical issue you are addressing,

- the results obtained and

- their clinical impact.

This may be edited to ensure that readers understand the significance and context of the research. Please refer to any of our

published articles for an example.

12) For more information: There is space at the end of each article to list relevant web links for further consultation by our readers. Could you identify some relevant ones and provide such information as well? Some examples are patient associations, relevant databases, OMIM/proteins/genes links, author's websites, etc...

13) Author contributions: the contribution of every author must be detailed in a separate section (before the acknowledgments).

14) A Conflict of Interest statement should be provided in the main text.

15) Every published paper now includes a 'Synopsis' to further enhance discoverability. Synopses are displayed on the journal webpage and are freely accessible to all readers. They include a short stand first (maximum of 300 characters, including space) as well as 2-5 one-sentences bullet points that summarizes the paper. Please write the bullet points to summarize the key NEW findings. They should be designed to be complementary to the abstract - i.e. not repeat the same text. We encourage inclusion of key acronyms and quantitative information (maximum of 30 words / bullet point). Please use the passive voice. Please attach these in a separate file or send them by email, we will incorporate them accordingly.

Please also suggest a striking image or visual abstract to illustrate your article as a PNG file 550 px wide x 300-800 px high.

EMBO Molecular Medicine has a "scooping protection" policy, whereby similar findings that are published by others during review or revision are not a criterion for rejection. Should you decide to submit a revised version, I do ask that you get in touch after three months if you have not completed it, to update us on the status.

***** Reviewer's comments *****

Referee #1 (Comments on Novelty/Model System for Author):

The models used are adequate for the quetions asked.

Referee #1 (Remarks for Author):

This paper from Nam et al. explores how co-stimulation blockade with CTLA-4-Ig can be supplemented by addition of IL-2 to rescue Treg functions and augmented it's immunosuppresive activity. This raises the possibility of combination therapy for autoimmune and inflammatory diseases, and is thereby builds on the publication of Wang et al, 2022 which is cited by the authors. Mechansistically, the authors studied how IL-2 and CTLA-4 combine to influence Treg using both human and mouse models of Treg differentiation. Although this paper does contain some interesting insights, there are several major issues which should be addressed prior to publication:

1. In vitro experiments focus on the effect of IL-2 and CTLA4-Ig on Treg differentiation cultures driven by addition of TGF-beta. However, in vivo in patients, these treatments will not only effect Treg differentiation, but also the survival and function of thymic Treg cells which is not modeled in their cultures and this is a major limitation of their studies.

2. Throughout the in vitro experiments data on Treg are presented as the % of cells in the culture, but without understanding the impacts of the various treatments on the absolute number of Tregs recovered it is difficult to conclude how these are really impacting Treg abundance. This is a major limitation of their study, and absolute number data is required to properly interpret their results.

3. The mechanistic focus on STAT3-mediated regulation of SMAD2/3 is interesting in the context of Treg differentiation cultures. However, is is not clear is STAT3 phosphorylation is directly mediated by IL-2R signaling (as suggested by Fig 4A), or not (as indicated by Fig 4G). Is it possible that there are other STAT3-activating cytokines present in the cultures such as IL-6 or IL-10 that could be contributing to this? Could these cultures be performed without APCs and with better control over conditions to directly show IL-2R-mediated STAT3 phosphorylation is regulating Treg differentiation?

4. Throughout the manuscript and figure legends, some important details are missing. For instance, disease timepoints for flow cytometric analyses in Figs 1F, 1G are not stated, the significant comparisons denoted by * designations in the right panel of Fig 3B are not stated, etc. Key controls are also sometimes missing, such as the CTLA-4-Ig alone group in Fig 4C.

5. Fig Figs 1I-J, each subpanel of total pateints, responders and non-responders are placed in different scales, and it is therefore difficult to draw direct comparisons between them and therefore these data are hard to interpret.

6. The significance of using RRMS patients samples in Fig 5 is unclear, since no healthy control groups are included in these analyses.

Referee #2 (Remarks for Author):

Summary: In this manuscript, Choi and colleagues explore the impact of CTLA-4 Ig and IL-2 treatment on Treg cells. To conduct their research, the authors utilize (i) in vivo mouse models, including the relapsing-remitting EAE model induced by proteolipid protein (PLP139-151), the MOG35-55-induced EAE model, and wild-type mouse models; (ii) human RNA-seq data from PBMCs of rheumatoid arthritis patients treated with CTLA-4 Ig (Abatacept); and (iii) in vitro T cell stimulation assays using cells isolated from mice and humans (MS patients). The authors report that while CTLA-4 Ig treatment reduces disease activity in the relapsing-remitting EAE model, discontinuation of treatment leads to a more severe relapse compared to untreated controls. This exacerbation may be linked to a reduction in $CD4^+$ $Foxp3^+$ Treg cells. To determine if a similar phenomenon occurs in humans, the authors analysed gene expression profiles of PBMCs from rheumatoid arthritis (RA) patients treated with Abatacept. Notably, non-responders exhibited decreased expression of Treg-associated genes. Further, in vitro experiments reported that antigen-specific stimulation of naïve 2D2 transgenic T cells in the presence of TGF-β, CTLA-4 Ig, and IL-2 led to more efficient differentiation into $CD25^+$ $Foxp3^+$ Treg cells, indicating a potential synergistic effect of CTLA-4 Ig and IL-2 in promoting Treg development. In the MOG35-55-induced EAE model, CTLA-4 Ig treatment significantly alleviated disease severity. Notably, co-administration of IL-2 further improved disease control, likely by mitigating the reduction in Treg cells associated with CTLA-4 Ig therapy. Finally, the study suggested a distinct mechanism by which CTLA-4 Ig modulated IL-2 signalling, by selectively dampening STAT3 activation without disrupting STAT5 pathways. When combined with IL-2, this selective signalling environment favoured the differentiation of effector Treg cells, driven by enhanced CD25/STAT5 and TGF-β/Smad3 activity. These insights suggest a novel therapeutic approach in which IL-2 and CTLA-4 Ig act synergistically to suppress pathogenic effector T cells while promoting regulatory T cell function, offering a refined strategy for treating autoimmune conditions.

Major comments:

1) Need for broader immune cell profiling
While the authors report a more severe disease relapse following cessation of CTLA-4 Ig therapy, it remains unclear why this occurs, particularly given the observed reduction in IL-17-producing $CD4^+$ T cells (Fig. 1F) and the apparent maintenance of a favourable Treg-to-effector ratio (Fig. 1G). This raises questions about other immunological mechanisms that may contribute to disease exacerbation. The current analysis is limited to T cell subsets, and the study would benefit from a broader investigation of immune components involved in disease progression. In particular, assessing the activation status of antigen-presenting cells (APCs)-for instance, by evaluating CD80/CD86 expression-could provide valuable insights. Additionally, profiling other proinflammatory cytokines such as IFN-γ and IL-6 may help clarify the underlying mechanisms driving disease severity after treatment withdrawal.
These aspects should also be addressed in the RNA-seq data of the RA patient.
Moreover, the results in Figure 2 indicate that Treg cell differentiation is increasing while effector T cell differentiation is suppressed. Unlike the authors' statements, such an experimental setup in Figure 2 is not designed to measure effector T cell activation. Therefore, the authors need to revisit their conclusions and correct their statements on T cell activation.

2) "Synergistic effect" needs further investigation
In order to conclude a synergistic effect of two compounds, in this case CTLA-4 Ig and IL-2, the effects of either treatment as a monotherapy should be tested as well. The treatment efficacy of IL-2 alone is for example missing in Figure 3 and therefore a claim on synergism is not possible. Particularly, the presented results in Figure 3H indicates that IL-2 does not significantly restore eTreg diminished by CTLA-4 as claimed in the figure title. On a side note, the injected amount of IL-2 (20 ng per injection) seems far too low, even for "low-dose IL-2" in mouse experiments (expected "low-dose IL-2" treatments would usually contain 1-5 microgram of IL-2 per injection)

3) Need for in vivo validation of CTLA-4 Ig-mediated modulation of IL-2 signaling in EAE
The manuscript would benefit from a more thorough investigation of the IL-2-driven signalling pathway in the in vivo setting. It remains unclear whether CTLA-4 Ig can inhibit IL-2-induced STAT3 phosphorylation, which could potentially shift the balance toward enhanced TGF-β/Smad2/3 signalling and increased Foxp3 expression, particularly in the EAE model. To support the proposed mechanism involving modulation of PI3K/STAT3 signalling by CTLA-4 Ig, the authors should extend their analysis beyond the in vitro findings presented in Figure 4. Specifically, ex vivo analysis of pSTAT3 and pSmad2/3 levels in $CD4^+$ T cells

isolated from EAE mice would be critical to validate the proposed pathway and strengthen the translational relevance of combining CTLA-4 Ig with IL-2 as a therapeutic strategy.

Minor Comments:

P.4 Please add a reference for the following sentence: "However, its expansion to other autoimmune diseases has still been largely limited in clinical trials, primarily due to its unintended effects on Treg cell depletion."
P.16: "In this study, PBBM's from RRMS..." - Please correct "PBBM's" to "PBMC's"
P.22: According to the Materials section on page 22, the study used the following setup "Cells were cotreated with 5 μM of dNP2-ctCTLA-4 or 0.5 μM of CTLA-4 Ig." Is this correct? If so, please indicate this tratment in the Figures.

P.25: According to the Materials section on page 25, the study used the following setup for the relapsing-remitting EAE mouse model: "100 μg of dNP2-ctCTLA-4 and 100 μg of CTLA-4 Ig (Orencia, BMS) was injected intraperitoneally daily from day 7 to day 20 during early phase."
Is this correct? If so, please indicate this tratment in the Figures.

Referee #3 (Remarks for Author):

In this work, Nam and colleagues present a series of experiments that explore the mechanism through which treatment with CTLA4.Ig might decrease the abundance of regulatory T cells. In general, I found the manuscript difficult to read and the flow of experiments difficult to follow. They interchangeably use different mouse models, observations from patients, and in vivo activation of murine and human cells and draw parallels that are not obvious to prove their points. The experiments lack an adequate sample size and, in some cases, important experimental controls. I do not feel the authors are able to adequately prove any of their claims.
Specific comments:
1. The manuscript is written in a very confusing manner. Professional English editing may be desirable.
2. Figure 1. CTLA4.Ig reduces Treg numbers by reducing CD28 signaling. The main point of the figure is that though CTLA4.Ig may decrease effector T cell activation, through the same mechanism it causes a drop in Treg abundance that may be counterproductive in the setting of autoimmune diseases. The authors provide two sets of data to prove their point: (1) EAE in mice treated with CTLA4.Ig or control, and (2) an analysis of transcriptomic data from patients with rheumatoid arthritis treated with abatacept. However, the data in mice is underpowered and not robust and the transcriptomic analysis fails to demonstrate their claim. Specifically:
a. How many times was the experiment depicted in Fig. 1B to D repeated? The figure legend says 5 animals per group and only 5 dots are depicted. In my opinion, the experiment should be repeated with more mice, to determine whether there are differences between the groups.
b. Fig 1B has very large error bars and I doubt there are any statistical differences in Fig 1B after day 20. The same applies to Fig1C, where the differences do not seem statistically significant.
c. Fig. 1E to G. When where those analyses performed? I assume they represent the same mice from Fig. 1B. It would be important to analyze CNS infiltration at earlier time points, after CTLA4.Ig treatment and before/during relapse.
d. Fig. 1F. Percentage of IL-17A+ CD4 T cells should be calculated as such (% of CD4 T cells that produce IL-17). The way it was calculated (% of IL-17+ CD4+) is misleading, because it is affected by the proportion of CD4- cells. The same applies to Fig. 1G.
e. The transcriptomic analysis performed with published data from patients with rheumatoid arthritis fails to yield any significant data. If the authors wish to evaluate the effects of abatacept in Treg numbers and/or function, it would have been more appropriate to evaluate Tregs using flow cytometry. For example, based on lower CD28 transcription, the authors claim that CD28 expression may be a factor involved in response to abatacept. However, they ignore if CD28 is differentially expressed and ignore the pattern of expression of this receptor in Tregs and other T cells. Then, they identify genes that encode signaling molecules as important and they propose this is an effect of "attenuation of costimulation". In my opinion, these claims should be supported by data in cells at the protein level.
3. Figure 2. CTLA4.Ig boosts Treg differentiation in the presence of IL-2. This is not connected with the concept presented in the previous figure. Treg differentiation from naïve CD4 T cells is a different process than Treg maintenance in the setting of a systemic inflammatory disease. It has been proven before (Sakaguchi PNAS) that lack of CD28 increases Treg differentiation.
4. Figure 3. In vivo administration of IL-2 prevents loss of Tregs induced by CTLA4.Ig. This experiment is very important to prove the main message of the paper. However, the authors used a different mouse strain than in Figure 1... in Figure 1, the main effects of CTLA4.Ig (and allegedly of Treg depletion) was more severe and common relapses, but in this figure, they chose a strain with a non-relapsing disease. As in Figure 1, the mouse numbers seem very small and in some parameters the data shows a large dispersion. Importantly, there is no group with IL-2 and no CTLA4.Ig and the experimental design does not prove

that IL-2 is restoring Treg numbers and phenotype by a specific mechanism.
5. Figure 4 is very relevant as it shows a possible mechanism. However, it is not completely explored, and no data demonstrates that this mechanism in place in the in vivo models presented. Specifically:
a. The blot presented in Fig. 4A is very impressive, but the cumulative data does not look so strong, which makes me wonder if the blot is representative. In the pSTAT3 bar graph of Fig. 4B, is there a significant difference between PBS and IL-2 (first and third bars)? If not, it would be hard to sustain that IL-2 is really inducing STAT3 phosphorylation in a significant manner. Additionally, it would be more appropriate to use STAT3 as a loading control (instead of b-actin).
b. The authors claim that CTLA4.Ig decreases IL-2-induced STAT3 phosphorylation by inhibiting PI3K. However, they do not actually show that CTLA4.Ig inhibits PI3K. It would be important to show that.
c. In Fig. 4G, the two pSTAT3 blots are discordant... is there a mistake?
6. Figure 5 is also hard to interpret. I do not think it proves any point.

Referee #1 (Remarks for Author):

This paper from Nam et al. explores how co-stimulation blockade with CTLA-4-Ig can be supplemented by addition of IL-2 to rescue Treg functions and augmented it's immunosuppresive activity. This raises the possibility of combination therapy for autoimmune and inflammatory diseases, and is thereby builds on the publication of Wang et al, 2022 which is cited by the authors. Mechansistically, the authors studied how IL-2 and CTLA-4 combine to influence Treg using both human and mouse models of Treg differentiation. Although this paper does contain some interesting insights, there are several major issues which should be addressed prior to publication:

[Answer]

We thank the reviewer for the careful evaluation and constructive summary of our manuscript. We appreciate the recognition of the potential relevance of combining costimulation blockade by CTLA-4 Ig with a suboptimal amount of IL-2 to increase Treg differentiation and immunosuppressive activity, as well as the mechanistic insight into the IL-2 → pSTAT3 ⊣ pSMAD2/3 axis, which represents a major distinction from the recent paper published by Wang et al., 2022. We have carefully addressed the major concerns raised and revised the manuscript, accordingly, as detailed below.

[Comment 1]

In vitro experiments focus on the effect of IL-2 and CTLA4-Ig on Treg differentiation cultures driven by addition of TGF-beta. However, in vivo in patients, these treatments will not only effect Treg differentiation, but also the survival and function of thymic Treg cells which is not modeled in their cultures and this is a major limitation of their studies.

[Response 1]

We thank the reviewer for this insightful comment. In the current manuscript, our in vitro experiments were designed to test whether blockade of costimulatory signaling by CTLA-4 Ig, in the presence of IL-2, enhances Treg induction under TGF-β–driven conditions. We agree that this culture system does not fully recapitulate

thymic Treg (nTreg) biology or the maintenance of pre-existing Treg populations in vivo.

As shown in Fig. EV1 of original manuscript, CTLA-4 Ig (400 µg/mouse, i.p., administered every other day for three injections) resulted in a significant reduction in the proportion of $Foxp3^{+}$ in CD4 T cells one week after treatment, indicating that tonic costimulation would be required for Treg maintenance or survival (Immunol Rev. 2009 May;229(1):41-66., Immunity. 2000 Apr;12(4):431-40.).

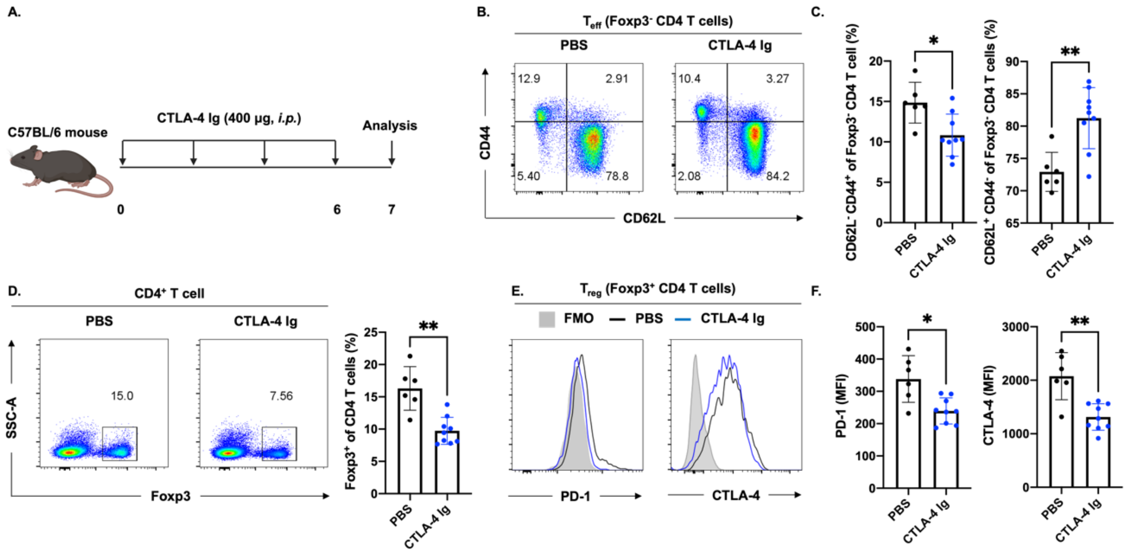

Fig. EV1A-F in original manuscript

Importantly, a similar phenomenon has been observed in the human setting. In rheumatoid arthritis patients treated with Abatacept for 3 months, $Foxp3^{+}$ Treg cells, $Helios^{+}$ T cells and $CD39^{+}$ T cells were significantly diminished in PBMCs (BMC Immunol. 2013 Aug 5;14:34.), which is also consistent with Fig 1 of RNA-seq analysis (Fig 1I and J), suggesting that costimulation blockade by CTLA-4 Ig might impair functional Treg cell maintenance (Rheumatology (Oxford). 2022 Mar 2;61(3):1233-1242.).

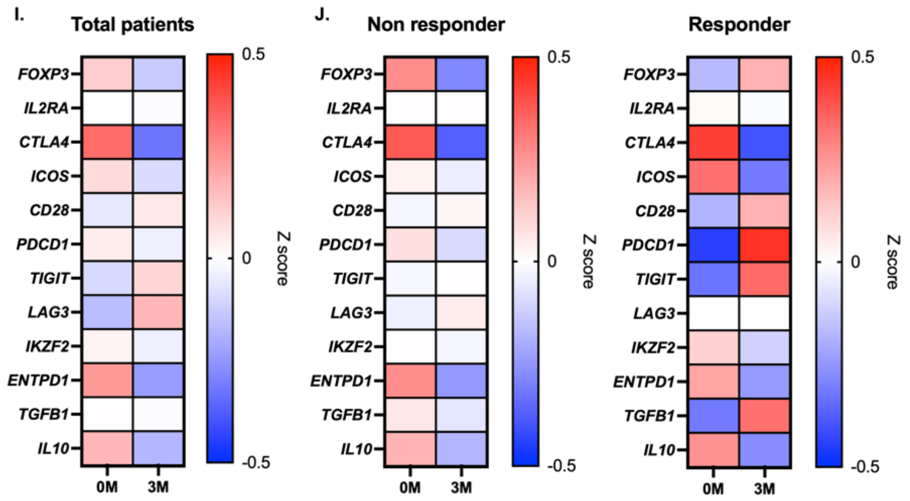

Fig 1I and J in revised manuscript. (I) Treg-related gene expression comparison between before and after Abatacept treatment for 3 months in total patients (I) and comparison between non-responders and responders (J) using heatmap.

There are several previous reports demonstrating that CD28 and IL-2 signaling are essential for maintaining Treg stability (Immunity. 2000 Apr;12(4):431–40; Immunity. 2024 Mar 12;57(3):414–428). We performed additional experiments using purified Foxp3$^+$ CD4$^+$ T cells stimulated with anti-CD3 or anti-CD3/CD28 antibodies, with or without IL-2, for 3 days. These experiments confirmed that IL-2 and costimulatory signaling are beneficial for increasing the expression of Foxp3, CD25, CTLA-4, and Ki67, indicating that both IL-2 and costimulatory signaling are essential not only for Treg maintenance but also for survival (Figure R1).

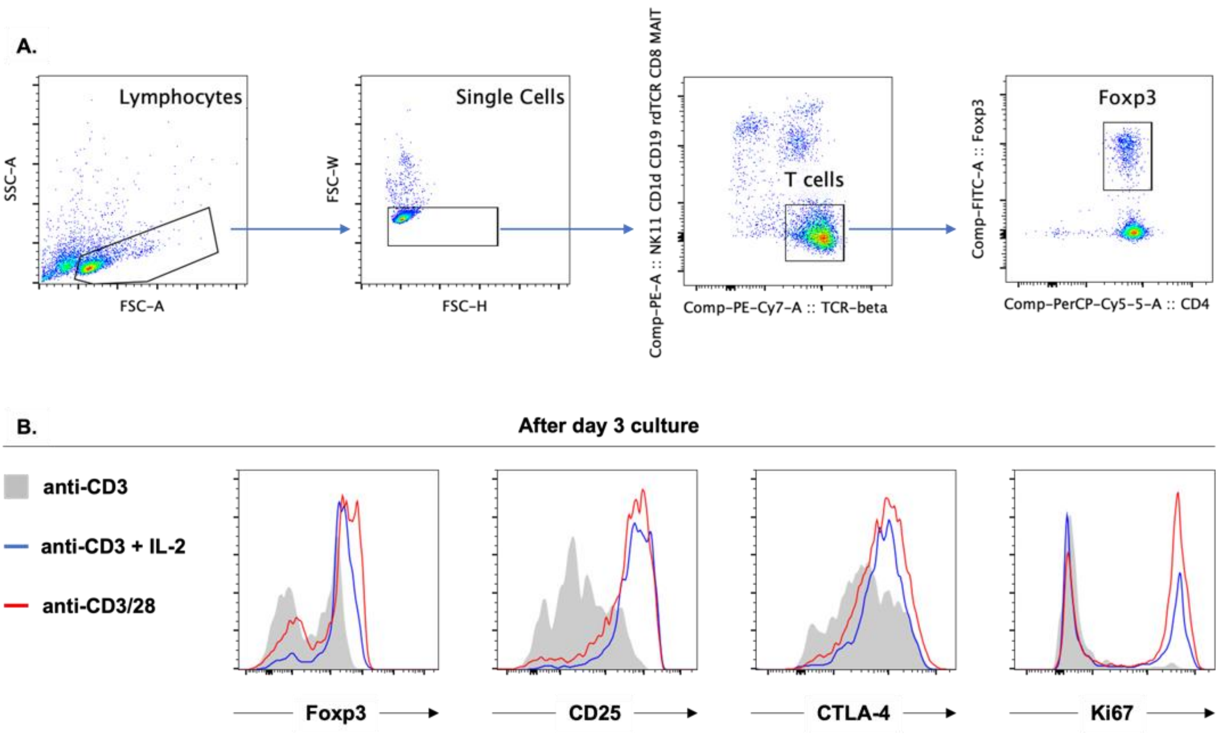

Figure R1. IL-2 compensates for loss of CD28 costimulation in purified Foxp3$^+$ Treg cells.
(A) Gating strategy for Foxp3$^+$ CD4 T cells. (B) FACS-sorted Foxp3$^+$ CD4 T cells were cultured with anti-CD3 (5 μg/ml) with or without IL-2 (50 U/ml), or with anti-CD3 and anti-CD28 (5 μg/ml), for 3 days. Representative histogram of Foxp3, CD25, CTLA-4 and Ki-67 expression after culture for 3 days.

Additionally, we examined thymic Treg cell markers, including Foxp3, PD-1, CTLA-4, and Helios. Consistent with the reduction observed in peripheral Treg populations, the frequency of Foxp3$^+$ CD4 T cells in the thymus was significantly decreased suggesting that costimulatory signaling may contribute to thymic Treg generation (Fig. EV2A). However, the expression of suppressive molecules, including PD-1 and CTLA-4, as well as the lineage-associated marker Helios, remained unchanged within the thymic Foxp3$^+$ Treg compartment (Fig. EV2B and C).

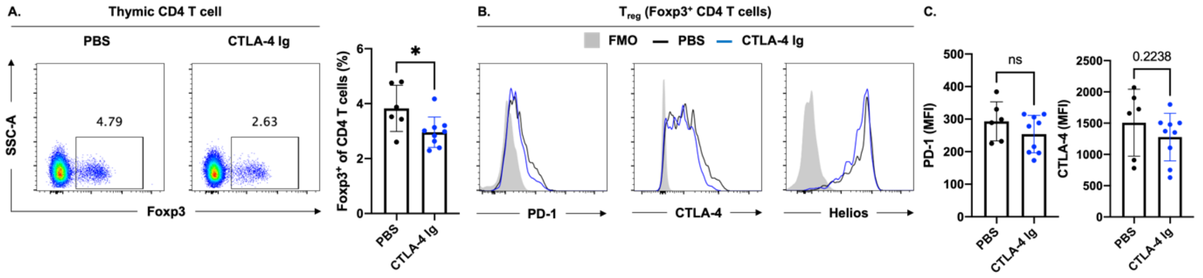

Revised Fig. EV2. Costimulation blockade by CTLA-4 Ig reduced thymic Treg cells in steady-state mice.

(A) Expression of Foxp3 in CD4 T cells from thymus was analysis by flow cytometry. (B) Representative histogram of PD-1, CTLA-4 and Helios in thymic Foxp3$^+$ CD4 T cells. (C) Bar graph of MFI of PD-1, CTLA-4.

Therefore, CTLA-4 Ig and IL-2 would not only promote the differentiation of naïve CD4 T cells into Treg cells but may also influence thymic-derived or pre-existing Treg cells by modulating their Foxp3 and CD25 expression. The therapeutic effects observed in the EAE model in our study likely reflect the combined impact of increased inducible Treg generation during antigen stimulation in the draining lymph nodes together with the maintenance of Treg stability and functionality.

However, the IL-2 dose (20 ng per mouse) was carefully selected based on our prior titration experiments in the EAE model. Although this relatively low dose of IL-2 alone did not significantly expand Treg cells in vivo, co-treatment with CTLA-4 Ig restored the Treg proportion. This finding indicates that IL-2 monotherapy exerts only a minimal effect on Treg expansion, whereas it preferentially enhances Treg differentiation under conditions of costimulatory blockade. Therefore, our experimental design was intended to evaluate IL-2–mediated Treg differentiation in the presence of CTLA-4 Ig rather than to achieve maximal Treg expansion.

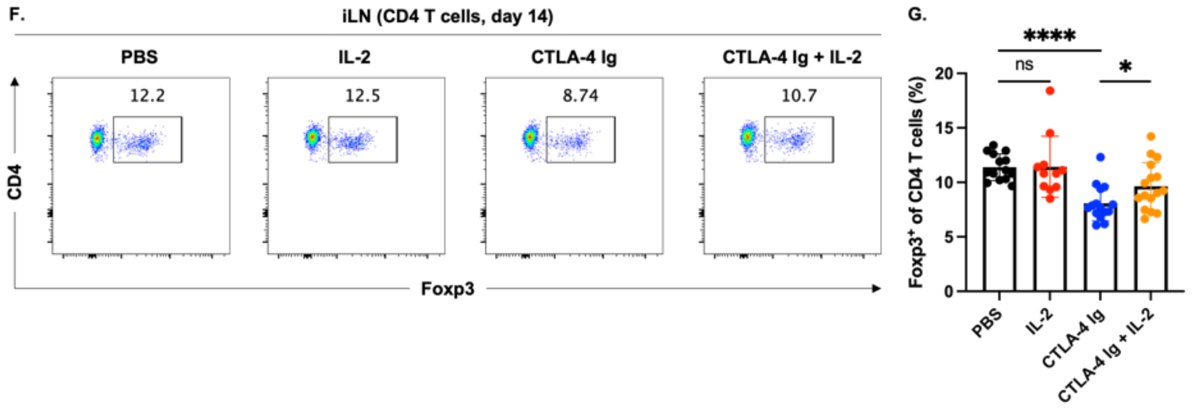

Revised Fig 3F and G. Representative FACS dot plot (F) and bar graph (G) showing Foxp3 expression in CD4 T cells from the iLN of EAE mice.

We have added Fig. EV2 to the revised manuscript and described these data in the revised Results section (line 129-132). We have denoted that 20 ng of IL-2 does not affect on Treg expansion in Result section (line 217-218). In addition, we have added corresponding sentences and references indicating costimulatory signaling and IL-2

signal are required for Treg maintenance and differentiation in Discussion section (line 391-412).

[Comment 2]

Throughout the in vitro experiments data on Treg are presented as the % of cells in the culture, but without understanding the impacts of the various treatments on the absolute number of Tregs recovered it is difficult to conclude how these are really impacting Treg abundance. This is a major limitation of their study, and absolute number data is required to properly interpret their results.

[Response 2]

We thank the reviewer for this important comment. We agree that presenting Treg data solely as percentages has inherent limitations, and that absolute cell numbers would provide the most definitive assessment of Treg abundance.

Although the percentage of $Foxp3^+$ indicates their proportion within the CD4 T cell population, to fully address this issue, it is beneficial to assess absolute cell numbers by factoring in total live cells and the frequency of CD4 expression.

For flow cytometric analysis, a fixed number of 10,000 live CD4 T cell events were acquired for each sample to ensure statistical comparability. To determine Treg abundance (absolute cell numbers), the values were calculated by normalizing the frequencies against the total live cell and CD4 percentages.

In revised manuscript, we add gating strategies for in vitro to calculate total Treg frequency. Under Th0 condition driven by MOG_{35-55} antigen and APCs in presence of TGF-β, treatment with CTLA-4 Ig markedly reduced the proportion of CD4 T cells as well as frequency of live, even in the presence of IL-2.

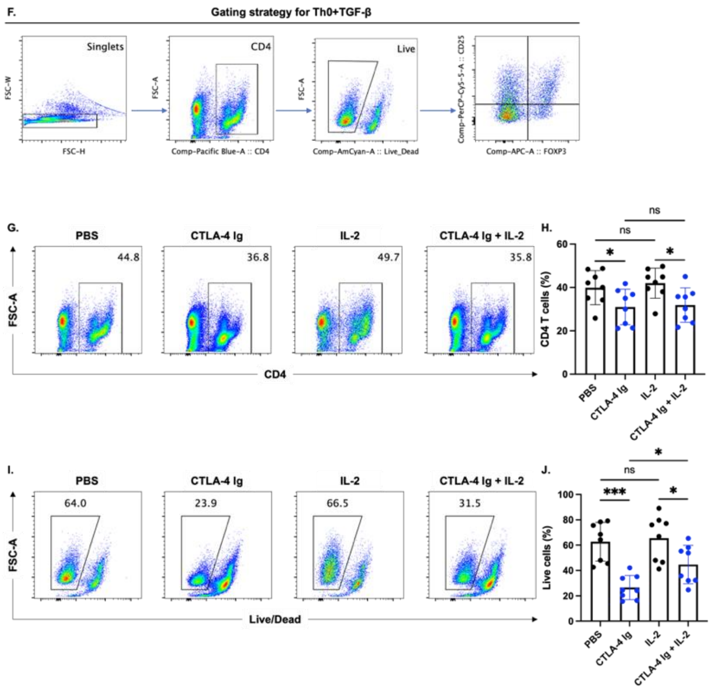

Revised Appendix Fig. 1F-J. Reduction of CD4^{+} T cells and cell viability under Th0+TGF-β condition. (F) Gating strategy for Th0+TGF-β culture condition using 2D2/APC co-culture. (G) Representative FACS dot plot and (H) quantification of the proportion of CD4^{+} T cells. (I) Representative FACS dot plot and (J) quantification of live cells within the CD4^{+} T cell population.

Within the live CD4 T cell gate, CTLA-4 Ig alone appeared to increase the proportion of CD25^{+} Foxp3^{+} Treg cells. However, when reanalyzed as total frequency (absolute abundance) across the entire gating strategy, the Treg population was significantly reduced, as shown in the following figures. IL-2 alone increased both the relative proportion and absolute number of Treg cells. In contrast, although the combination of CTLA-4 Ig and IL-2 increased the proportion of Treg cells within CD4 T cell gate compared with IL-2 alone, CTLA-4 Ig mediated reduction of total proportion of CD4 T cells and cell viability offset this effect, resulting in a total Treg frequency comparable to that of the IL-2 only group.

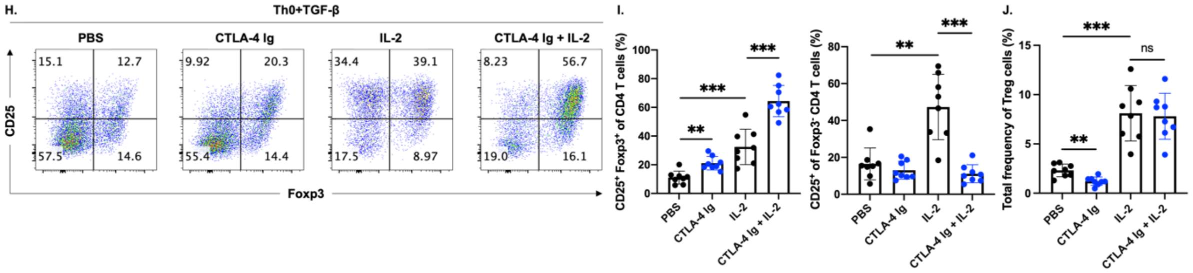

Revised Fig 2H-J. Proportion of CD25^{+} in Foxp3^{+} and Foxp3^{-} in CD4 T cells and total frequency of Treg cells.

(H) Representative FACS dot plot showing $CD25^{+}$ $Foxp3^{+}$ proportion in CD4 T cells cultured with IL-2, with or without CTLA-4 Ig in Th0+TGF-β condition. (I) Bar graph indicating $CD25^{+}$ $Foxp3^{+}$ Treg population and $CD25^{+}$ $Foxp3^{-}$ effector T cell population. (J) Total frequency of $CD25^{+}$ $Foxp3^{+}$ Treg cells.

We performed additional experiments using the $MOG_{35\text{-}55}$ antigen and APC stimulation system under Th17-polarizing conditions (IL-6 + TGF-β). The CTLA-4 Ig treatment group exhibited a pattern similar to that observed in Th0+TGF-β cultures, characterized by reduced live cell numbers and a slight increase in the frequency of $Foxp3^{+}$ cells. Notably, however, the combination of CTLA-4 Ig and IL-2 demonstrated a clear synergistic effect: both the proportion of $Foxp3^{+}$ cells within the CD4 population and the total frequency (absolute abundance) of Treg cells were significantly increased compared to either the CTLA-4 Ig or IL-2 single-treatment groups.

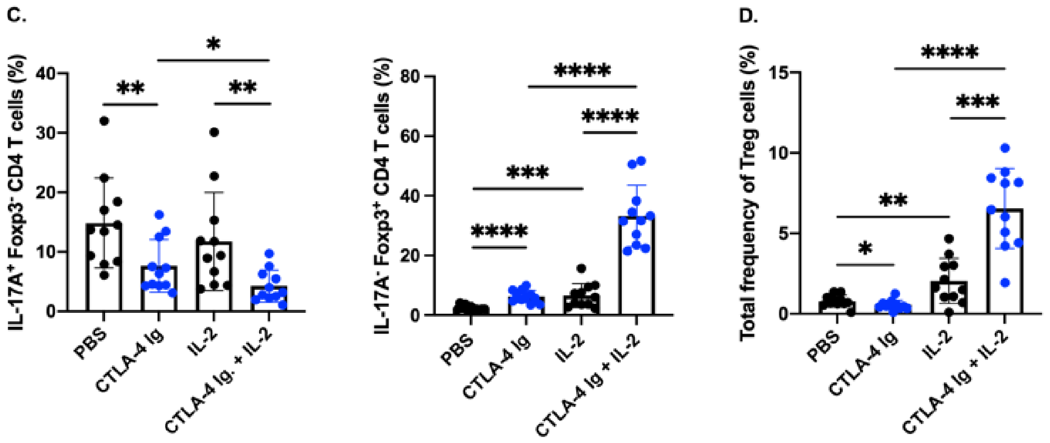

Revised Fig 2C and D. (C) Bar graph of $IL\text{-}17A^{+}$ $Foxp3^{-}$ effector CD4 T cells and $IL\text{-}17A^{-}$ $Foxp3^{+}$ iTreg cells. (D) Total frequency of $IL\text{-}17A^{-}$ $Foxp3^{+}$ iTreg cells.

This difference can be explained by changes in CD4 T cell viability under Th17-polarizing conditions. Although CTLA-4 Ig reduced the total number of CD4 T cells even in the presence of IL-2 compared with IL-2 alone, the proportion of live CD4 T cells was restored under Th17 conditions to a level comparable to that observed in the IL-2–only group, and notably higher than that observed under Th0+TGF-β conditions. As a result, the changes in the total frequency (absolute abundance) of $Foxp3^{+}$ Treg cells within the CD4 T cell compartment closely mirrored the pattern observed for the proportion of $Foxp3^{+}$ cells.

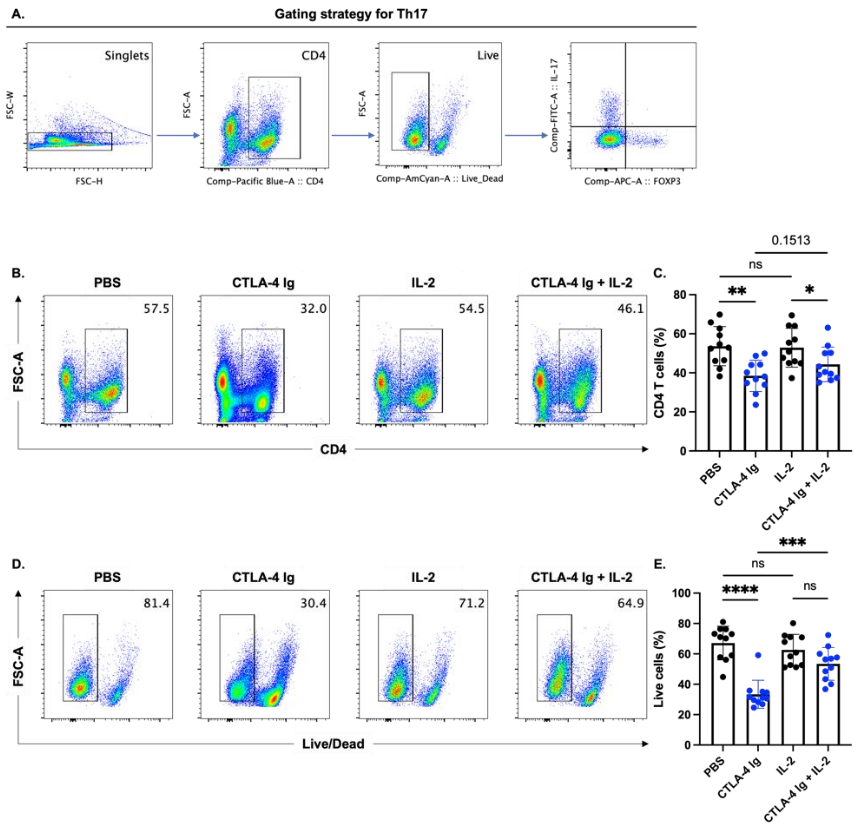

Revised Appendix Fig. 1A-E. Effects of CTLA-4 Ig and IL-2 on CD4^{+} T cell frequency and cell viability under Th17-polarizing condition.

(A) Gating strategy for culture Th17-polarizing condition using 2D2/APC co-culture. (B) Representative FACS dot plot and (C) quantification of the proportion of CD4 T cells. (D) Representative FACS dot plot and (E) quantification of live cells within the CD4 T cell population.

In the revised manuscript, we have added the total frequency of Treg cells (absolute number or abundance) in Fig. 2D (Th17) and Fig. 2J (Treg), along with the corresponding text in the Results section (Th17: line 174–188; Treg: line 188–197). The gating strategy for the in vitro co-culture experiments shown in Fig. 2, as well as the proportion of CD4 T cells and cell viability under both conditions, have been included in Appendix Fig. 2.

[Comment 3]

The mechanistic focus on STAT3-mediated regulation of SMAD2/3 is interesting in the context of Treg differentiation cultures. However, is is not clear is STAT3 phosphorylation is directly mediated by IL-2R signaling (as suggested by Fig 4A), or not (as indicated by Fig 4G). Is it possible that there are other STAT3-activating cytokines present in the cultures such as IL-6 or IL-10 that could be contributing to this? Could these cultures be performed without APCs and with better control over conditions to directly show IL-2R-mediated STAT3 phosphorylation is regulating Treg differentiation?

[Response 3]

We thank to the reviewer for this clarifying comment on the interpretation of STAT3 activation by IL-2 in our Treg differentiation experiments.

First of all, we apologize for the confusion regarding Figure 4G, which shows a strong pSTAT3 signal in solenocytes stimulated with anti-CD3 antibody and TGF-β for 3 days. These experiments were repeated multiple independent times, during which some variability in the baseline pSTAT3 signal was observed in the control group, ranging from relatively strong to weak levels. However, the increase in pSTAT3 following IL-2 treatment in addition to anti-CD3 antibody and TGF-β was consistent across most experiments. We inadvertently selected a representative image from the single replicate that showed a weaker (marginal) response by IL-2, as the base line control signal in that replicate was too strong.

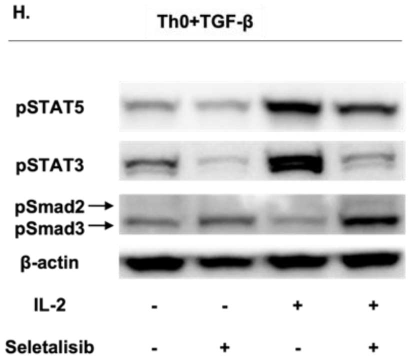

Fig 4H. Replaced pSTAT3 blotting in revised Fig. 4H (Fig. 4G in the original manuscript).

In the revised version of manuscript, we have replaced representative image that reflects the consistent results from the repeated experiments.

The reason we designed the in vitro experiments as an APC–T cell co-culture system is that B7 molecules expressed on APCs are the binding targets of CTLA-4 Ig. As suggested by the reviewer, we performed additional experiments using FACS-sorted naïve CD4^{+} T cells stimulated with anti-CD3 or anti-CD3/CD28 antibodies to mimic the effects of CTLA-4 Ig in the absence of APCs.

Stimulation of FACS-sorted naïve CD4^{+} T cells for 3 days with anti-CD3 antibody alone had minimal effects on pSTAT5 and induced only a slight increase in pSTAT3. In contrast, stimulation with anti-CD3 plus anti-CD28 antibodies robustly induced both pSTAT5 and pSTAT3, indicating that STAT3 activation is dependent on costimulatory signaling. Importantly, the addition of IL-2 further enhanced STAT3 phosphorylation under anti-CD3/anti-CD28 stimulation (Revised Fig 4D), whereas neutralization of IL-2 markedly reduced both pSTAT3 and pSTAT5 levels (Revised Fig. EV7A).

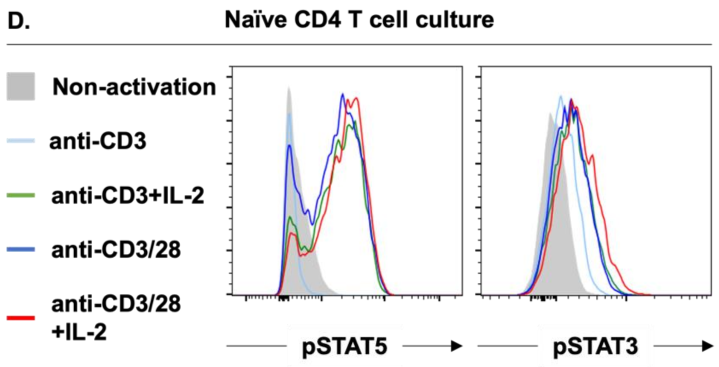

Revised Fig 4D. The expression level of pSTAT5 and pSTAT3 in CD4 T cells cultured with anti-CD3 (2 μg/ml), anti-CD3 + anti-CD28 (5 μg/ml) with or without 50 U/ml of IL-2 for 3 days.

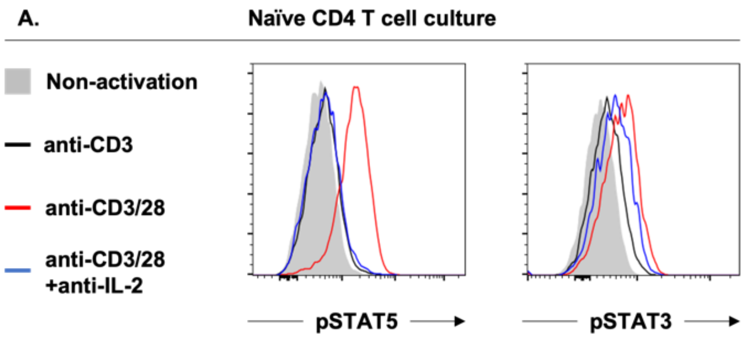

Revised Fig. EV7A. The expression level of pSTAT5 and pSTAT3 in CD4 T cells cultured with anti-CD3 (2 µg/ml) anti-CD3 + anti-CD28 (5 µg/ml) or anti-CD3 + anti-CD28 with anti-IL-2 neutralizing antibody (5 µg/ml) for 3 days.

Accordingly, we confirmed beneficial effects for Treg differentiation by anti-CD3 antibody with IL-2 treatment which showed lower pSTAT3 level than with CD28 antibody and IL-2 as shown in the above Fig. 4D. Without anti-CD28 stimulation, IL-2 treatment with TGF-β in naïve CD4 T cells for 3 days showed significantly increased CD25$^+$ Foxp3$^+$ regulatory T cells with increased Foxp3 intensity (revised Fig. EV5).

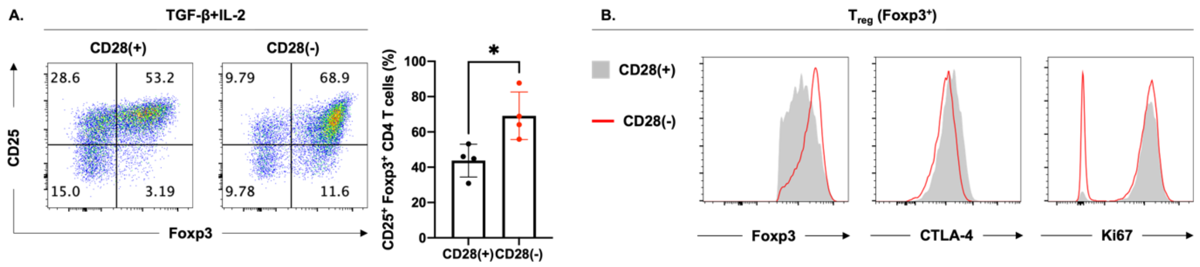

Revised Fig. EV5. IL-2 promotes selective Treg induction under low-costimulation conditions.

(A, B) FACS sorted naïve CD4 T cells (CD4$^+$ CD25$^-$ CD62L$^+$ CD44$^-$) were stimulated with anti-CD3 (5 ug/ml) with or not anti-CD28 (5 µg/ml) in presence of TGF-β (2 ng/ml) with or not IL-2 (50 U/ml) for 3 days. (A) Representative FACS dot plot of Foxp3$^+$ CD25$^+$ CD4 T cells and (B) bar graph.

Additionally, to more evaluate IL-2–mediated signaling, we analyzed Foxp3$^+$ regulatory CD4$^+$ T cells, which constitutively express CD25, in both in vitro and in vivo settings. In splenocytes, IL-2 treatment for 4 hours robustly induced pSTAT5 but did not significantly increase pSTAT3, whereas IL-6 selectively enhanced pSTAT3. Notably, co-stimulation with IL-2 and anti-CD3 elevated pSTAT3 to levels comparable to those induced by IL-6 (Revised Fig. EV8B). These results indicate that IL-2–mediated STAT3 activation requires concomitant costimulatory signaling rather than IL-2 stimulation alone.

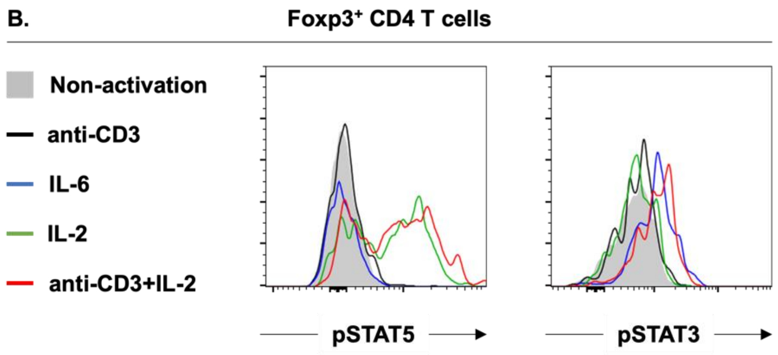

Fig. EV7B. STAT3 phosphorylation by IL-2 requires T cell receptor stimulation.

(B) Isolated splenocyte stimulated by IL-2 (50 U/ml) or IL-6 (30 ng/ml) 4 hours with or without anti-CD3 (5 μg/ml). The expression levels of pSTAT5 and pSTAT3 in Foxp3$^+$ CD4 T cells.

Collectively, we confirmed STAT3 phosphorylation can be induced by IL-2 in the presence of TCR stimulation, regardless of the presence of APCs.

In the revised manuscript, we have revised Fig. 4D and added Fig. EV7, along with the corresponding text in the Results (line 247–254; 254-257) and Discussion (line 457–462). In addition, we have also updated Fig. EV5 with additional repeat experiments.

[Comment 4]

Throughout the manuscript and figure legends, some important details are missing. For instance, disease timepoints for flow cytometric analyses in Figs 1F, 1G are not stated, the significant comparisons denoted by * designations in the right panel of Fig 3B are not stated, etc. Key controls are also sometimes missing, such as the CTLA-4-Ig alone group in Fig 4C.

[Response 4-1]

We thank the reviewer for pointing out these missing details and controls. We have carefully revised the manuscript and figure legends to address these issues.

For the SJL EAE dataset shown in Fig 1F and 1G, we have now clearly the indicated analyzed disease time points at which flow cytometric analyses in the corresponding figure panels and legends.

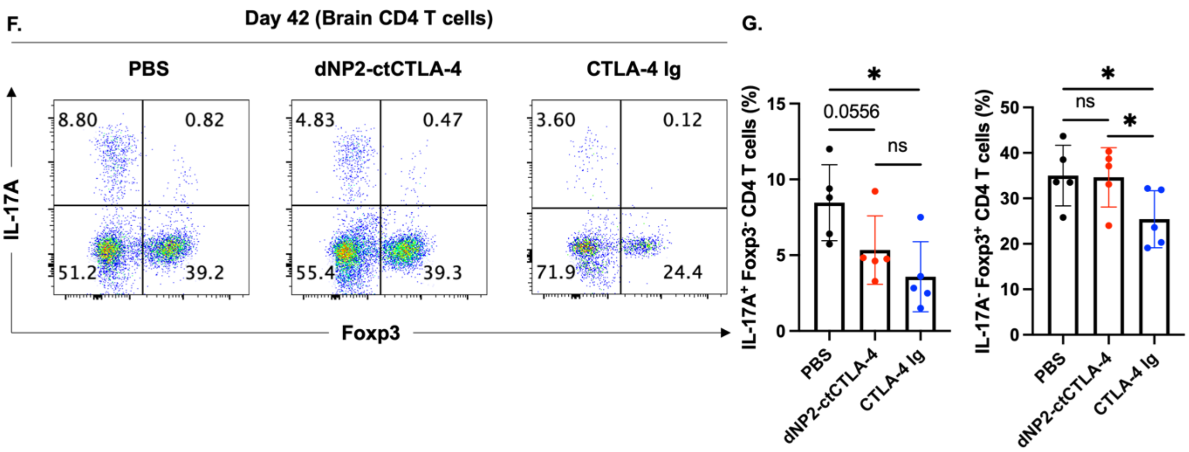

Revised Fig 1F and G indicating disease time points for flow cytometric analysis.

[Response 4-2]

Regarding the regular EAE dataset of Fig 3, we acknowledge that the IL-2–only group was not included in the previous version of the manuscript. In the original experiments, although both the CTLA-4 Ig–treated group and the CTLA-4 Ig+IL-2 co-treatment group showed significant therapeutic effects compared with the control group, no statistically significant difference was observed between these two treatment groups.

To address this limitation, we performed additional experiments including an IL-2–only treatment group. In addition, we have now included statistical comparisons and significance annotations between experimental groups to clearly indicate intergroup differences in revised Fig 3B (total four independent experiments).

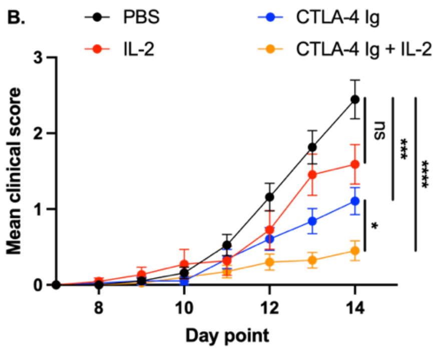

Revised Fig 3B. Clinical disease score after EAE induction.

[Response 4-3]

In addition, the reviewer noted the absence of a CTLA-4 Ig–alone group in Fig. 4C. In the original figure, we focused on conditions in the presence of IL-2 to facilitate visual comparison of signaling changes, as inclusion of all conditions in a single histogram reduced clarity. To address this concern, we have now included the CTLA-4 Ig–alone condition (in the absence of IL-2) in revised Fig. 4C.

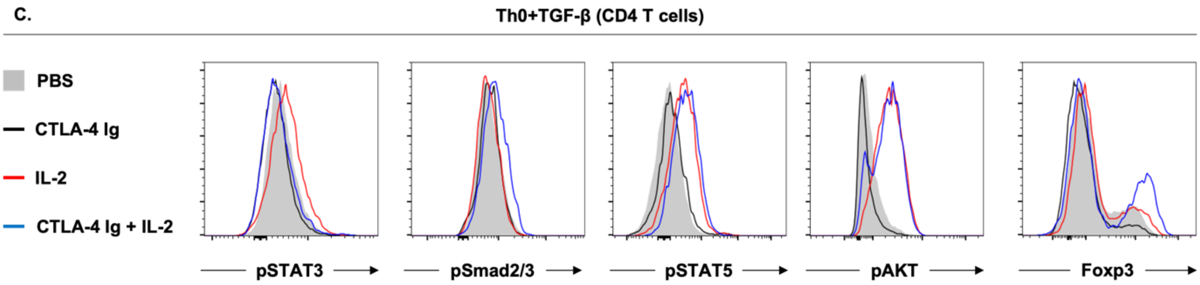

Revised Fig 4C. Phosphorylation of STAT3, Smad2/3 C-term, STAT3 and AKT with expression of Foxp3 in CD4 T cells.

[Comment 5]

Fig Figs 1I-J, each subpanel of total pateints, responders and non-responders are placed in different scales, and it is therefore difficult to draw direct comparisons between them and therefore these data are hard to interpret.

[Response 5]

We thank the reviewer for this helpful comment regarding data presentation. In response, we have unified the y-axis scales across all subpanels in Fig 1I-K to facilitate direct comparison among total patients, responders, and non-responders. Following rescaling, we confirmed that the overall expression patterns remained consistent with those presented in the original manuscript.

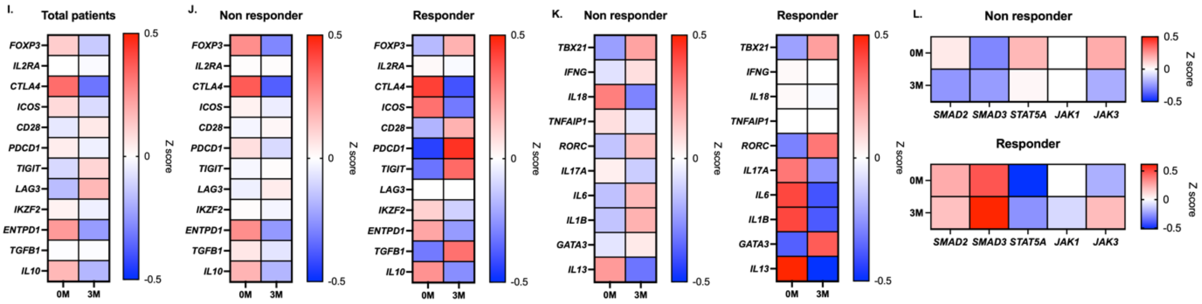

Revised Fig 1I-L with rescaling. (I) Treg-related gene expression comparison between before and after Abatacept treatment for 3 months in total patients using heatmap. (J) Treg-related gene expression comparison between non-responders and responders using heatmap (K) Inflammatory cytokine gene expression comparison utilizing heat map between non-responders and responders. (L) Comparison of *SMAD2*, *SMAD3, STAT5A, JAK1* and *JAK3* gene expression between non-responders and responders before and after Abatacept treatment.

The revised figures have been updated accordingly in Fig 1I-K, and the figure legends.

[Comment 6]

The significance of using RRMS patients samples in Fig 5 is unclear, since no healthy control groups are included in these analyses.

[Response 6]

We appreciate the reviewer's important comment regarding the use of RRMS patient samples in Fig. 5. Although healthy donor samples were not included, we employed PBMCs from patients with non-inflammatory demyelinating diseases (non-IDD) as a disease control group. These individuals lack active immune-driven neuroinflammation, allowing comparison between inflammatory (RRMS) and non-inflammatory demyelinating conditions. Importantly, the non-IDD group provides a clinically relevant control by sharing demyelinating pathology without ongoing autoimmune inflammation, thereby enabling isolation of immune-specific alterations associated with RRMS (Nat Rev Neurol. 2012 Nov 5;8(11):647-56., Brain Commun. 2023 Jul 25;5(4):fcad206.).

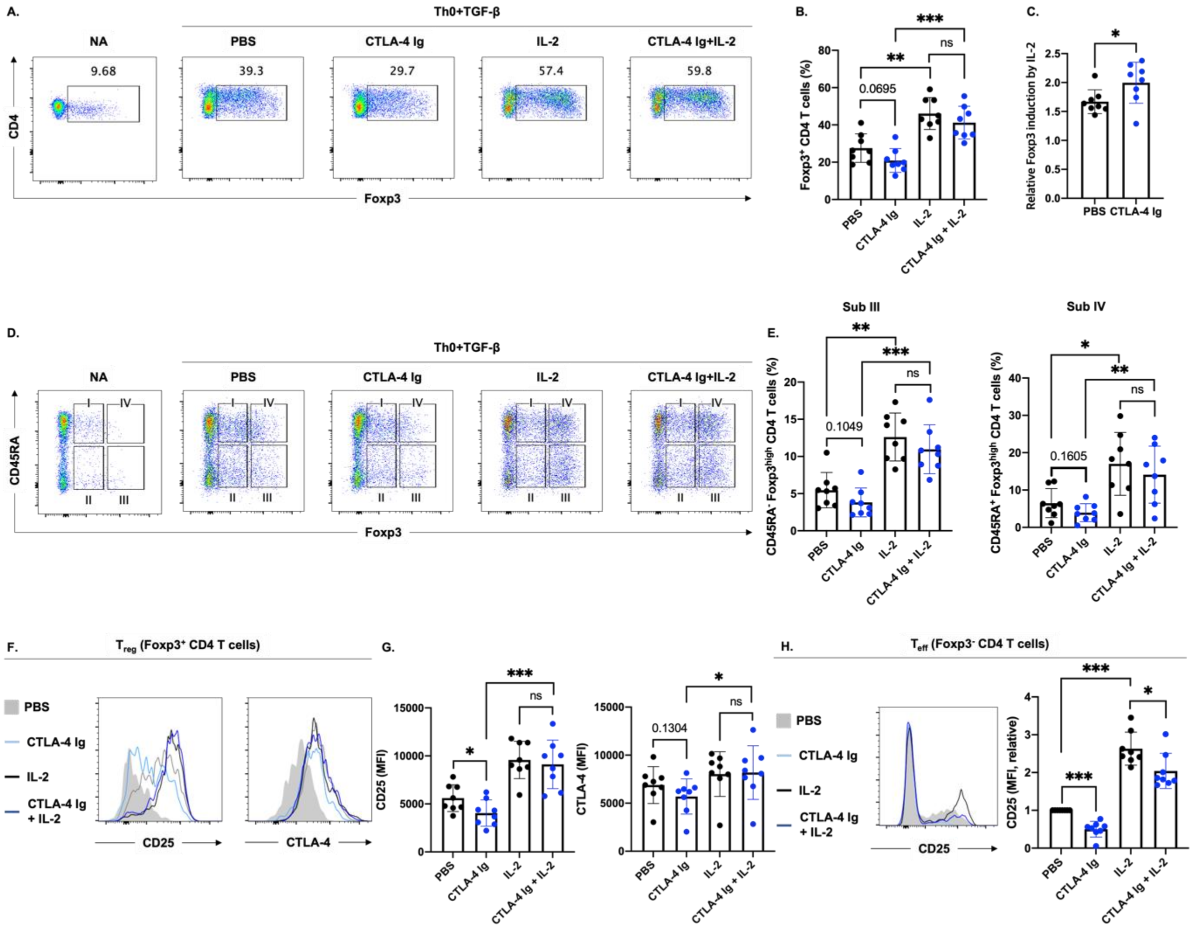

Fig. EV10. CTLA-4 Ig and IL-2 co-treatment increases Foxp3^{hi} Treg subpopulations and enhances suppressive molecule expression in human PBMCs from non-IDD patients.

(A, B) Representative FACS dot plot (A) and bar graph (B) exhibits Foxp3 induction. (C) IL-2 induced Foxp3 ratio with or without CTLA-4 Ig. (D, E) Representative FACS dot plot (D) and bar graph (E) of III (Effector/memory phenotype Treg cells) and IV (induced-naïve phenotype Treg cells) sub-population of Treg cells. (F) Representative histogram (G) and bar graph of CD25 and CTLA-4 expression in Foxp3^{+} CD4 T cells. (H) Representative histogram and bar graph of CD25 expression in Foxp3^{-} effector CD4 T cells.

Importantly, the combined effects of CTLA-4 Ig and IL-2 were consistently observed in both RRMS and non-IDD samples. This indicates that the proposed immunoregulatory mechanism is not restricted to MS-specific pathology but reflects a broader principle of T cell regulation under costimulation blockade.

Together, the human data supports the concept that IL-2 supplementation can functionally complement CTLA-4 Ig therapy by restoring Treg-mediated immune regulation, thereby providing a rationale for revisiting CTLA-4 Ig–based strategies in MS.

We have added in vitro human PBMC culture results using PBMCs from non-IDD patients in the revised Fig. EV10 with corresponding text in Results section (line 311-313), along with the list of patients in revised Appendix Table S1.

Referee #2 (Remarks for Author):

Summary: In this manuscript, Choi and colleagues explore the impact of CTLA-4 Ig and IL-2 treatment on Treg cells. To conduct their research, the authors utilize (i) in vivo mouse models, including the relapsing-remitting EAE model induced by proteolipid protein (PLP139-151), the MOG35-55-induced EAE model, and wild-type mouse models; (ii) human RNA-seq data from PBMCs of rheumatoid arthritis patients treated with CTLA-4 Ig (Abatacept); and (iii) in vitro T cell stimulation assays using cells isolated from mice and humans (MS patients). The authors report that while CTLA-4 Ig treatment reduces disease activity in the relapsing-remitting EAE model, discontinuation of treatment leads to a more severe relapse compared to untreated controls. This exacerbation may be linked to a reduction in $CD4^+$ $Foxp3^+$ Treg cells. To determine if a similar phenomenon occurs in humans, the authors analysed gene expression profiles of PBMCs from rheumatoid arthritis (RA) patients treated with Abatacept. Notably, non-responders exhibited decreased expression of Treg-associated genes. Further, in vitro experiments reported that antigen-specific stimulation of naïve 2D2 transgenic T cells in the presence of TGF-β, CTLA-4 Ig, and IL-2 led to more efficient differentiation into $CD25^+$ $Foxp3^+$ Treg cells, indicating a potential synergistic effect of CTLA-4 Ig and IL-2 in promoting Treg development. In the MOG35-55-induced EAE model, CTLA-4 Ig treatment significantly alleviated disease severity. Notably, co-administration of IL-2 further improved disease control, likely by mitigating the reduction in Treg cells associated with CTLA-4 Ig therapy. Finally, the study suggested a distinct mechanism by which CTLA-4 Ig modulated IL-2 signalling, by selectively dampening STAT3 activation without disrupting STAT5 pathways. When combined with IL-2, this selective signalling environment favoured the differentiation of effector Treg cells, driven by enhanced CD25/STAT5 and TGF-β/Smad3 activity. These insights suggest a novel therapeutic approach in which IL-2 and CTLA-4 Ig act synergistically to suppress pathogenic effector T cells while promoting regulatory T cell function, offering a refined strategy for treating autoimmune conditions.

[Answer]

We thank to the reviewer for the comprehensive and thoughtful summary of our study. We appreciate the reviewer’s careful evaluation of the in vivo mouse models, human transcriptomic analyses, and in vitro T cell differentiation assays, as

well as the clear articulation of the proposed synergistic effects of CTLA-4 Ig and IL-2 on Treg biology. We are grateful for the recognition of the mechanistic insights regarding selective modulation of IL-2–STAT signaling and their relevance to disease control in autoimmune models. We have carefully considered all comments raised by the reviewer and have revised the manuscript accordingly. Below, we address each point in detail.

[Comment 1]

Broader immune cell profiling

While the authors report a more severe disease relapse following cessation of CTLA-4 Ig therapy, it remains unclear why this occurs, particularly given the observed reduction in IL-17-producing CD4^{+} T cells (Fig. 1F) and the apparent maintenance of a favourable Treg-to-effector ratio (Fig. 1G). This raises questions about other immunological mechanisms that may contribute to disease exacerbation. The current analysis is limited to T cell subsets, and the study would benefit from a broader investigation of immune components involved in disease progression. In particular, assessing the activation status of antigen-presenting cells (APCs)-for instance, by evaluating CD80/CD86 expression-could provide valuable insights. Additionally, profiling other proinflammatory cytokines such as IFN-γ and IL-6 may help clarify the underlying mechanisms driving disease severity after treatment withdrawal.

These aspects should also be addressed in the RNA-seq data of the RA patient.

Moreover, the results in Figure 2 indicate that Treg cell differentiation is increasing while effector T cell differentiation is suppressed. Unlike the authors' statements, such an experimental setup in Figure 2 is not designed to measure effector T cell activation. Therefore, the authors need to revisit their conclusions and correct their statements on T cell activation.

[Response 1-1]

We would like to thank the reviewer for the helpful comments. We agree that disease relapse in SJL mice following cessation of CTLA-4 Ig treatment is unlikely to be solely attributable to reduced Treg numbers. To clarify whether reductions in Treg cells and IL-17–producing effector T cells provide meaningful insight during the relapse phase, we reintroduced the dNP2-ctCTLA-4 treatment group as a control in Fig. 1 using our pre-existing dataset.

dNP2-ctCTLA-4 is a cell-penetrating synthetic peptide derived from the cytoplasmic domain of CTLA-4, which our group has previously characterized (Adv Sci (Weinh). 2021 May 5;8(14):2004973.). Although this treatment group was included in the original experiments, it was omitted from the initially submitted version of Fig. 1 for simplicity. In response to the reviewer's suggestion, we have now restored the full dataset. This comparison revealed distinct outcomes between the two treatments: whereas CTLA-4 Ig reduced both IL-17A^{+} effector T cells and Foxp3^{+} Treg cells, dNP2-ctCTLA-4 preserved the Treg population while still reducing IL-17A^{+} cells. These findings indicate that the decrease in Treg frequency observed with CTLA-4 Ig is not merely a secondary consequence of reduced inflammation, but rather reflects a treatment-specific effect.

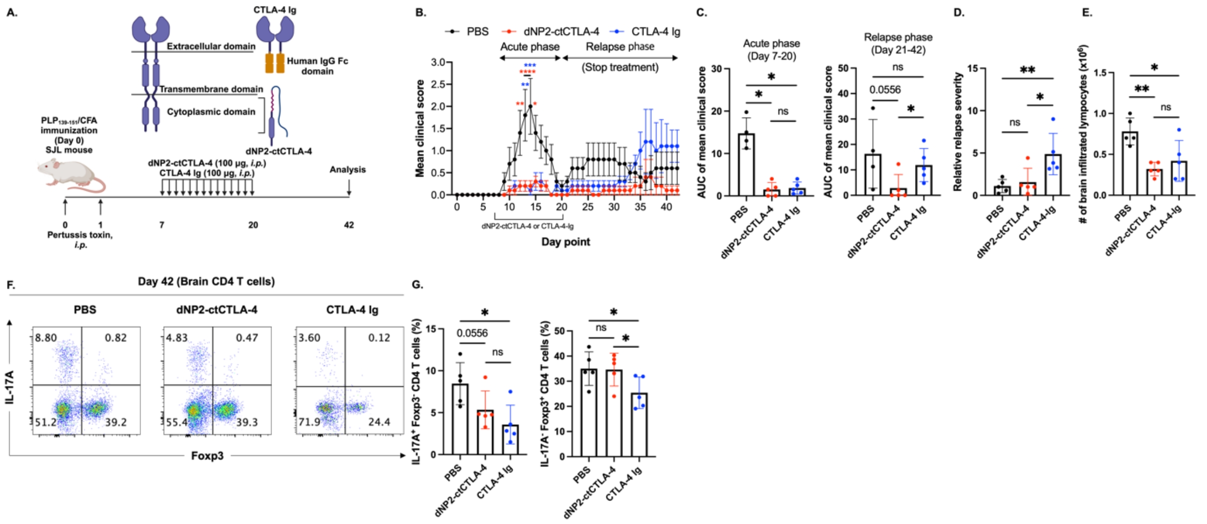

Revised Fig 1A-G. dNP2-ctCTLA-4 suppressed acute disease and prevented relapse in relapse-remitting EAE, unlike CTLA-4 Ig.

(A) Experimental scheme used to induce EAE. (B) Clinical score of EAE. (C) Comparison of disease score AUC between peak stage and relapse stage. (D) Relative relapse severity. (E) The number of brain-infiltrated lymphocytes. (F, G) Proportion of brain infiltrated IL-17A^{+} Foxp3^{-} and IL-17A^{-} Foxp3^{+} CD4 T cells. Representative FACS dot plot (F) and bar graph (G).

We fully agree with the reviewer that relapse in the SJL relapsing–remitting EAE model cannot be exclusively dependent on peripheral T cell frequencies. Previous studies have demonstrated that Treg depletion is sufficient to precipitate exacerbated relapse in SJL/PLP EAE (J Neuroimmunol. 2006 Mar;172(1-2):73-84.). In addition, enhanced APC activation, characterized by increased expression of CD80/CD86 and MHC-II, has been proposed as a contributing factor during the relapse phase in this model (Biochim Biophys Acta. 2011 Feb;1812(2):265-74.).

In our SJL EAE cohort, we did not observe significant differences in proinflammatory cytokine expression within the $CD4^-$ brain-infiltrated immune cell compartment between CTLA-4 Ig–treated and control mice, indicating that APC-derived cytokine output was not broadly enhanced at the time of relapse. In contrast, cytokine expression within brain CD4 T cells was reduced following CTLA-4 Ig treatment, consistent with attenuated T cell activation (Figure R2).

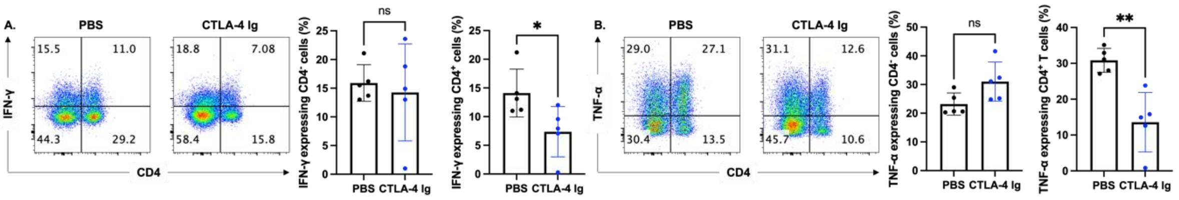

Figure R2. CTLA-4 Ig does not appear to suppress cytokine production of $CD4^-$ population in brain.
(A) Expression of IFN-γ in $CD4^-$ cells and $CD4^+$ T cells. (B) Expression of TNF-α in $CD4^-$ cells and $CD4^+$ T cells.

We note that reduced costimulatory capacity may contribute to this T cell–restricted effect. While CD86 expression was not directly assessed in the SJL model, we observed a reduction in CD86 expression within spinal cord–infiltrating $CD4^-$ $CD8^-$ immune cells in a conventional $MOG_{35\text{-}55}$ induced EAE model following CTLA-4 Ig treatment (Figure R3).

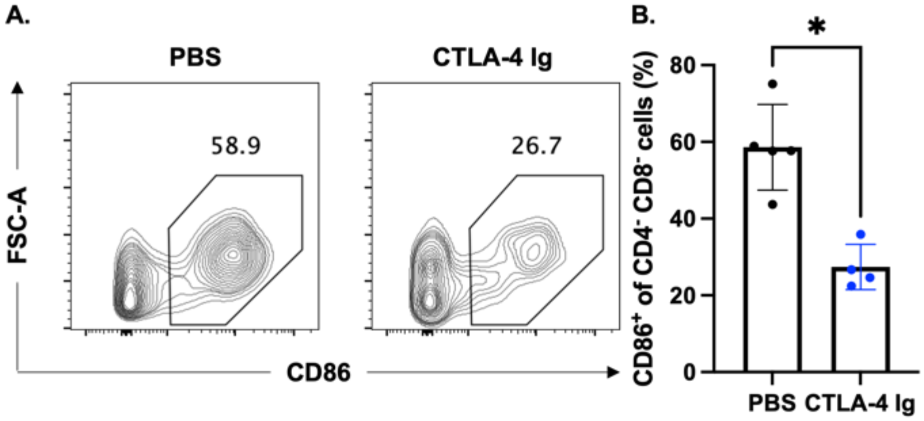

Figure R3. CTLA-4 Ig reduces CD86 expression of spinal cord-infiltrated $CD4^-$ $CD8^-$ population.
(A) Representative contour plot of CD86 expression in $CD4^-$ $CD8^-$ cells in spinal cord and (B) bar graph.

Consistent with these findings, transcriptomic analyses of RA patient samples revealed that expression of *CD80* and *CD86* were reduced after Abatacept treatment,

supporting the notion that CTLA-4 Ig treatment does not promote a generalized APC-driven inflammatory state (Figure R4).

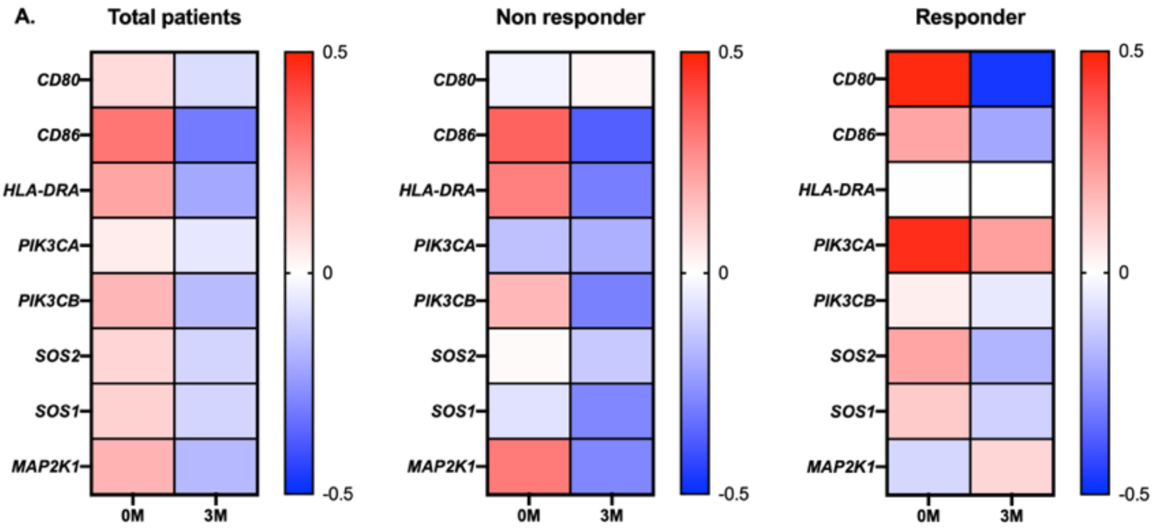

Figure R4. Reduced B7 molecules and CD28-related signaling molecules in RA patients after Abatacept treatment.

(A) Gene expression of B7 molecules and CD28 signaling-related molecules in RA patients before and after Abatacept treatment.

Taken together, although APC activation could be implicated as a potential contributor to relapse in SJL EAE, as pointed out by the reviewer, our data demonstrate that CTLA-4 Ig treatment does not affect cytokine production derived from non-T cell population, while it significantly suppresses APC activation.

In the revised manuscript, we have revised Fig. 1A to G including dNP2-ctCTLA-4 treatment group with corresponding sentences in Result section (line 102-120).

[Response 1-2]

We would like to thank the reviewer for the construct comments. To assess effector T cell differentiation more appropriately, we performed additional experiments under Th17-polarizing conditions (TGF-β + IL-6), which are better suited for evaluating inhibitory function of CTLA-4 Ig in effector lineage differentiation. Under these conditions, CTLA-4 Ig alone significantly inhibited Th17 differentiation, and the combination of CTLA-4 Ig and IL-2 further increased Foxp3 induction while reducing IL-17A expression compared with CTLA-4 Ig alone (Fig 2B, C). These data demonstrate suppression of effector T cell differentiation concomitant with enhanced Treg generation under conditions permissive for effector lineage differentiation.

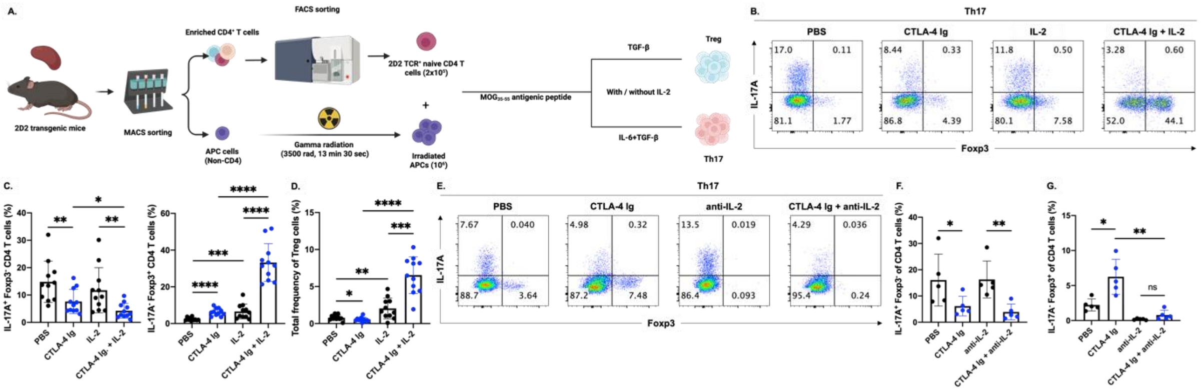

Revised Fig 2A-G. CTLA-4 Ig and IL-2 synergistically increase Treg cell differentiation while suppress effector T cell activation.

(A) Experimental flow of co-culture using FACS sorted naïve 2D2 TCR$^+$ CD4 T cells with APCs. (B) Representative FACS dot plot showing IL-17A and Foxp3 expression in CD4 T cells cultured with IL-2, with or without CTLA-4 Ig in Th17 polarized condition. (C) Bar graph of IL-17A$^+$ Foxp3$^-$ effector CD4 T cells and IL-17A$^-$ Foxp3$^+$ iTreg cells. (D) Total frequency of IL-17A$^-$ Foxp3$^+$ iTreg cells. (E-G) Expression of IL-17A and Foxp3 in presence of CTLA-4 Ig with 5 μg/ml of anti-IL-2 neutralization antibody in Th17 polarized condition. (E) Representative dot plot of IL-17A and Foxp3 expression in CD4 T cells. (F) Bar graph of IL-17A$^+$ Foxp3$^-$ CD4 T cells. (G) Bar graph of IL-17A$^-$ Foxp3$^+$ CD4 T cells.

In the revised manuscript, we updated new Fig 2B-G to include the Th17 experiments and reorganized original Fig 2B-E to revised Fig 2H-K. We also updated and revised the text to clarify the description of the Results section (line 174-188).

[Comment 2]

"Synergistic effect" needs further investigation

In order to conclude a synergistic effect of two compounds, in this case CTLA-4 Ig and IL-2, the effects of either treatment as a monotherapy should be tested as well. The treatment efficacy of IL-2 alone is for example missing in Figure 3 and therefore a claim on synergism is not possible. Particularly, the presented results in Figure 3H indicates that IL-2 does not significantly restore eTreg diminished by CTLA-4 as claimed in the figure title. On a side note, the injected amount of IL-2 (20 ng per injection) seems far too low, even for "low-dose IL-2" in mouse experiments (expected "low-dose IL-2" treatments would usually contain 1-5 microgram of IL-2 per injection)

[Response 2]

We thank the reviewer for the comments regarding the missing experimental group and the assessment of statistical significance. The IL-2–alone group was originally included in the EAE experiments, but it was omitted from the initial presentation to reduce graphical complexity. In addition, to rigorously assess the statistical significance of the CTLA-4 Ig + IL-2 group compared with CTLA-4 Ig alone, we performed additional independent EAE experiments. In total, 4 independent EAE datasets (n=11-19 per group) were analyzed and are now presented in the revised Fig. 3. Therefore, IL-2 alone, as used in this study, did not ameliorate EAE or induce Treg expansion. However, when combined with CTLA-4 Ig, IL-2 led to a greater reduction in EAE clinical scores and restored the effector Treg (eTreg) population that was reduced by CTLA-4 Ig treatment.

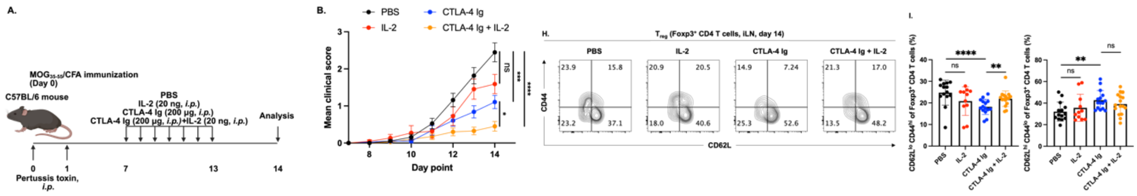

Revised Fig 3A-B and H-I. IL-2 synergistically ameliorate EAE disease with restoration of eTreg cells diminished by CTLA-4 Ig.

(A) Experimental scheme for inducing EAE. (B) Clinical disease score after EAE induction. (H) Representative dot plot of naïve ($CD62L^{hi}$ $CD44^{lo}$) and effector/memory ($CD62L^{lo}$ $CD44^{hi}$) T cells in $Foxp3^{+}$ CD4 Treg cells of iLN analyzed by flow cytometry. (I) Bar graph indicating proportion of eTreg cells and cTreg cells.

In the revised manuscript, we have updated Fig 3 to include IL-2 alone group to conclude synergistic effects of CTLA-4 Ig and IL-2 in EAE treatment. In addition, we updated new Fig 3I which is original Fig. 3H with additional sets of EAE and statistical significances.

Regarding IL-2 dosing, we agree that the term “low-dose IL-2” is commonly used to refer to doses of approximately 1–5 μg per injection, particularly when administered in complexed formulations (e.g., IL-2/JES6-1) to achieve Treg-selective expansion (Immunity. 2015 May 19;42(5):815-25., Science. 2006 Mar 31;311(5769):1924-7.). However, in this study we used uncomplexed (“naked”) IL-2 which in principle could promote both Treg cell expansion and differentiation, while recognizing that uncomplexed IL-2 can also act on CD25-expressing activated effector T cells. Therefore, we carefully titrated the dose of uncomplexed (“naked”) IL-2 to limit excessive effector T cell expansion and Treg expansion based on previous studies (Elife. 2021 Feb 22:10:e57417., Int Immunopharmacol. 2021 Aug:97:107663.). When we administered different dose of IL-2 (20, 500 ng per mouse) intraperitoneally every day for 7 days in EAE mice, 500 ng IL-2 showed significant increase of $CD25^+$ $Foxp3^+$ Treg cells while 20 ng IL-2 treatment did not induce Treg cells (Figure R5 below).

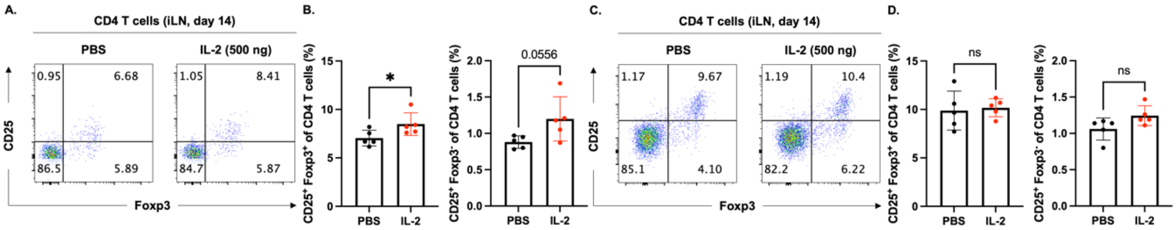

Figure R5. Effects of different IL-2 doses on Treg cells in the EAE model.
(A, B) Proportions of $CD25^+$ $Foxp3^+$ and $CD25^+$ $Foxp3^-$ CD4 T cells in the iLN after intraperitoneal administration of 500 ng IL-2 daily for 7 days. (C, D) Proportions of $CD25^+$ $Foxp3^+$ and $CD25^+$ $Foxp3^-$ CD4 T cells in the iLN after intraperitoneal administration of 20 ng IL-2 daily for 7 days.

In addition, the combination of CTLA-4 Ig with 500 ng of IL-2 failed to produce synergistic effects with CTLA-4 Ig in reducing EAE clinical symptoms (Figure R6 below).

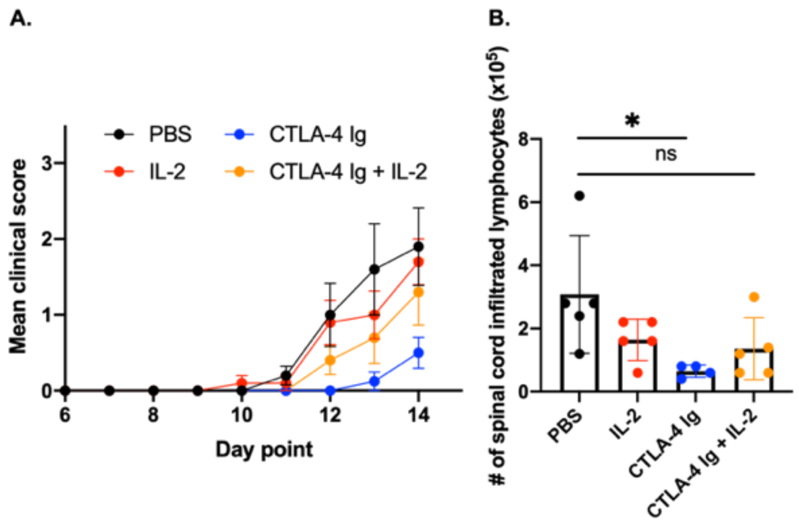

Figure R6. High-dose uncomplexed IL-2 attenuates CTLA-4 Ig–mediated disease control in EAE. (A) Mean clinical score after EAE induction. (B) Cell number of spinal cord infiltrated lymphocytes.

Therefore, we chose 20 ng as a suboptimal dose for this study.

In the revised manuscript, we have also added an explanation in the Discussion section for using suboptimal dose (20 ng) of IL-2 and for selecting the titrated optimal dose (line 437-446).

[Comment 3]

Need for in vivo validation of CTLA-4 Ig-mediated modulation of IL-2 signaling in EAE

The manuscript would benefit from a more thorough investigation of the IL-2-driven signalling pathway in the in vivo setting. It remains unclear whether CTLA-4 Ig can inhibit IL-2-induced STAT3 phosphorylation, which could potentially shift the balance toward enhanced TGF-β/Smad2/3 signalling and increased Foxp3 expression, particularly in the EAE model. To support the proposed mechanism involving modulation of PI3K/STAT3 signalling by CTLA-4 Ig, the authors should extend their analysis beyond the in vitro findings presented in Figure 4. Specifically, ex vivo analysis of pSTAT3 and pSmad2/3 levels in $CD4^+$ T cells isolated from EAE mice would be critical to validate the proposed pathway and strengthen the translational relevance of combining CTLA-4 Ig with IL-2 as a therapeutic strategy.

[Response 3]

We agree on the importance of the in vivo relevance of the signaling pathway we propose, in which IL-2–induced STAT3 activation under antigen stimulation is regulated by costimulatory blockade mediated by CTLA-4 Ig, thus increasing pSmad2/3 and Foxp3 expression. This pathway represents an important mechanism underlying our findings.

In the EAE disease context, we performed additional EAE experiments for ex vivo phospho-flow analyses of $CD4^+$ T cells isolated from the draining inguinal lymph nodes of $MOG_{35–55}$–immunized C57BL/6 mice on day 7. The mice treated with IL-2 (20 ng per mouse, day 0-7) showed increased pattern of STAT5 phosphorylation ($p=0.0556$) which was synergistically increased by CTLA-4 Ig combination (Figure R7A, B). Importantly, we observed a reproducible increase in C-terminal Smad2/3 phosphorylation in the CTLA-4 Ig and IL-2 combination group, accompanied by restoration of Foxp3 expression in $Foxp3^+$ CD4 T cells (Figure R7C, D). These data suggest that in vivo restoration of Treg cells during EAE by CTLA-4 Ig and IL-2 would mediate enhanced TGF-β/Smad and STAT5 signaling.

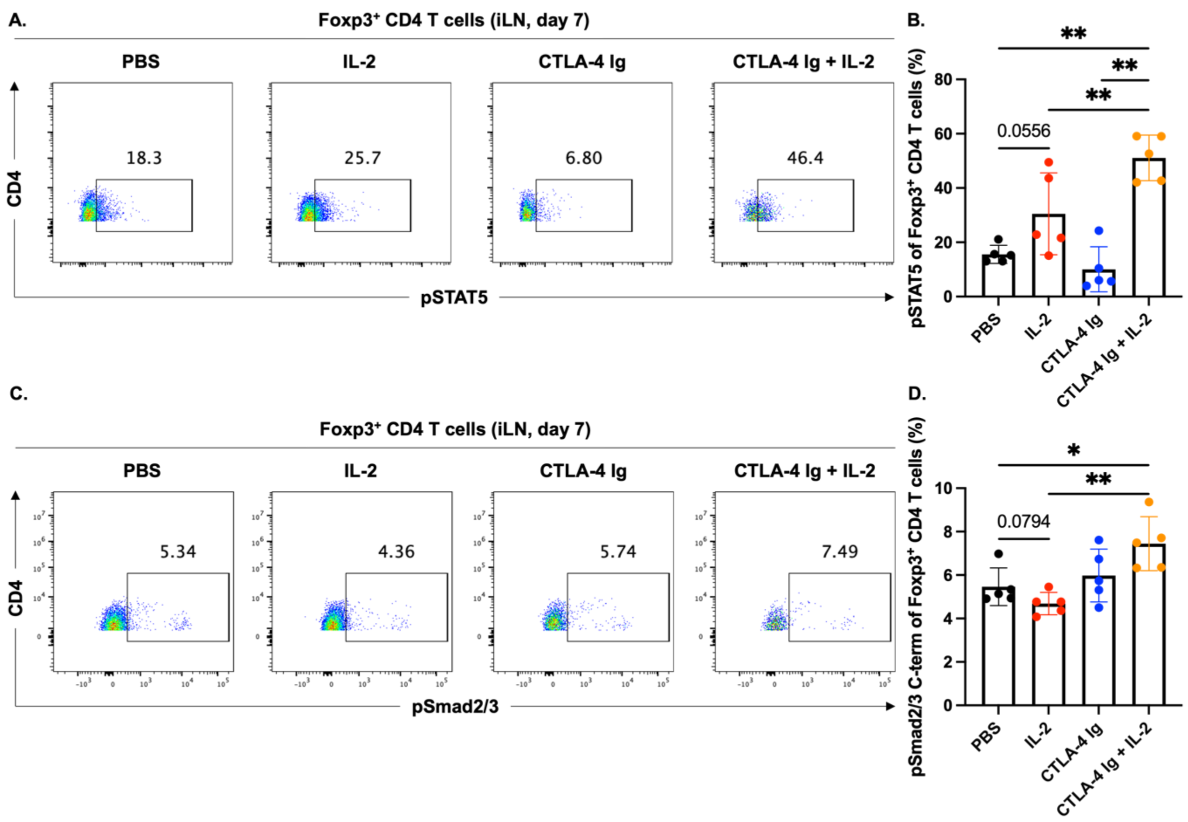

Figure R7. Expression of pSTAT5 (A, B), pSmad2/3 C-term (C, D) and pSTAT3 (E, F) in Foxp3^{+} CD4 T cells isolated from iLN of EAE-induced mice in day 7.

However, unfortunately, we could not detect pSTAT3 in isolated T cells from EAE mice.

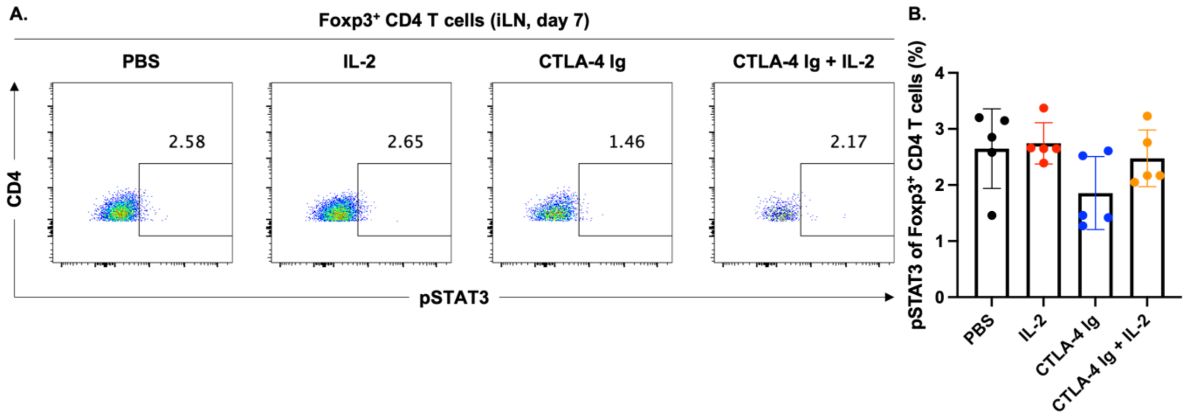

Figure R8. Expression of pSTAT3 could not detected in Foxp3^{+} CD4 T cells isolated from iLN of EAE-induced mice in day 7.

(A) Representative FACS dot plots of pSTAT3 expression in Foxp3^{+} CD4 T cells. (B) Bar graph indicating expression of pSTAT3.

This discrepancy may be explained by the polyclonal nature of CD4 T cells in the draining lymph nodes of C57BL/6 mice, in which MOG-specific T cells constitute only a small fraction of the total CD4 T-cell population (J Neuroinflammation. 2024 Oct 8;21(1):253). Alternatively, STAT3 phosphorylation during EAE may be transient, and antigen-specific signals could be diluted in bulk ex vivo flow cytometric analyses.

Alternatively, we designed a separate in vivo stimulation experiment in which anti-CD3 antibody was administered subcutaneously for 2 hours in mice together with 20 ng of IL-2. In this experiment, we observed a robust increase in pSTAT3 in draining lymph node Foxp3$^+$ CD4 T cells following combined anti-CD3 and IL-2 administration, compared with either anti-CD3 alone or IL-2 alone (Figure R9). These results suggest that IL-2–mediated STAT3 activation requires concomitant TCR stimulation in vivo, which may explain the technical limitation in detecting pSTAT3 signals in T cells from the MOG-specific activation model in EAE mice by ex vivo flow cytometry.

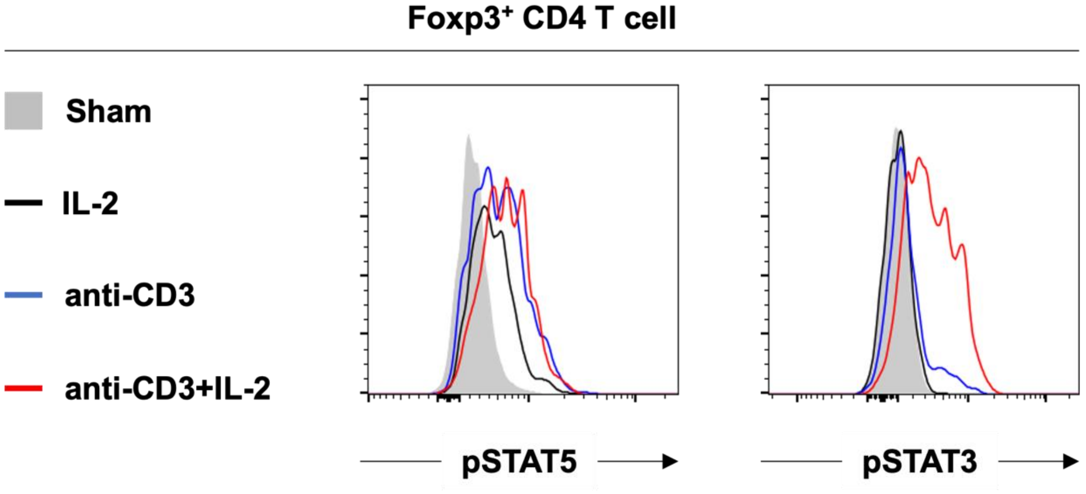

Figure R9. Intact mice were stimulated by subcutaneous injection into the iLN with anti-CD3 (20 μg), IL-2 (20 ng), or their combination for 2 hours. The expression of pSTAT5 and pSTAT3 was analyzed in Foxp3$^+$ CD4 T cells.

In the revised manuscript, we have added bar graph indicating expression of pSTAT5 and pSmad2/3 C-term of Figure R7 in revised Figure 3L, along with the corresponding FACS dot plot data in Appendix Figure S3 and the relevant text in the Results section (line 225-232).

[Minor Comments]

1. P.4 Please add a reference for the following sentence: "However, its expansion to other autoimmune diseases has still been largely limited in clinical trials, primarily due to its unintended effects on Treg cell depletion."

[Response]

We thank the reviewer for pointing this out. We have now added an appropriate reference supporting this statement in the revised manuscript. These studies indicate that clinical expansion of Abatacept into other autoimmune diseases has been limited, often accompanied by alterations in immune regulatory pathways, including those associated with regulatory T cells, highlighting disease-specific constraints of costimulation blockade. (Mult Scler. 2017 Apr;23(5):686-695., Gastroenterology. 2012 Jul;143(1):62-69.e4., Am J Respir Crit Care Med. 2013 Mar 1;187(5):494-501.) (line 72-74).

2. P.16: “In this study, PBBM’s from RRMS”–" - Please corre“t "P’B”'s" “o "P’M”'s"

[Response]

We appreciate the reviewer for catching this error. The typographical mistake has been corrected to “PBMCs” in the revised manuscript.

3. P.22: According to the Materials section on page 22, the study used the following setup "Cells were cotreated with 5 μM of dNP2-ctCTLA-4 or 0.5 μM of CTLA-4 Ig." Is this correct? If so, please indicate this tratment in the Figures.

[Response]

We thank the reviewer for this comment. The description in the Materials section referring to cotreatment with dNP2-ctCTLA-4 and CTLA-4 Ig reflected an earlier experimental design that was later revised. To avoid confusion, we have now clarified and updated the manuscript accordingly.

dNP2-ctCTLA-4 is a cell-penetrating peptide developed in our group, consisting of the cytoplasmic signaling domain of CTLA-4 conjugated to the dNP2 peptide. This reagent was used in a limited set of in vitro experiments to assess whether

intracellular CTLA-4 signaling modulates Treg induction under TGF-β–containing conditions. Consistent with our previous work, dNP2-ctCTLA-4 promoted Treg induction in a TGF-β–dependent manner (Fig. EV4A-C). However, unlike CTLA-4 Ig, it did not further enhance Treg differentiation in the presence of IL-2 (Fig. EV4D).

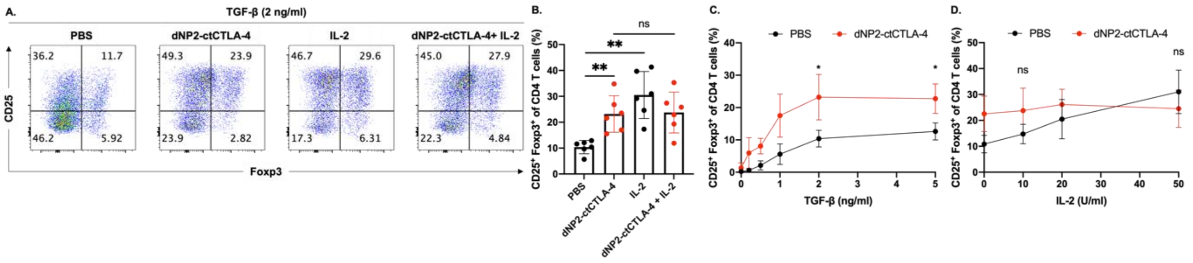

Fig. EV4. dNP2-ctCTLA-4 enhances TGF-β-dependent, but not IL-2-driven, Foxp3 induction.

(A-D) Naïve CD4 T cells sorted from 2D2 transgenic mice were cultured with irradiated APCs and stimulated by MOG_{35-55} antigenic peptide in presence of (A-D) TGF-β (Th0+TGF-β) or (E, F) IL-6+TGF-β (Th17) with or without 50 U/ml of IL-2, treatment of dNP2-ctCTLA-4 (5 μM) for 3 days. (A) Representative dot plot of $CD25^+$ $Foxp3^+$ CD4 T cells and (B) bar graph. (C) Dose dependency of TGF-β of $CD25^+$ $Foxp3^+$ Treg induction by dNP2-ctCTLA-4 in absence of IL-2. (D) Dose dependency of IL-2 of $CD25^+$ $Foxp3^+$ Treg induction by dNP2-ctCTLA-4 in fixed concentration of TGF-β.

These data are now explicitly presented in Fig. EV4, and all corresponding treatments are clearly indicated in the figure and legend (line 197-199). We have also revised the Materials and Methods section to ensure consistency between the experimental description and the data shown in the figures (line 547-548).

4. P.25: According to the Materials section on page 25, the study used the following setup for the relapsing-remitting EAE mouse model: "100 μg of dNP2-ctCTLA-4 and 100 μg of CTLA-4 Ig (Orencia, BMS) was injected intraperitoneally daily from day 7 to day 20 during early phase."

Is this correct? If so, please indicate this tratment in the Figures.

[Response]

We thank the reviewer for pointing out this issue. While the description in the Materials section was accurate, the original manuscript did not fully reflect this treatment scheme in the corresponding figures. In the revised manuscript, we have now included the dNP2-ctCTLA-4 treatment group in Figure 1, thereby ensuring that

the data presentation is fully consistent with the experimental setup described in the Materials section.

In the relapsing–remitting EAE model, mice were treated intraperitoneally with 100 μg of dNP2-ctCTLA-4 or 100 μg of CTLA-4 Ig daily from day 7 to day 20, corresponding to the early phase of disease. As now shown in Fig. 1, dNP2-ctCTLA-4 markedly suppressed disease severity during the acute phase and, in contrast to CTLA-4 Ig, effectively prevented disease relapse following treatment cessation. Notably, while CTLA-4 Ig treatment was associated with disease exacerbation after withdrawal, dNP2-ctCTLA-4–treated mice exhibited sustained disease control with minimal relapse.

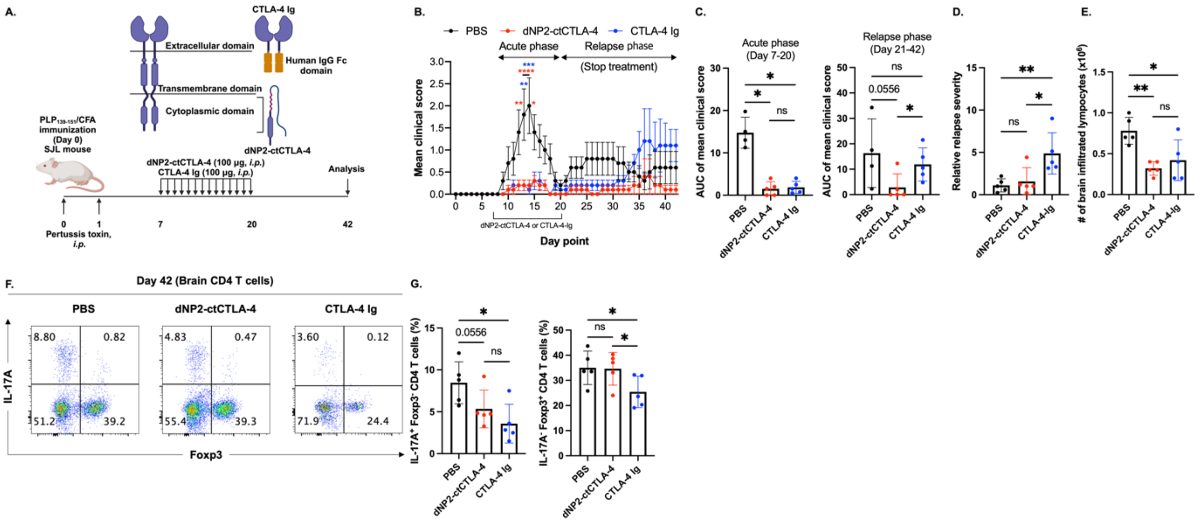

Fig 1A-G. dNP2-ctCTLA-4 suppressed acute disease and prevented relapse in relapse-remitting EAE, unlike CTLA-4 Ig.

(A) Experimental scheme used to induce EAE. (B) Clinical score of EAE. (C) Comparison of disease score AUC between peak stage and relapse stage. (D) Relative relapse severity. (E) The number of brain-infiltrated lymphocytes. (F, G) Proportion of brain infiltrated IL-17A^{+} Foxp3^{-} and IL-17A^{-} Foxp3^{+} CD4 T cells. Representative FACS dot plot (F) and bar graph (G).

In the revised manuscript, we include dNP2-ctCTLA-4 group in revised Fig 1A-G in Results section (line 102-120).

Referee #3 (Remarks for Author):

In this work, Nam and colleagues present a series of experiments that explore the mechanism through which treatment with CTLA4.Ig might decrease the abundance of regulatory T cells. In general, I found the manuscript difficult to read and the flow of experiments difficult to follow. They interchangeably use different mouse models, observations from patients, and in vivo activation of murine and human cells and draw parallels that are not obvious to prove their points. The experiments lack an adequate sample size and, in some cases, important experimental controls. I do not feel the authors are able to adequately prove any of their claims.

[Answer]

We appreciate the reviewers' critique to be better clarification on our observation and hypothesis with logical flow for both experimental design and text writing. For the last 7 months, we performed 4 independent sets of EAE experiments to increase sample size and obtain statistical significance between groups. We also performed intensive in vitro experiments to satisfy our hypothesis with statistical significance. Finally, we re-evaluated SJL mouse EAE and relevant in vitro T cell experiments with dNP2-ctCTLA-4 group. Although $PLP_{139-151}$ EAE and MOG_{35-55} EAE is not an equivalent model, regular EAE is enough and efficient model to address our hypothesis that CTLA-4 Ig reduces Treg during the treatment while IL-2 combination restores them with synergistic effects. Finally, we carefully revised the text of the manuscript thoroughly to clarify the research flow and clarify what we would like to claim. We believe this revised version of manuscript would satisfy the reviewer's concerns to prove our hypothesis.

[Comment 1]

The manuscript is written in a very confusing manner. Professional English editing may be desirable.

[Response 1]

We have carefully revised the entire manuscript to improve clarity, coherence, and logical flow. The order of experimental sections has been reorganized to better reflect the conceptual progression of the study, and all text has been professionally

proofread to ensure clarity and readability. We believe these revisions substantially improve the readability of the manuscript.

[Comment 2]

Figure 1. CTLA4.Ig reduces Treg numbers by reducing CD28 signaling. The main point of the figure is that though CTLA4.Ig may decrease effector T cell activation, through the same mechanism it causes a drop in Treg abundance that may be counterproductive in the setting of autoimmune diseases. The authors provide two sets of data to prove their point: (1) EAE in mice treated with CTLA4.Ig or control, and (2) an analysis of transcriptomic data from patients with rheumatoid arthritis treated with abatacept. However, the data in mice is underpowered and not robust and the transcriptomic analysis fails to demonstrate their claim.

Specifically:

a. How many times was the experiment depicted in Fig. 1B to D repeated? The figure legend says 5 animals per group and only 5 dots are depicted. In my opinion, the experiment should be repeated with more mice, to determine whether there are differences between the groups.

b. Fig 1B has very large error bars and I doubt there are any statistical differences in Fig 1B after day 20. The same applies to Fig1C, where the differences do not seem statistically significant.

[Response a and b]

We acknowledge the potential limitation associated with the relatively small group size in the SJL EAE model (Fig. 1B-D). To address this concern and strengthen the statistical and biological robustness of the dataset, we implemented several analytical and experimental revisions in the revised manuscript.

To further strengthen the biological interpretation and provide an internal comparator, we included an additional treatment group using the cytoplasmic domain–derived CTLA-4 peptide, dNP2-ctCTLA-4, which has been reported to promote Treg induction (Adv Sci. 2021;8:2004973). As shown in the referenced study, continuous treatment with both dNP2-ctCTLA-4 and CTLA-4 Ig significantly inhibited disease relapse; however, the CTLA-4 Ig–treated group showed reduced Foxp3^{+} CD4^{+} T cells in spinal cord tissue (n = 4 per group). This previous study confirms CTLA-4 Ig effects on Treg reduction in EAE of SJL mice as a separate independent experiment.

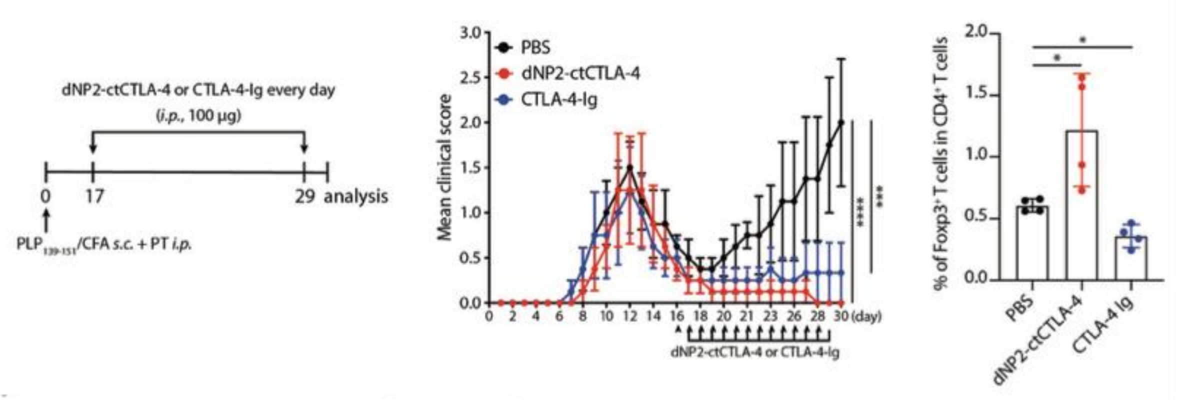

Adv Sci (Weinh). 2021 May 5;8(14):2004973.

In the present study, we confirmed that both CTLA-4 Ig and dNP2-ctCTLA-4 significantly suppressed disease activity during the acute phase (day 7–20). However, during the relapse phase, dNP2-ctCTLA-4 maintained long-term disease control consistent with previous reports, whereas the CTLA-4 Ig–treated group exhibited relapse comparable to the PBS control group, as reflected by both clinical scores and area-under-the-curve (AUC) analysis. This distinction was further supported by flow cytometric analyses showing that CTLA-4 Ig reduced not only IL-17A–producing CD4^{+} T cells but also Foxp3^{+} Treg frequencies, whereas dNP2-ctCTLA-4 preferentially preserved Treg populations within brain-infiltrating CD4^{+} T cells. These findings provide a mechanistic explanation for the relapse observed following CTLA-4 Ig withdrawal despite initial suppression of effector responses.

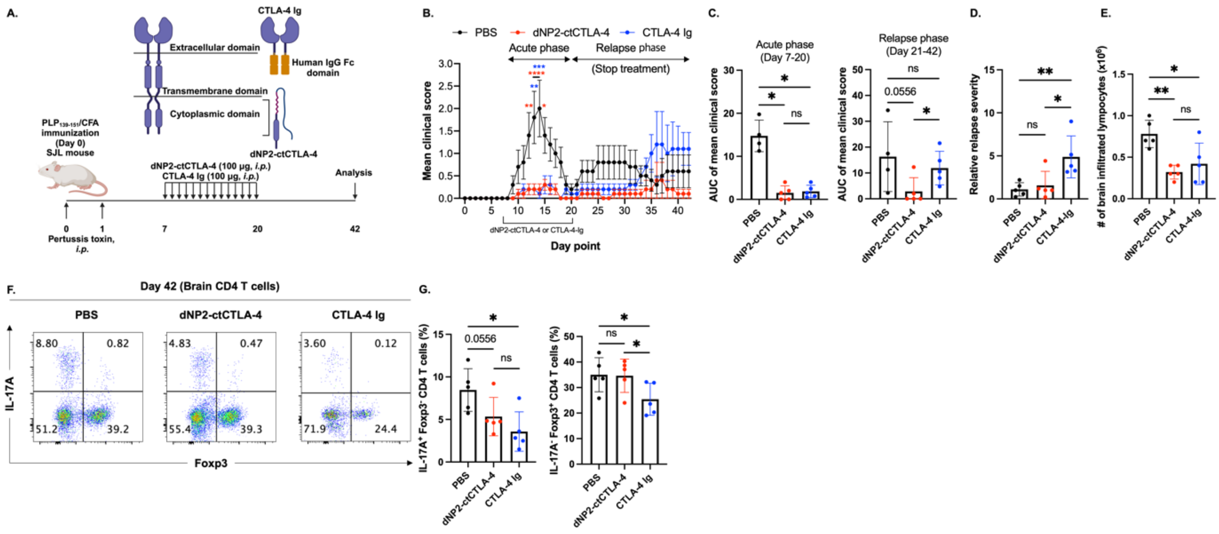

Revised Fig 1A-G. dNP2-ctCTLA-4 suppressed acute disease and prevented relapse in relapse-remitting EAE, unlike CTLA-4 Ig. (A) Experimental scheme used to induce EAE.

(B) Clinical score of EAE. (C) Comparison of disease score AUC between peak stage and relapse stage. (D) Relative relapse severity. (E) The number of brain-infiltrated lymphocytes. (F, G) Proportion of brain infiltrated IL-17A^{+} Foxp3^{-} and IL-17A^{-} Foxp3^{+} CD4 T cells. Representative FACS dot plot (F) and bar graph (G).

We further calculated the AUC for each phase (acute phase, day 7–20; relapse phase, day 21–42) and introduced a relative relapse severity index, defined as the ratio of relapse-phase AUC to acute-phase AUC. This index was significantly increased only in the CTLA-4 Ig–treated group, but not in the dNP2-ctCTLA-4 group. For the phase-specific AUC analysis, one PBS mouse that did not develop clinical disease was excluded from this particular calculation due to the absence of measurable disease activity, which may reflect technical variability in disease induction. Importantly, this sample was included in all other analyses, including clinical score curves and flow cytometric data, and its inclusion did not alter the overall interpretation.

Lastly, to improve the visualization and statistical interpretation of disease severity, we replotted the clinical score curves using the standard error of the mean (S.E.M.) instead of standard deviation, which previously resulted in large error bars that obscured intergroup trends. Importantly, the primary purpose of Fig. 1B was not to directly compare absolute disease severity between groups, but rather to assess the impact of CTLA-4 Ig withdrawal on disease relapse.

We revised Fig. 1A-G in the revised manuscript and the corresponding Results section (line 102-120).

c. Fig. 1E to G. When where those analyses performed? I assume they represent the same mice from Fig. 1B. It would be important to analyze CNS infiltration at earlier time points, after CTLA4.Ig treatment and before/during relapse.

[Response c]

The analysis time point was day 42, as indicated in the original manuscript (Fig. 1A). Figures 1E–G were derived from brain tissue. Although the y-axis label in the original Figure 1E specified “# of brain-infiltrated lymphocytes,” this information

was not explicitly indicated in revised Fig 1F and 1G, although it was described originally in the figure legend. However, as the reviewer pointed out, to improve clarity and provide clearer information regarding the experimental scheme, analysis time point, and tissue source, we have added the time point and tissue information at the top of each graph in the revised manuscript.

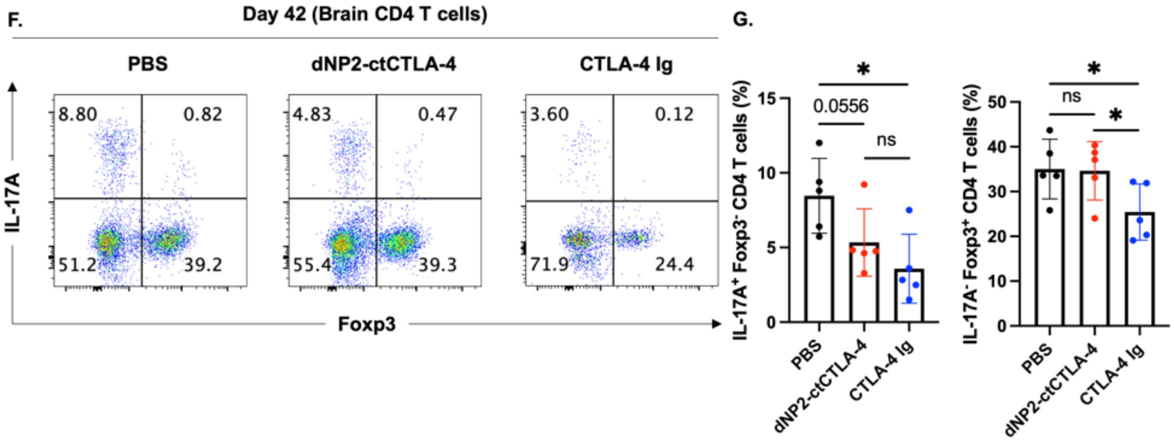

Revised Fig 1F, G. Proportion of brain infiltrated IL-17A$^+$ Foxp3$^-$ and IL-17A$^-$ Foxp3$^+$ CD4 T cells. Representative FACS dot plot (F) and bar graph. (F, G) Proportion of brain infiltrated IL-17A$^+$ Foxp3$^-$ and IL-17A$^-$ Foxp3$^+$ CD4 T cells. Representative FACS dot plot (F) and bar graph (G).

We agree with the reviewer's concern regarding the comparison between early and late time points during relapse. In our previous study, as mentioned above (**Response a and b**), analysis during the suppression phase of CTLA-4 Ig treatment demonstrated a reduction in Foxp3$^+$ Treg cells, indicating that CTLA-4 Ig rapidly impairs Treg stability, and that this reduction is not autonomously restored after treatment cessation. A similar acute effect of CTLA-4 Ig–mediated Treg reduction was also confirmed in wild-type mice, as shown in Fig. EV5D–F.

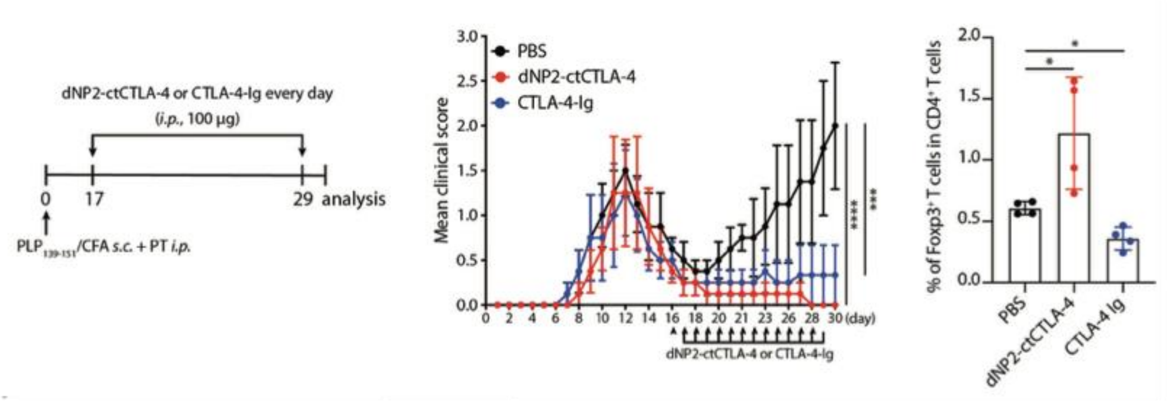

Adv Sci (Weinh). 2021 May 5;8(14):2004973.

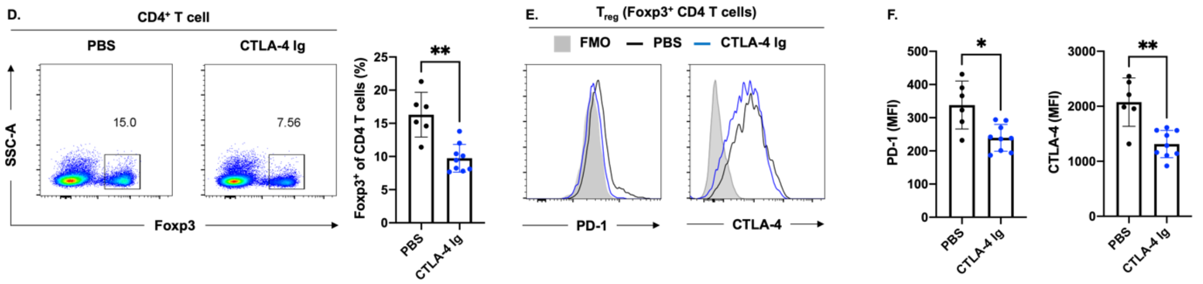

Fig. EV1D-F. Costimulation blockade by CTLA-4 Ig reduces proportion of Treg cells in steady-state mice.

(D) Expression of Foxp3 in CD4 T cells was analyzed by flow cytometry. (E, F) PD-1 and CTLA-4 expression in Foxp3$^+$ CD4 T cells.

We have stated in the Results section (line 121–132) that CTLA-4 Ig administration reduces Treg cells at early time points in intact mice. In addition, we have added corresponding sentences and references indicating costimulatory signaling and IL-2 signal are required for Treg maintenance and differentiation in Discussion section (line 391-412).

e. The transcriptomic analysis performed with published data from patients with rheumatoid arthritis fails to yield any significant data. If the authors wish to evaluate the effects of abatacept in Treg numbers and/or function, it would have been more appropriate to evaluate Tregs using flow cytometry. For example, based on lower CD28 transcription, the authors claim that CD28 expression may be a factor involved in response to abatacept. However, they ignore if CD28 is differentially expressed and ignore the pattern of expression of this receptor in Tregs and other T cells. Then, they identify genes that encode signaling molecules as important and they propose this is an effect of “attenuation of costimulation”. In my opinion, these claims should be supported by data in cells at the protein level.

[Response e]

We appreciate the reviewer’s concern and fully agree that bulk PBMC transcriptomic data alone do not provide cell type–specific resolution (e.g., Tregs versus other T-cell subsets) nor direct information on protein expression or functional activity. In human peripheral blood, Treg cells typically constitute only a small fraction of total CD4$^+$ T cells (approximately 3–7%), and therefore CD28 transcript

levels in bulk RNA-seq analyses are expected to be predominantly influenced by non-Treg CD4+ and CD8+ T-cell populations.

Accordingly, bulk RNA-seq data cannot definitively determine whether the reduced CD28 expression observed in non-responders reflects changes within a specific T-cell subset. Nevertheless, we consistently observed lower overall CD28 transcript levels in PBMCs from non-responding patients, which may be associated with diminished responsiveness to CTLA-4 Ig. In the present study, we did not intend to over-interpret this observation as a cell-intrinsic mechanism. Rather, we considered it descriptive, hypothesis-generating information that informed subsequent experimental design.

Furthermore, previous studies have reported that the proportion of CD4+ CD28− T cells is increased in the peripheral blood of patients with rheumatoid arthritis (Mol Med. 1996 Sep;2(5):608-18., Arthritis Res Ther. 2007;9(5):R87.). This phenomenon may be related to altered responsiveness to CTLA-4 Ig, as clinical studies have shown that patients with a higher frequency of CD28^low or CD28− T cells exhibit reduced therapeutic responses to abatacept (J Rheumatol. 2010;37:911–916, J Rheumatol. 2011;38:2105–2111).

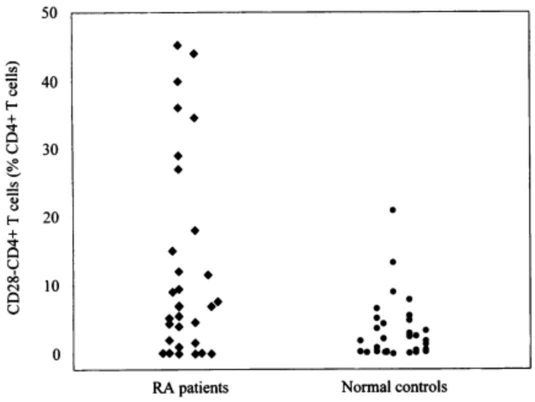

FIG. 1. Expansion of the CD4+ CD28− compartment in RA patients

PBMC from 30 healthy individuals and 30 RA patients were analyzed by two-color FACS for the presence of CD4+ CD28− T cells.

Mol Med. 1996 Sep;2(5):608-18.

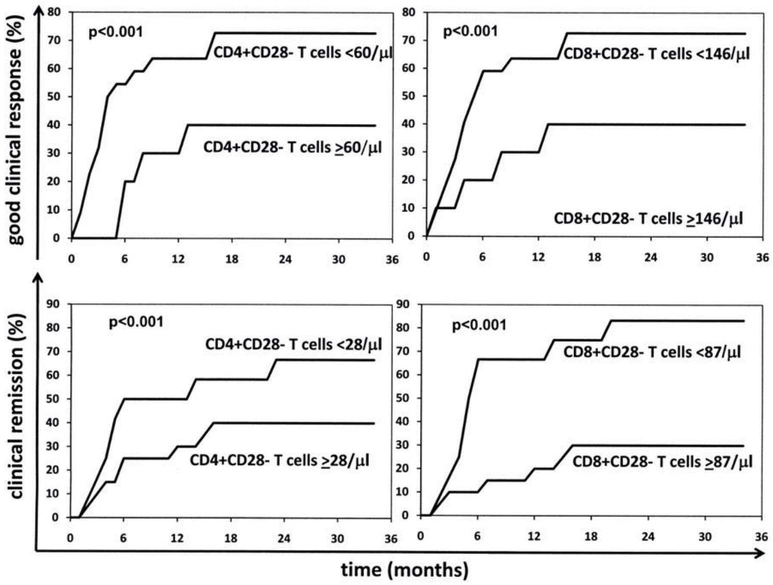

J Rheumatol. 2011 Oct;38(10):2105-11.

Accordingly, we did not intend our transcriptomic analysis to serve as definitive evidence for changes in Treg number or function, but rather exploratory, hypothesis-generating approach to assess whether Abatacept treatment is associated with coordinated changes in gene expression related to CD28-dependent signaling pathways at the population level.

Consistent with the known mechanism of abatacept, we observed a coordinated reduction in genes related to PI3K/AKT and MAPK signaling, which likely reflects attenuation of CD28-mediated costimulatory signaling in activated T cells. Given that PI3K/AKT pathway activation downstream of CD28 costimulation is a well-established feature of T cell activation, these findings are these findings are most consistent with reduced CD28-dependent signaling in T cells.

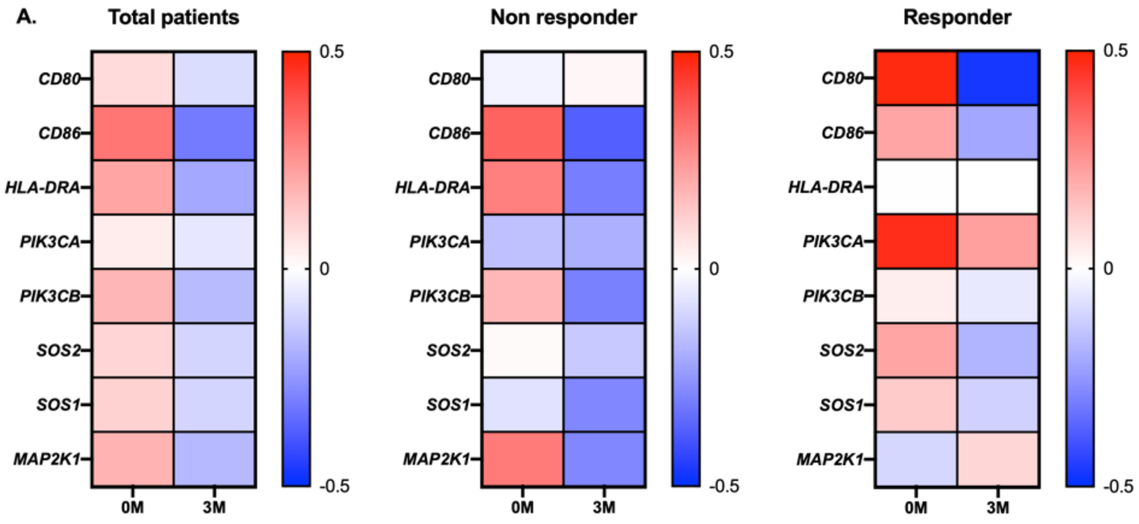

Figure R4. Reduced B7 molecules and CD28-related signaling molecules in RA patients after Abatacept treatment.

(A) Gene expression of B7 molecules and CD28 signaling-related molecules in RA patients before and after Abatacept treatment.

It is well established from both preclinical and clinical studies that CTLA-4 Ig treatment is associated with a reduction in regulatory T cells and Treg-associated suppressive markers. In patients with multiple sclerosis, switching from placebo to Abatacept treatment resulted in a time-dependent decrease in CD45RO$^+$ Foxp3$^+$ effector phenotype Treg cells in peripheral blood (J Immunol. 2019 Mar 1;202(5):1373-1382.). Notably, protein-level alterations of Treg markers following Abatacept treatment, including reductions in Foxp3, Helios, CD39, and CTLA-4–expressing Tregs, have also been reported in rheumatoid arthritis patients, which is consistent with our transcriptomic observations (BMC Immunol. 2013 Aug 5;14:34., Rheumatology (Oxford). 2022 Mar 2;61(3):1233-1242.).

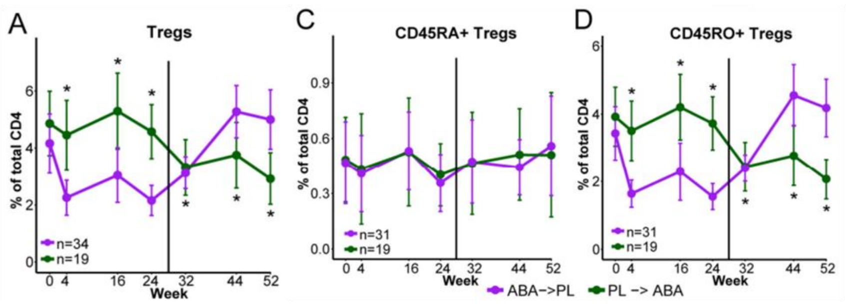

J Immunol. 2019 Mar 1;202(5):1373-1382.

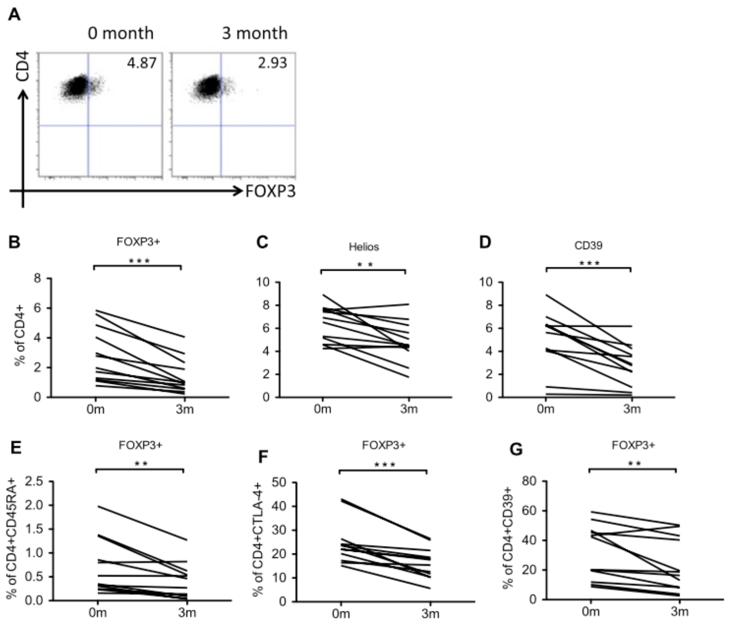

BMC Immunol. 2013 Aug 5;14:34.

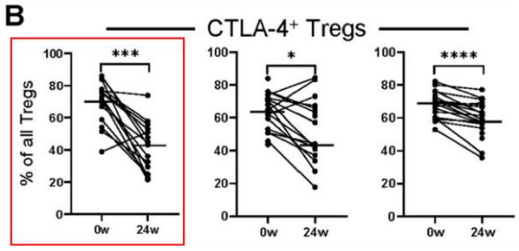

Rheumatology (Oxford). 2022 Mar 2;61(3):1233-1242.

In this context, the observed reduction in Treg-associated features and costimulatory molecules following abatacept treatment is not unexpected. A key distinction of our study, however, is that the transcriptomic data were derived from rheumatoid arthritis patients receiving clinically approved abatacept therapy and were analyzed by directly comparing responder and non-responder populations. This approach provides insight into how abatacept-mediated immune modulation differs according to clinical responsiveness, rather than merely reflecting global immunosuppression. This would be a separate independent further study to investigate.

In the revised manuscript, we have explicitly stated the limitations of bulk RNA-seq analysis without direct flow cytometric validation and complemented our findings with appropriate references that provide protein-level evidence for reduced Treg populations following abatacept treatment in Result section (line 149-156).

[Comment 3]

Figure 2. CTLA4.Ig boosts Treg differentiation in the presence of IL-2. This is not connected with the concept presented in the previous figure. Treg differentiation from naïve CD4 T cells is a different process than Treg maintenance in the setting of a systemic inflammatory disease. It has been proven before (Sakaguchi PNAS) that lack of CD28 increases Treg differentiation.

[Response 3]

We agree that there might be a confusion regarding the conceptual distinction between Treg maintenance in inflammatory settings (Fig 1) and Treg differentiation from naïve CD4 T cells (Fig 2). In chronic inflammatory settings, the reduction in Treg populations observed after Abatacept (CTLA-4 Ig) treatment is most likely attributable to impaired Treg stability resulting from costimulation blockade. However, our intention was not to equate these two phenomena, but rather to use them as sequential steps to address a single overarching question; whether the limitation of CTLA-4 Ig–mediated costimulation blockade on the Treg compartment can be functionally offset by IL-2 under conditions of reduced costimulatory signaling.

In this context, Fig 1 raises the problem of Treg loss following CTLA-4 Ig treatment both in mice and Abatacept-treated RA patients. Whereas Fig 2 addresses the biological hypothesis whether IL-2 can preferentially support Treg differentiation when costimulatory signaling is limited. Thus, while Treg stability and Treg differentiation are mechanistically distinct, they are conceptually linked as problem and potential solution.

As shown below in Fig 2, when costimulatory signaling is attenuated, IL-2 stimulation robustly enhances Foxp3 expression in CD4 T cells and promotes Treg differentiation under both Th17-polarizing and Treg-inducing conditions. These findings are consistent with previous reports by Sakaguchi and colleagues demonstrating that reduced CD28 signaling favors Treg differentiation. However, those studies primarily focused on phenotypic outcomes under Treg-polarizing conditions in Treg cells and did not address functional consequences in inflammatory disease settings or potential synergistic effects of costimulation blockade with IL-2.

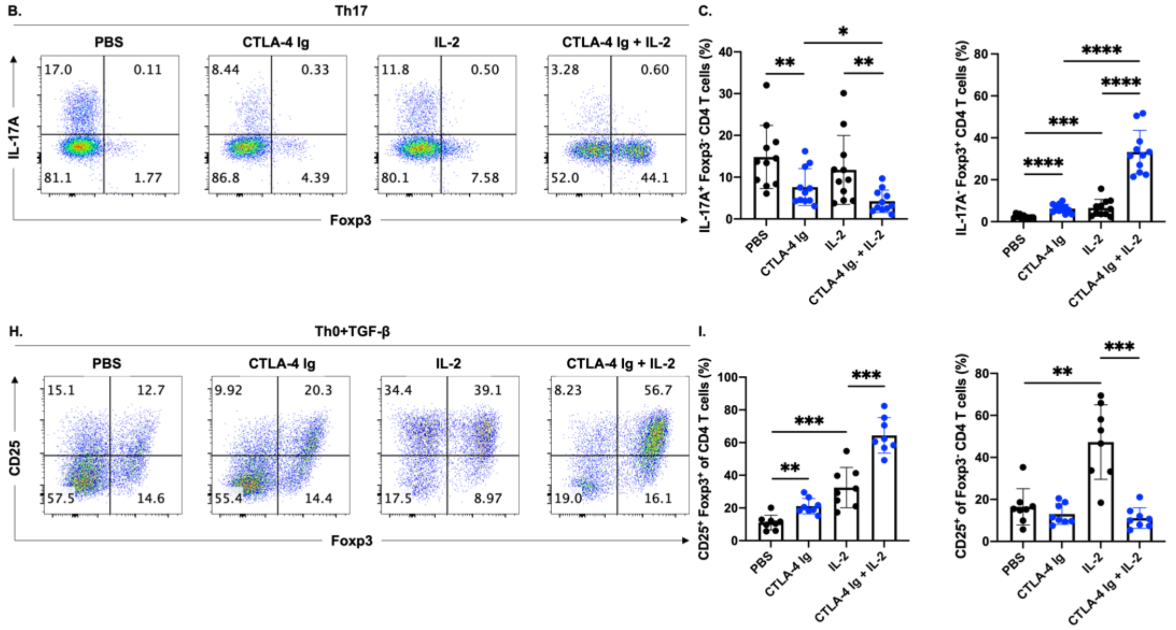

Revised Fig 2B-C, H-I. (B) Representative FACS dot plot showing IL-17A and Foxp3 expression in CD4 T cells cultured with IL-2, with or without CTLA-4 Ig in Th17 polarized condition. (C) Bar graph of IL-17A$^+$ Foxp3$^-$ effector CD4 T cells and IL-17A$^-$ Foxp3$^+$ iTreg cells. (H) Representative FACS dot plot showing CD25$^+$ Foxp3$^+$ proportion in CD4 T cells cultured with IL-2, with or without CTLA-4 Ig in Th0+TGF-β condition. (I) Bar graph indicating CD25$^+$ Foxp3$^+$ Treg population and CD25$^+$ Foxp3$^-$ effector T cell population.

We fully acknowledge prior work by Sakaguchi and colleagues (PNAS 2020) demonstrating that CD28 deprivation in conventional CD4 T cells induces hypomethylation of Foxp3 and Treg related genes and increased Foxp3 protein levels upon TGF-β and IL-2. In addition, CD28(-) iTreg cells exhibited enhanced regulatory function in DNFB-induced contact dermatitis model when they were transferred into the BALB/c mice.

In our study, we utilized CTLA-4 Ig for costimulation blockade combined with IL-2 synergistically induced Treg cells and showed therapeutic efficacy in EAE model which is in a similar line of conclusion. However, we focused on how costimulation loss would be beneficial for iTreg differentiation regarding IL-2 mediated downstream signaling. We investigated the intracellular signaling dynamics underlying this phenomenon, identifying a shift from PI3K/STAT3-dominant signaling toward STAT5 and TGF-β/Smad2/3–associated pathways under conditions of reduced costimulation. In addition, we suggest potential clinical use of CTLA-4 Ig in other

autoimmune diseases such as MS rather than just RA treatment (approved treatment) when combined to suboptimal dose of IL-2.

In this revised manuscript, we have clarified the rationale of Fig 2 in the Result section (lines 166-171). In addition, we have added corresponding sentences and references including Sakaguchi PNAS paper indicating costimulatory signaling and IL-2 signal are required for Treg maintenance and differentiation in Discussion section (line 391-412).

[Comment 4]

Figure 3. In vivo administration of IL-2 prevents loss of Tregs induced by CTLA4.Ig. This experiment is very important to prove the main message of the paper. However, the authors used a different mouse strain than in Figure 1... in Figure 1, the main effects of CTLA4.Ig (and allegedly of Treg depletion) was more severe and common relapses, but in this figure, they chose a strain with a non-relapsing disease. As in Figure 1, the mouse numbers seem very small and in some parameters the data shows a large dispersion. Importantly, there is no group with IL-2 and no CTLA4.Ig and the experimental design does not prove that IL-2 is restoring Treg numbers and phenotype by a specific mechanism.

[Response 4]

We thank the reviewer's point Fig. 3 is important experiments to prove our hypothesis and we agree to reviewer's criticize about reliability of in vivo model study and incomplete subgroup in EAE. To address reviewer's concern, we performed additional 2 independent sets of EAE experiments (finally 4 independent, n=11-19 per group) and included IL-2 alone group to make solid conclusion of CTLA-4 Ig and IL-2 effects on Treg restoration and synergistic effects on EAE treatment.

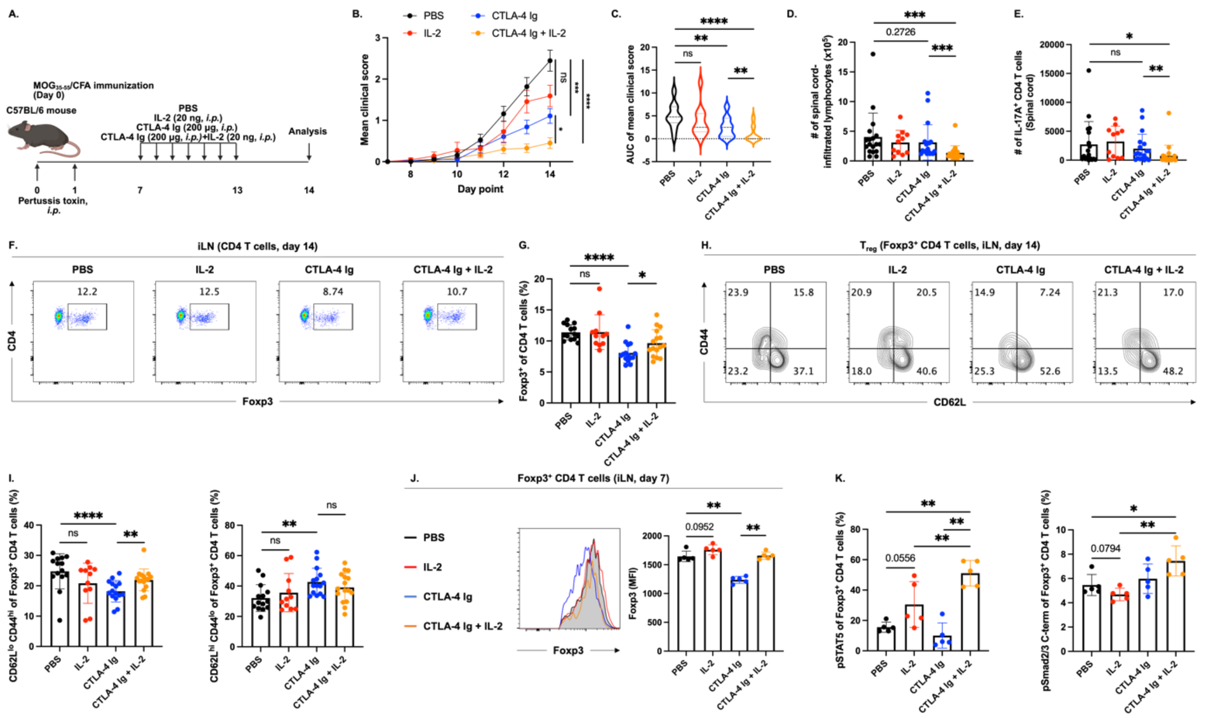

Revised Fig 3. Concomitant CTLA-4 Ig and IL-2 treatment synergistically ameliorates EAE disease.

(A-I) 10-weeks-old C57BL/6 female mice were subcutaneously immunized with MOG_{35-55} peptide (100 µg / flank) emulsified in CFA together with PTX. From day 7 after disease induction, mice received CTLA-4 Ig (200 µg), IL-2 (20 ng) or a combination of CTLA-4 Ig and IL-2 until day 13. (Representative data from 4 independent experiments) (A) Experimental scheme for inducing EAE. (B) Clinical disease score after EAE induction. (C) AUCs of total disease score after induction of EAE. (D) Cell number of spinal cord infiltrated lymphocyte analyzed by flow cytometry. (E) The number of IL-17A producing CD4 T cells in spinal cord. (F, G) Proportion of $Foxp3^+$ CD4 T cells in inguinal lymph node (iLN) analyzed by flow cytometry. Representative FACS dot plot (F) and bar graph (G). (H) Representative dot plot of naïve ($CD62L^{hi}$ $CD44^{lo}$) and effector/memory ($CD62L^{lo}$ $CD44^{hi}$) T cells in $Foxp3^+$ CD4 Treg cells of iLN analyzed by flow cytometry. (I) Bar graph indicating proportion of eTreg cells and cTreg cells. (J-L) 10-weeks-old C57BL/6 female mice were subcutaneously immunized with MOG_{35-55} peptide (100 µg / flank) emulsified in CFA with PTX. From day 0 after disease induction, mice received CTLA-4 Ig (200 µg), IL-2 (20 ng) or a combination of CTLA-4 Ig and IL-2 until day 7. (J) Representative histogram and bar graph of Foxp3 in $Foxp3^+$ CD4 T cells. (K) Bar graph indicating expression of pSTAT5 and pSmad2/3 C-term in $Foxp3^+$ CD4 T cells. Data are presented as mean ± S.E.M in (B) and mean ± S.D in remaining panels. Statistical significance was determined by Two-Way ANOVA in (B) and nonparametric Mann-Whitney test in remaining panels. ns = nonsignificant, *$p < 0.05$, **$p < 0.01$, ***$p<0.001$, ****$p<0.0001$.

In addition to phenotypic restoration of Treg populations, in vivo signaling analyses revealed intact IL-2–induced STAT5 phosphorylation and a significant increase in C-terminal Smad2/3 phosphorylation specifically in the CTLA-4 Ig and IL-2 combination group. These findings provide mechanistic support that the synergistic effect of CTLA-4 Ig and IL-2 is associated with preserved IL-2R–STAT5 signaling and enhanced TGF-β/Smad signaling in vivo.

In the revised manuscript, we updated Fig 3 with additional EAE experiments and IL-2 only group. Now the updated figure indicates 4 independent experiments with statistical significance of IL-2 effects on Treg restoring with in vivo signaling in Figure legend.

[Comment 5] Figure 4 is very relevant as it shows a possible mechanism. However, it is not completely explored, and no data demonstrates that this mechanism in place in the in vivo models presented.

Specifically:

a. The blot presented in Fig. 4A is very impressive, but the cumulative data does not look so strong, which makes me wonder if the blot is representative. In the pSTAT3 bar graph of Fig. 4B, is there a significant difference between PBS and IL-2 (first and third bars)? If not, it would be hard to sustain that IL-2 is really inducing STAT3 phosphorylation in a significant manner. Additionally, it would be more appropriate to use STAT3 as a loading control (instead of b-actin).

[Response a]

We thank the reviewer's comment that Fig.4 is very relevant and impressive results to explain possible molecular mechanism of CTLA-4 Ig and IL-2 treatment. To address reviewer's concern, we performed additional experiments to strengthen the reproducibility and statistical robustness of the signaling data shown in Figure 4.

1) First, to address the concern regarding representativeness, the immunoblot presented in Fig. 4A was selected from experiments that were independently repeated multiple times, all of which showed a consistent pattern. In these additional experiments, IL-2 treatment resulted in a statistically significant increase in STAT3 phosphorylation compared with PBS, and this IL-2–induced pSTAT3 was significantly reduced by CTLA-4 Ig under the same culture conditions. The cumulative quantification has been updated accordingly in the revised figure, and the number of independent experiments is now indicated in the figure legend.

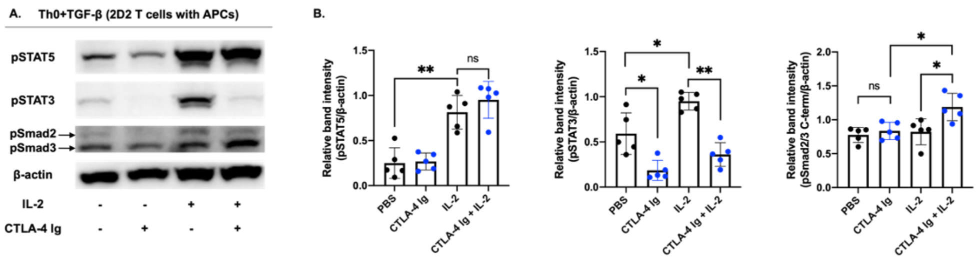

Revised Fig 4A and B. Naïve CD4 T cells sorted from 2D2 transgenic mice were cultured with irradiated APCs and stimulated by 20 μg/ml of MOG_{35-55} antigenic peptide with 2 ng/ml of TGF-β (Th0+ TGF-β) or TGF-β with 50 U/ml of IL-2 in presence of CTLA-4 Ig (0.5 μM) for 3 days. (A)

Immunoblotting of phosphorylation of STAT5, STAT3, C-terminal (C-term) of Smad2 and Smad3. (B) Representative change in band intensity of pSTAT5, pSTAT3 and pSmad2/3 C-term.

2) Regarding normalization, we performed additional immunoblotting for total STAT3 as suggested by the reviewer. Total STAT3 levels showed only modest variation across conditions relative to the changes observed in phosphorylated STAT3. Normalization of pSTAT3 to total STAT3 yielded the same overall conclusions as normalization to β-actin. For transparency, representative total STAT3 blots are provided in the answer figure. However, the primary quantification shown in the main panel remains based on β-actin normalization, as in the original submission.

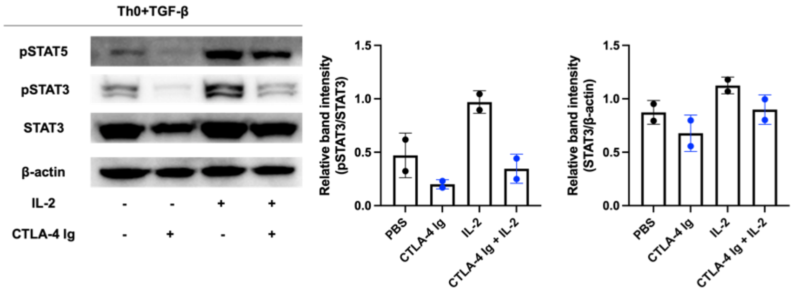

Fig. EV6. CTLA-4 Ig consistently reduces pSTAT3 levels when normalized to either STAT3 or β-actin. (A) Immunoblotting of phosphorylation of STAT5, STAT3 and total STAT3. (B) Relative band intensity of pSTAT3 normalized with STAT3 and STAT3 normalized with β-actin.

In the revised manuscript, Fig. 4B has been updated to include five independent western blot experiments with statistical analysis. In addition, pSTAT3 western blot analysis normalized to total STAT3 has been added to Fig. EV6 with corresponding sentences in Result section (line 245-247).

b. The authors claim that CTLA4.Ig decreases IL-2-induced STAT3 phosphorylation by inhibiting PI3K. However, they do not actually show that CTLA4.Ig inhibits PI3K. It would be important to show that.

[Response b]

We thank the reviewer for this comment. To address this point, we examined downstream PI3K signaling by measuring AKT phosphorylation by flow cytometry.

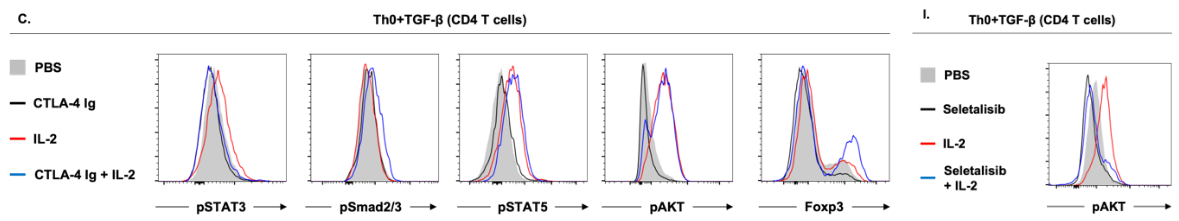

Revised Fig 4C and I. (C) Phosphorylation of STAT3, Smad2/3 C-term, STAT5 and AKT with expression of Foxp3 in CD4 T cells analyzed by flow cytometry. (I) Phosphorylation of AKT in CD4 T cells analyzed by flow cytometry.

IL-2 treatment robustly increased pAKT in CD4 T cells (red line in Fig. 4C), consistent with activation of both STAT5 and STAT3. In contrast, concomitant treatment with CTLA-4 Ig and IL-2 partially reduced pAKT and completely inhibited pSTAT3 while preserving pSTAT5 (blue line in Fig. 4C). Importantly, the combination group also showed increased pSmad2/3 levels with highly expression of Foxp3, confirming that the conclusions drawn from the western blot analysis are reproducible and that IL-2–driven PI3K activation under Th0 + TGF-β conditions is attenuated by CTLA-4 Ig.

Moreover, pharmacologic inhibition of PI3K using the selective PI3Kδ inhibitor seletalisib resulted in a pronounced reduction of pAKT accompanied by a corresponding decrease in pSTAT3 in splenocyte Th0 + TGF-β cultures. Collectively, these results support a model in which CTLA-4 Ig attenuates IL-2–induced STAT3 activation through inhibition of PI3K/AKT signaling.

In the revised manuscript, pAKT histogram plot is now added in Fig. 4C, 4I with relevance sentences in Results section (line 242-245; 273-274).

c. In Fig. 4G, the two pSTAT3 blots are discordant... is there a mistake?

[Response c]

We apologize for the confusion regarding Fig 4G, which shows a strong pSTAT3 signal in solenocytes stimulated with anti-CD3 antibody and TGF-β for 3 days. These experiments were repeated multiple independent times, during which some variability in the baseline pSTAT3 signal was observed in the control group, ranging from relatively strong to weak levels. However, the increase in pSTAT3

following IL-2 treatment in addition to anti-CD3 antibody and TGF-β was consistent across most experiments. We inadvertently selected a representative image from the single replicate that showed a weaker (marginal) response by IL-2, as the base line control signal in that replicate was too strong.

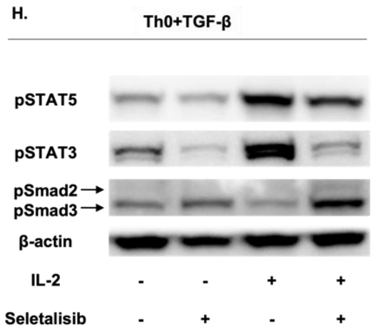

Revised Fig 4G to 4H. (H) Immunoblotting of phosphorylation of STAT5, STAT3 and Smad2/3 C-term.

We have now revised Fig. 4H to include the appropriate experimental data.

[Comment 6]

Figure 5 is also hard to interpret. I do not think it proves any point.

[Response 6]

We apology for the reviewer to interpret Fig. 5 that we intended to demonstrate. We would like to confirm our finding of CTLA-4 Ig and IL-2 cotreatment effects on human samples including MS (Multiple Sclerosis) patients and non-IDD (non-inflammatory demyelinating disease) samples. To address reviewer’s concern, we decided to add experimental scheme and gating strategy with reorganized figure orientation and updated expanded figure.

First, to further improve clarity and address the reviewer’s concern regarding interpretability, we have added a schematic overview illustrating the experimental design and flow cytometric gating strategy used for Figure 5. This schematic depicts the source of human PBMCs, the ex vivo culture conditions, and the sequential gating strategy applied (lymphocytes → live cells → $CD4^+$ T cells → $Foxp3^+$ and $Foxp3^-$ subsets), as well as the quadrant-based analysis used to assess CD25 expression patterns. By explicitly presenting the experimental workflow and gating logic, we aim to facilitate interpretation of Fig 5 and to clarify how Treg-associated phenotypes were defined and quantified. This schematic has been incorporated into the revised figure to enhance transparency and reproducibility.

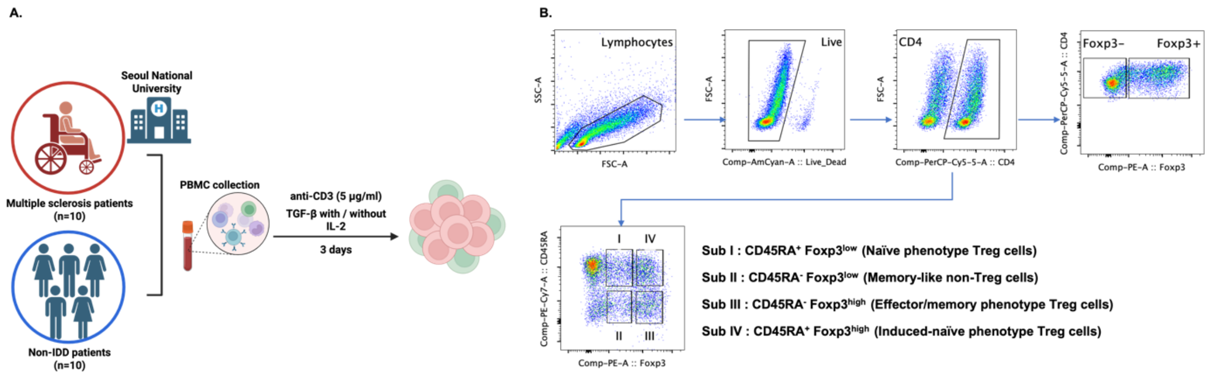

Revised Fig 5A and B. (A) Schematic overview of the experimental design and (B) gating strategy for Treg subset analysis. Naïve and effector/memory Treg cells were analyzed in 4 subpopulations; $CD45RA^+$ $Foxp3^{low}$ Treg cells: Sub I; $CD45RA^-$ $Foxp3^{low}$ Treg: Sub II; $CD45RA^-$ $Foxp3^{high}$ Treg cells: Sub III; $CD45RA^+$ $Foxp3^{high}$ Treg cells: Sub IV.

In addition, we excluded Sub I and Sub II from Fig 5 to Fig. EV9 to improve readability of data flow.

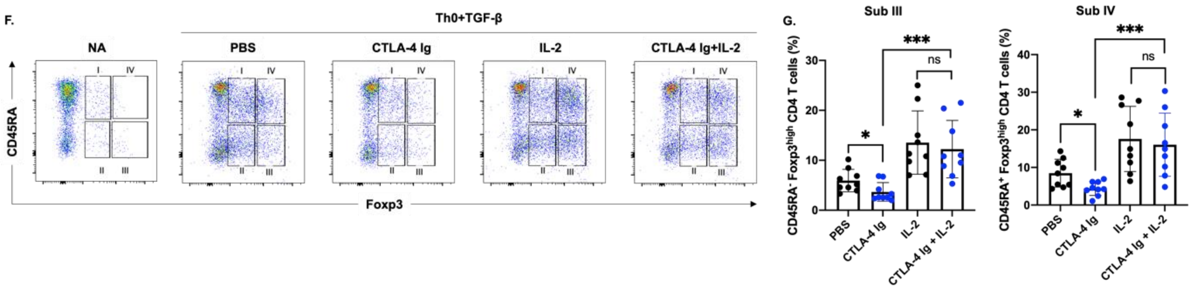

Revised Fig 5F and G. (F, G) Representative FACS dot plot (F) and bar graph (G) of III (Effector/memory phenotype Treg cells) and IV (induced-naïve phenotype Treg cells) sub-population of Treg cells.

We also added CD25 expression histogram data in T_{eff} (Foxp3$^-$ CD4 T cells) to confirm CTLA-4 Ig treatment inhibits T cell activation either with or without IL-2.

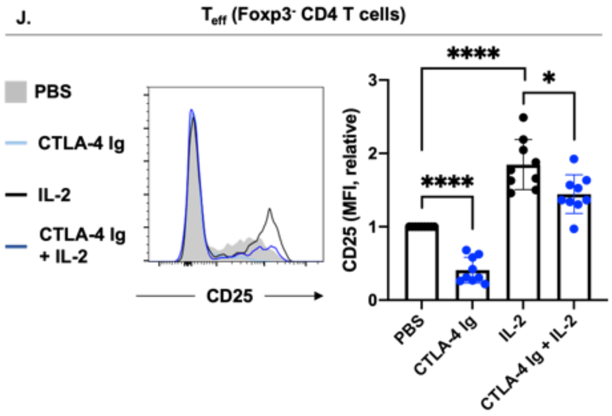

Revised Fig. 5J. Representative histogram and bar graph of CD25 expression in Foxp3$^-$ effector CD4 T cells.

Lastly, we incorporated previously available data from the non-IDD patient group in Fig. EV10, which served as a non-inflammatory comparator cohort for MS. Analysis of this cohort revealed response patterns consistent with those observed in MS patients.

In the revised manuscript, we have updated Fig 5 with experimental scheme and gating strategy to better visualization and clarification. We also added non-IDD control patient sample experiments in Fig EV10.

27th Mar 2026

Dear Prof. Choi,

Thank you for the submission of your revised manuscript to EMBO Molecular Medicine. Unfortunately, two original referees were unavailable to re-review your manuscript. Therefore, we had to invite an additional reviewer to be able to make an informed decision. I am pleased to inform you that we will be able to accept your manuscript pending the following final amendments:

1) Please address referee #4 minor suggestion.
2) Figures:
- Please submit main Figures and EV Figures as individual, high resolution figure files. Please place figure legends for main and EV figures at the end of the main manuscript file. Add the heading Figure Legends for the main figures and for the EV Figures: "Expanded View Figure Legends". Please check "Author Guidelines" for more information:
https://www.embopress.org/page/journal/17574684/authorguide#figureformat
https://www.embopress.org/page/journal/17574684/authorguide#expandedview
- We note that FACS data presented in Figure 2B - Th17 appear the same as in Figure 5A - Th17 in your published manuscript Lee et al., 2023 (DOI: 10.3389/fimmu.2023.1233514). Please clarify and if this is intentional the manuscript must be cited in the figure legend and in the reference list.
3) In the main manuscript file, please do the following:
- Please address all comments suggested by our data editors listed below:
o Figure legends:
1. Please note that the exact p values are not provided in the legends of figures 1B, C, D, E, G, M; 2C, D, F, G, I, J, K; 3B, C, D, E, G, I, J, K; 4B, F, G, K; 5D, E, G, I, J; EV1 C, D, F, G, H, K; EV2 A, EV3 C, EV4 B, C; EV5 A, EV8 C, EV9 C, EV10 B, C, E, G, H; S1C, S2 C, E, H, J.
2. Please note that information related to n is missing in the legends of figures 1B, C, D, E, G; 2C, D, F, G, I, J, K; 3B, C, D, E, G, I, J, K; 4B, F, G, K; 5D, E, G, I, J; EV 3 C, EV4 B-D; EV5 A; EV6 B, EV8 C, EV10 B, C, E, G, H; S1 C, S2 C, E, H, J.
- Add callouts for Fig 3J, Fig 5G and for panels of Fig EV4, EV5 and EV10.
- In Methods, add a paragraph describing the analysis of the published dataset 8250013. Dataset should be adequately cited here incl. the publications.
- In Methods, provide the antibody dilutions that were used for each antibody.
- In Methods, provide the statement that informed consent was obtained from all human subjects and confirm that the experiments conformed to the principles set out in the WMA Declaration of Helsinki and the Department of Health and Human Services Belmont Report.
- In Methods, statistical paragraph should reflect all information that you have filled in the Authors Checklist, especially regarding randomization, blinding, replication.
- In Methods, add a dedicated "Graphics" section following this format (remove BioRender reference from Acknowledgements):
Graphics:
(some of the... OR Figure #... OR synopsis) Graphics were created with BioRender.com.
- Indicate in legends number and nature of replicates and exact p= values, not a range, along with the statistical test used. To keep the figures "clear" some authors found providing an Appendix table Sx with all exact p-values preferable. You are welcome to do this if you want to.
- Please provide Reagents and Tools Table and uploaded it as a separate file. Structured Methods section includes Reagents and Tools Table followed by a Methods and Protocols section. More information on how to adhere to this format as well as downloadable templates (.docx) for the Reagents and Tools Table can be found in our author guidelines:
https://www.embopress.org/page/journal/17574684/authorguide#structuredmethods
An example of a paper with Structured Methods can be found here:
https://www.embopress.org/doi/full/10.1038/s44320-024-00037-6#sec-4
- Rename "Competing Interest Statement" to "Disclosure Statement & Competing Interests". We updated our journal's competing interests policy in January 2022 and request authors to consider both actual and perceived competing interests. Please review the policy https://www.embopress.org/competing-interests and update your competing interests if necessary.
- Author contributions: Please remove it from the manuscript and specify author contributions in our submission system. CRediT has replaced the traditional author contributions section because it offers a systematic machine-readable author contributions format that allows for more effective research assessment. You are encouraged to use the free text boxes beneath each contributing author's name to add specific details on the author's contribution. More information is available in our guide to authors:
https://www.embopress.org/page/journal/17574684/authorguide#authorshipguidelines
- Remove the appendix figure and table legends.
- Remove duplicate title page on p. 50.
- Data availability statement should contain information only for the raw data generated in this study and deposited in public databases. Please remove information about data generated in other studies and cite it and the corresponding publication at an appropriate place in Methods. If no data are deposited in public repositories, please replace current text with the following sentence: This study includes no data deposited in external repositories.

- Correct the reference citation in the reference list. Where there are more than 10 authors on a paper, 10 will be listed, followed by "et al.". Also, please remove DOIs for published articles. Please check "Author Guidelines" for more information. https://www.embopress.org/page/journal/17574684/authorguide#referencesformat
4) Appendix: Please remove yellow highlights and add a table of content with page numbers on the title page. Please increase the font of the figure and table legends and add the figures and the table in higher resolution. Remove the duplicated file for Appendix Table S1.
5) Dataset: Add a legend in a separate tab.
6) Funding: Please make sure that information about all sources of funding are complete in both our submission system and in the manuscript. Currently, RS-2025-00520647 is missing in the manuscript text and RS-2025-02232977 is missing in our system. Please correct.
7) The Paper Explained: Please provide "The Paper Explained" and add it to the main manuscript text. Please check "Author Guidelines" for more information. https://www.embopress.org/page/journal/17574684/authorguide#researcharticleguide
8) Synopsis: Every published paper now includes a 'Synopsis' to further enhance discoverability. Synopses are displayed on the journal webpage and are freely accessible to all readers. They include separate synopsis image and synopsis text.
- Synopsis image: Please provide a visual abstract as a high-resolution jpeg file Please provide a visual abstract as a high-resolution jpeg file 550 pixels wide x 300 pixels high to illustrate your article.
- Synopsis text: Please provide a short standfirst (maximum of 300 characters, including space) as well as 2-5 one sentence bullet points that summarise the paper as a .doc file. Please write the bullet points to summarise the key NEW findings. They should be designed to be complementary to the abstract - i.e. not repeat the same text. We encourage inclusion of key acronyms and quantitative information (maximum of 30 words / bullet point). Please use the passive voice.
- Please check your synopsis text and image before submission with your revised manuscript. Please be aware that in the proof stage minor corrections only are allowed (e.g., typos).
9) As part of the EMBO Publications transparent editorial process (see our Editorial at http://embomolmed.embopress.org/content/2/9/329), EMBO Molecular Medicine will publish online a Review Process File (RPF) to accompany accepted manuscripts. This file will be published in conjunction with your paper and will include the anonymous referee reports, your point-by-point response and all pertinent correspondence relating to the manuscript. Let us if you want to remove or not any figures from it prior to publication. Please note that the Authors checklist will be published at the end of the RPF.
10) Please provide a point-by-point letter INCLUDING my comments as well as the reviewer's reports and your detailed responses (as Word file).

I look forward to reading a new revised version of your manuscript as soon as possible.

Yours sincerely,

Zeljko Durdevic

Zeljko Durdevic
Senior Editor
EMBO Molecular Medicine

*** Instructions to submit your revised manuscript ***

*** PLEASE NOTE *** As part of the EMBO Publications transparent editorial process initiative (see our Editorial at https://www.embopress.org/doi/pdf/10.1002/emmm.201000094), EMBO Molecular Medicine will publish online a Review Process File to accompany accepted manuscripts.

In the event of acceptance, this file will be published in conjunction with your paper and will include the anonymous referee reports, your point-by-point response and all pertinent correspondence relating to the manuscript. If you do NOT want this file to be published, please inform the editorial office at contact@embomolmed.org.

*****

When preparing your revised manuscript, please refer to our guidelines: https://link.springer.com/journal/44321/submission-

guidelines#cms-Revised-submissions. We perform an initial quality control of all revised manuscripts before re-review; failure to include requested items will delay the evaluation of your revision.

We require:

1) A .docx formatted version of the manuscript text (including legends for main figures, EV figures and tables). Please make sure that the changes are highlighted to be clearly visible.

2) Individual production quality figure files as .eps, .tif, .jpg (one file per figure). For guidance, download the 'Figure Guide PDF': https://media.springernature.com/original/springer-cms/rest/v1/content/27825798/data/v1.

3) A .docx formatted letter INCLUDING the reviewers' reports and your detailed point-by-point responses to their comments. As part of the EMBO Press transparent editorial process, the point-by-point response is part of the Review Process File (RPF), which will be published alongside your paper.

4) A complete author checklist, which you can download from our author guidelines. Please insert information in the checklist that is also reflected in the manuscript. The completed author checklist will also be part of the RPF.

5) Please note that all corresponding authors are required to supply an ORCID ID for their name upon submission of a revised manuscript.

6) It is mandatory to include a 'Data Availability' section after the Materials and Methods. Before submitting your revision, primary datasets produced in this study need to be deposited in an appropriate public database, and the accession numbers and database listed under 'Data Availability'. Please remember to provide a reviewer password if the datasets are not yet public.

In case you have no data that requires deposition in a public database, please state so in this section. Note that the Data Availability Section is restricted to new primary data that are part of this study.

7) For data quantification: please specify the name of the statistical test used to generate error bars and P values, the number (n) of independent experiments (specify technical or biological replicates) underlying each data point and the test used to calculate p-values in each figure legend. The figure legends should contain a basic description of n, P and the test applied. Graphs must include a description of the bars and the error bars (s.d., s.e.m.).

8) At EMBO Press we ask authors to provide source data for the main manuscript figures. You will receive a separate email with instructions for providing source data with your revised manuscript, including how to upload and organize the files.

9) Our journal encourages inclusion of *data citations in the reference list* to directly cite datasets that were re-used and obtained from public databases. Data citations in the article text are distinct from normal bibliographical citations and should directly link to the database records from which the data can be accessed. In the main text, data citations are formatted as follows: "Data ref: Smith et al, 2001" or "Data ref: NCBI Sequence Read Archive PRJNA342805, 2017". In the Reference list, data citations must be labeled with "[DATASET]". A data reference must provide the database name, accession number/identifiers and a resolvable link to the landing page from which the data can be accessed at the end of the reference.

10) We replaced Supplementary Information with Expanded View (EV) Figures and Tables that are collapsible/expandable online. A maximum of 5 EV Figures can be typeset. EV Figures should be cited as 'Figure EV1, Figure EV2" etc... in the text and their respective legends should be included in the main text after the legends of regular figures.

- For the figures that you do NOT wish to display as Expanded View figures, they should be bundled together with their legends in a single PDF file called *Appendix*, which should start with a short Table of Content. Appendix figures should be referred to in the main text as: "Appendix Figure S1, Appendix Figure S2" etc.

- Additional Tables/Datasets should be labeled and referred to as Table EV1, Dataset EV1, etc. Legends have to be provided in a separate tab in case of .xls files. Alternatively, the legend can be supplied as a separate text file (README) and zipped together with the Table/Dataset file.

11) The paper explained: EMBO Molecular Medicine articles are accompanied by a summary of the articles to emphasize the major findings in the paper and their medical implications for the non-specialist reader. Please provide a draft summary of your article highlighting

- the medical issue you are addressing,

- the results obtained and

- their clinical impact.

This may be edited to ensure that readers understand the significance and context of the research. Please refer to any of our published articles for an example.

12) Author contributions: You will be asked to provide CRediT (Contributor Role Taxonomy) terms in the submission system. These replace a narrative author contribution section in the manuscript.

13) A Conflict of Interest statement should be provided in the main text.

14) Every published paper includes a 'Synopsis' to further enhance discoverability. Synopses are displayed on the journal webpage and are freely accessible to all readers. They include a short stand first (maximum of 300 characters, including space) as well as 2-5 one-sentences bullet points that summarizes the paper. Please write the bullet points to summarize the key NEW findings. They should be designed to be complementary to the abstract - i.e. not repeat the same text. We encourage inclusion of key acronyms and quantitative information (maximum of 30 words / bullet point). Please use the passive voice. Please attach these in a separate file or send them by email, we will incorporate them accordingly.

Please also suggest a striking image or visual abstract to illustrate your article as a PNG file 550 px wide x 300 px high.

15) Include a Reagents and Tools Table as part of the Methods section, which can be downloaded from our author guidelines.

EMBO Molecular Medicine has a "scooping protection" policy, whereby similar findings that are published by others during review or revision are not a criterion for rejection. Should you decide to submit a revised version, I do ask that you get in touch after three months if you have not completed it, to update us on the status.

*Additional important information regarding Figures

Each figure should be given in a separate file and should have the following resolution:
Graphs 800-1,200 DPI
Photos 400-800 DPI
Colour (only CMYK) 300-400 DPI"

Figures are not edited by the production team. All lettering should be the same size and style; figure panels should be indicated by capital letters (A, B, C etc). Gridlines are not allowed except for log plots. Figures should be numbered in the order of their appearance in the text with Arabic numerals. Each Figure must have a separate legend and a caption is needed for each panel.

*Additional important information regarding figures and illustrations can be found at
https://media.springernature.com/original/springer-cms/rest/v1/content/27825798/data/v1

***** Reviewer's comments *****

Referee #1 (Comments on Novelty/Model System for Author):

Models are adequate. No concerns

Referee #1 (Remarks for Author):

Nam et al. have adequately addressed my prior critiques.

Referee #4 (Remarks for Author):

In this revision I believe that authors have adequately addressed the prior reviewer comments. One minor issue which could be improved is the response to Review 1, comment 2. Absolute number should be calculated in reference to a cell count of the original sample, not to a count from the flow cytometer collection.

1) Please address referee #4 minor suggestion.

***** Reviewer's comments *****

Referee #1 (Comments on Novelty/Model System for Author):

Models are adequate. No concerns

Referee #1 (Remarks for Author):

Nam et al. have adequately addressed my prior critiques.

Referee #4 (Remarks for Author):

[Comment 1]

In this revision I believe that authors have adequately addressed the prior reviewer comments. One minor issue which could be improved is the response to Review 1, comment 2. Absolute number should be calculated in reference to a cell count of the original sample, not to a count from the flow cytometer collection.

[Response 1]

We agree with the reviewer that accurate determination of absolute cell numbers requires direct measurement of total cell counts from the original culture samples. In our in vitro 2D2 co-culture experiments (Figure 2), however, we did not measure total cell numbers when harvesting cells and collecting culture media for subsequent analyses. Therefore, we were unable to provide absolute cell numbers as originally requested by the reviewer.

Instead, we reanalyzed all data and present them as total frequency, which we believe better reflects the relative abundance of the target cell populations recorded by flow cytometry (Figure 2D and J). The main difference between the revised and previous versions is observed in the CTLA-4-Ig–treated group, which now shows a significant reduction in the total frequency of Treg cells, due to a decrease in the number of live cells following CTLA-4-Ig treatment. Therefore, although this does not fully replace direct absolute cell counting, we believe that

presenting total frequency provides an alternative way to address the reviewer's concern.

8th Apr 2026

Dear Prof. Choi,

We are pleased to inform you that your manuscript is accepted for publication and is now being sent to our publisher to be included in the next available issue of EMBO Molecular Medicine.

Your manuscript will be processed for publication by EMBO Press. It will be copy edited and you will receive page proofs prior to publication. Please note that you will be contacted by Springer Nature Author Services to complete licensing and payment information.

You may qualify for financial assistance for your publication charges - either via a Springer Nature fully open access agreement or an EMBO initiative. Check your eligibility: https://link.springer.com/journal/44321/how-to-publish-with-us

Should you be planning a Press Release on your article, please get in contact with embo_production@springernature.com as early as possible in order to coordinate publication and release dates.

If you have any questions, please do not hesitate to contact the Editorial Office. Thank you for your contribution to EMBO Molecular Medicine.

Yours sincerely,
Zeljko Durdevic

Zeljko Durdevic
Senior Editor
EMBO Molecular Medicine

>>> Please note that it is EMBO Molecular Medicine policy for the transcript of the editorial process (containing referee reports and your response letter) to be published as an online supplement to each paper. If you do NOT want this, you will need to inform the Editorial Office via email immediately. More information is available here: https://link.springer.com/partners/embo-press/editorial-policies#Peer%20review

EMBO Press Author Checklist

Corresponding Author Name: JE-MIN CHOI	
Journal Submitted to: Costimulation loss enhances IL-2-driven Treg generation by PI3K-STAT	
Manuscript Number: EMM-2025-21975	

USEFUL LINKS FOR COMPLETING THIS FORM
The EMBO Journal - Author Guidelines
EMBO Reports - Author Guidelines
Molecular Systems Biology - Author Guidelines
EMBO Molecular Medicine - Author Guidelines

Reporting Checklist for Life Science Articles (updated January
This checklist is adapted from Materials Design Analysis Reporting (MDAR) Checklist for Authors. MDAR establishes a minimum set of requirements in transparent reporting in the life sciences (see Statement of Task: 10.31222/osf.io/9sm4x). Please follow the journal's guidelines in preparing your
Please note that a copy of this checklist will be published alongside your article.

Abridged guidelines for figures
1. Data
The data shown in figures should satisfy the following conditions:
- the data were obtained and processed according to the field's best practice and are presented to reflect the results of the experiments in an accurate and unbiased manner.
- ideally, figure panels should include only measurements that are directly comparable to each other and obtained with the same assay.
- plots include clearly labeled error bars for independent experiments and sample sizes. Unless justified, error bars should not be shown for technical
- if n<5, the individual data points from each experiment should be plotted. Any statistical test employed should be justified.
- Source Data should be included to report the data underlying figures according to the guidelines set out in the authorship guidelines on Data

2. Captions
Each figure caption should contain the following information, for each panel where they are relevant:
- a specification of the experimental system investigated (eg cell line, species name).
- the assay(s) and method(s) used to carry out the reported observations and measurements.
- an explicit mention of the biological and chemical entity(ies) that are being measured.
- an explicit mention of the biological and chemical entity(ies) that are altered/varied/perturbed in a controlled manner.
- the exact sample size (n) for each experimental group/condition, given as a number, not a range;
- a description of the sample collection allowing the reader to understand whether the samples represent technical or biological replicates (including how many animals, litters, cultures, etc.).
- a statement of how many times the experiment shown was independently replicated in the laboratory.
- definitions of statistical methods and measures:
  - common tests, such as t-test (please specify whether paired vs. unpaired), simple χ2 tests, Wilcoxon and Mann-Whitney tests, can be unambiguously identified by name only, but more complex techniques should be described in the methods section;
  - are tests one-sided or two-sided?
  - are there adjustments for multiple comparisons?
  - exact statistical test results, e.g., P values = x but not P values < x;
  - definition of 'center values' as median or average;
  - definition of error bars as s.d. or s.e.m.

Please complete ALL of the questions below.
Select "Not Applicable" only when the requested information is not relevant for your study.

Materials

Newly Created Materials	Information included in the manuscript?	In which section is the information available? (Reagents and Tools Table, Materials and Methods, Figures, Data Availability Section)
New materials and reagents need to be available; do any restrictions apply?	Not Applicable	

Antibodies	Information included in the manuscript?	In which section is the information available? (Reagents and Tools Table, Materials and Methods, Figures, Data Availability Section)
For **antibodies** provide the following information:<br>- Commercial antibodies: RRID (if possible) or supplier name, catalogue number and or/clone number<br>- Non-commercial: RRID or citation	Yes	Materials and Methods

DNA and RNA sequences	Information included in the manuscript?	In which section is the information available? (Reagents and Tools Table, Materials and Methods, Figures, Data Availability Section)
**Short novel DNA or RNA including primers, probes**: provide the sequences.	Not Applicable	

Cell materials	Information included in the manuscript?	In which section is the information available? (Reagents and Tools Table, Materials and Methods, Figures, Data Availability Section)
**Cell lines:** Provide species information, strain. Provide accession number in repository **OR** supplier name, catalog number, clone number, and/**OR** RRID.	Not Applicable	
**Primary cultures:** Provide species, strain, sex of origin, genetic modification status.	Yes	Materials and Methods. Figures
Report if the cell lines were recently **authenticated** (e.g., by STR profiling) and tested for mycoplasma contamination.	Not Applicable	

Experimental animals	Information included in the manuscript?	In which section is the information available? (Reagents and Tools Table, Materials and Methods, Figures, Data Availability Section)
**Laboratory animals or Model organisms:** Provide species, strain, sex, age, genetic modification status. Provide accession number in repository **OR** supplier name, catalog number, clone number, **OR** RRID.	Yes	Materials and Methods
**Animal observed in or captured from the field:** Provide species, sex, and age where possible.	Not Applicable	
Please detail **housing and husbandry conditions**.	Yes	Materials and Methods

Plants and microbes	Information included in the manuscript?	In which section is the information available? (Reagents and Tools Table, Materials and Methods, Figures, Data Availability Section)
**Plants:** provide species and strain, ecotype and cultivar where relevant, unique accession number if available, and source (including location for collected wild specimens).	Not Applicable	
**Microbes:** provide species and strain, unique accession number if available, and source.	Not Applicable	

Human research participants	Information included in the manuscript?	In which section is the information available? (Reagents and Tools Table, Materials and Methods, Figures, Data Availability Section)
If collected and within the bounds of privacy constraints report on age, sex and gender or ethnicity for all study participants.	Yes	Appendix Table S1

Core facilities	Information included in the manuscript?	In which section is the information available? (Reagents and Tools Table, Materials and Methods, Figures, Data Availability Section)
If your work benefited from core facilities, was their service mentioned in the acknowledgments section?	Not Applicable	

Design

Study protocol	Information included in the manuscript?	In which section is the information available? (Reagents and Tools Table, Materials and Methods, Figures, Data Availability Section)
If study protocol has been **pre-registered, provide DOI in the manuscript**. For clinical trials, provide the trial registration number **OR** cite DOI.	Not Applicable	
Report the **clinical trial registration number** (at ClinicalTrials.gov or equivalent), where applicable.	Not Applicable	

Laboratory protocol	Information included in the manuscript?	In which section is the information available? (Reagents and Tools Table, Materials and Methods, Figures, Data Availability Section)
Provide DOI OR other citation details if **external detailed step-by-step protocols** are available.	Not Applicable	

Experimental study design and statistics	Information included in the manuscript?	In which section is the information available? (Reagents and Tools Table, Materials and Methods, Figures, Data Availability Section)
Include a statement about **sample size** estimate even if no statistical methods were used.	Not Applicable	
Were any steps taken to minimize the effects of subjective bias when allocating animals/samples to treatment (e.g. **randomization procedure**)? If yes, have they been described?	Not Applicable	
Include a statement about **blinding** even if no blinding was done.	Yes	Materials and Methods
Describe **inclusion/exclusion criteria** if samples or animals were excluded from the analysis. Were the criteria pre-established?<br><br>If sample or data points were omitted from analysis, report if this was due to attrition or intentional exclusion and provide justification.	Not Applicable	
For every figure, are **statistical tests** justified as appropriate? Do the data meet the assumptions of the tests (e.g., normal distribution)? Describe any methods used to assess it. Is there an estimate of variation within each group of data? Is the variance similar between the groups that are being statistically compared?	Yes	Figures, Materials and Methods

Sample definition and in-laboratory replication	Information included in the manuscript?	In which section is the information available? (Reagents and Tools Table, Materials and Methods, Figures, Data Availability Section)
In the figure legends: state number of times the experiment was **replicated** in laboratory.	Yes	Figure legends
In the figure legends: define whether data describe **technical or biological replicates**.	Yes	Figure legends

Ethics

Ethics	Information included in the manuscript?	In which section is the information available? (Reagents and Tools Table, Materials and Methods, Figures, Data Availability Section)
Studies involving **human participants**: State details of **authority granting ethics approval** (IRB or equivalent committee(s), provide reference number for approval.	Yes	Ethics statement
Studies involving **human participants**: Include a statement confirming that **informed consent** was obtained from all subjects and that the experiments conformed to the principles set out in the WMA Declaration of Helsinki and the Department of Health and Human Services Belmont Report.	Not Applicable	
Studies involving **human participants: For** publication of **patient photos**, include a statement confirming that consent to publish was obtained.	Not Applicable	
Studies involving experimental **animals**: State details of **authority granting ethics approval** (IRB or equivalent committee(s), provide reference number for approval. Include a statement of compliance with ethical regulations.	Yes	Ethics statement
Studies involving **specimen and field samples:** State if relevant **permits** obtained, provide details of authority approving study; if none were required, explain why.	Not Applicable	

Dual Use Research of Concern (DURC)	Information included in the manuscript?	In which section is the information available? (Reagents and Tools Table, Materials and Methods, Figures, Data Availability Section)
Could your study fall under dual use research restrictions? Please check biosecurity documents and list of **select agents and toxins** (CDC): https://www.selectagents.gov/sat/list.htm	Not Applicable	
If you used a select agent, is the security level of the lab appropriate and reported in the manuscript?	Not Applicable	
If a study is subject to dual use research of concern regulations, is the name of the **authority granting approval and reference number** for the regulatory approval provided in the manuscript?	Not Applicable	

Reporting

The MDAR framework recommends adoption of discipline-specific guidelines, established and endorsed through community initiatives. Journals have their own policy about requiring specific guidelines and recommendations to complement MDAR.

Adherence to community standards	Information included in the manuscript?	In which section is the information available? (Reagents and Tools Table, Materials and Methods, Figures, Data Availability Section)
State if relevant guidelines or checklists (e.g., **ICMJE, MIBBI, ARRIVE, PRISMA**) have been followed or provided.	Not Applicable	
For **tumor marker prognostic studies,** we recommend that you follow the **REMARK** reporting guidelines (see link list at top right). See author guidelines, under 'Reporting Guidelines'. Please confirm you have followed these guidelines.	Not Applicable	
For **phase II and III randomized controlled trials**, please refer to the **CONSORT** flow diagram (see link list at top right) and submit the CONSORT checklist (see link list at top right) with your submission. See author guidelines, under 'Reporting Guidelines'. Please confirm you have submitted this list.	Not Applicable	

Data Availability

Data availability	Information included in the manuscript?	In which section is the information available? (Reagents and Tools Table, Materials and Methods, Figures, Data Availability Section)
Have **primary datasets** been deposited according to the journal's guidelines (see 'Data Deposition' section) and the respective accession numbers provided in the Data Availability Section?	Not Applicable	
Were **human clinical and genomic datasets** deposited in a public access-controlled repository in accordance to ethical obligations to the patients and to the applicable consent agreement?	Yes	Data Availability Section
Are **computational models** that are central and integral to a study available without restrictions in a machine-readable form? Were the relevant accession numbers or links provided?	Not Applicable	
If publicly available data were reused, provide the respective **data citations in the reference list**.	Yes	References, Data Availability Section
